# Systematic intensive therapy in addition to continuous glucose monitoring in adults with type 1 diabetes: a multicentre, open-label, randomised controlled trial

**DOI:** 10.1016/j.lanepe.2025.101485

**Published:** 2025-10-16

**Authors:** Arndís F. Ólafsdóttir, Kari-Anne Sveen, Magnus Wijkman, Sara Hallström, Per-Henrik Nilsson, Sofia Sterner Isaksson, Helene Holmer, Marie Ekström, Henrik Imberg, Marcus Lind

**Affiliations:** a Department of Medicine, NU Hospital Group, Uddevalla, Sweden; b Department of Molecular and Clinical Medicine, Institute of Medicine, Sahlgrenska Academy, University of Gothenburg, Gothenburg, Sweden; c Department of Endocrinology, Morbid Obesity and Preventive Medicine, Oslo University Hospital, Oslo, Norway; d Department of Health, Medicine and Caring Sciences and Department of Internal Medicine, Linköping University, Norrköping, Sweden; e Department of Internal Medicine, Sahlgrenska University Hospital, Gothenburg, Sweden; f Central Hospital Växjö, Växjö, Sweden; g Department of Internal Medicine, Centralsjukhuset, Kristianstad, Sweden; h Statistiska Konsultgruppen Sweden AB, Gothenburg, Sweden

**Keywords:** Type 1 diabetes, Glycaemic control, HbA1c, Telemedicine, Digital health, Time in range

## Abstract

**Background:**

Although continuous glucose monitor/intermittent scanning continuous glucose monitor (CGM/isCGM) is widely used for glucose monitoring, many adults with type 1 diabetes (T1D) still fail to achieve recommended glycaemic targets. We aimed to evaluate whether digital distance counselling based on CGM data could improve glycaemic control in adults with T1D and suboptimal control.

**Methods:**

In this multicentre, open-label, randomised controlled trial, adults with T1D and HbA1c ≥58 mmol/mol, already using CGM/isCGM with insulin therapy (multiple daily injections or pump), were enrolled across eight sites in Sweden and Norway. Participants were allocated (1:1) via a minimisation algorithm to receive either systematic intensive therapy (SIT) or conventional therapy (CT). The SIT group received weekly distance counselling, including CGM interpretation, if mean glucose was ≥8·4 mmol/L, during an 18-week intervention period. The control group attended two clinical visits during this period. The primary outcome was change in HbA1c from baseline to 18 weeks. Adverse events of special interest (AESI; severe hypoglycaemia or diabetic ketoacidosis) were assessed in the safety population. This trial is registered at clinicaltrials.gov number NCT03474393.

**Findings:**

117 participants were enrolled and randomised (59 SIT, 58 control). At 18 weeks, mean (SD) HbA1c decreased by −10·7 (9·4) mmol/mol (−0·98% [0·86]) in the SIT group compared with −2·4 (8·4) mmol/mol (−0·22% [0·77]) in CT, resulting in an adjusted mean difference of −8·3 mmol/mol (95% CI −11·2 to −5·5), equivalent to −0·76% (95% CI −1·02 to −0·50%; *P* < 0·0001). No AESI were observed in the SIT group, compared with one event in the control group (1·7%), giving a risk difference of −1·7% (95% CI −5·1 to 1·6%).

**Interpretation:**

SIT improves glycaemic control in adults with T1D using CGM/isCGM who are not achieving recommended glycaemic targets, without evidence of safety concerns. These findings highlight the critical role of structured, individualised interventions in addressing persistent glycaemic management deficits and advancing clinical outcomes in this population.

**Funding:**

The study was supported by the Swedish state, 10.13039/100007212Region Västra Götaland, and the 10.13039/501100008546Swedish Diabetes Foundation.

**Research in context**

**Evidence before this study**

We searched PubMed up to December 2017 and conducted an updated search after trial completion, covering publications up to February 2025, using the terms “telemedicine”, “CGM”, “type 1 diabetes”, and “glycaemic control” in different combinations. Evidence from the available literature showed that CGM and isCGM improved glycaemic control, but many individuals still did not reach the recommended glycaemic targets. A lack of RCTs was noted regarding the efficacy and safety of glucose management by telemedicine for adults with type 1 diabetes.

**Added value of this study**

In this randomised controlled trial of 117 adults, it was found that systematic intensive therapy with distance counselling improved HbA1c by 10·7 mmol/mol (0·98%) compared to 2·4 mmol/mol (0·22%) for the control group after 18 weeks. Furthermore, there was significant improvement in time in range, mean glucose, and time above range. There was no increase in hypoglycaemia or ketoacidosis during systematic intensive therapy.

**Implications of all the available evidence**

This study shows that systematic intensive therapy is an effective and safe method to improve glycaemic control for adults with type 1 diabetes already using CGM/isCGM, without increasing the risk of hypoglycaemia or ketoacidosis.

## Introduction

Treatments for persons with type 1 diabetes (T1D) have changed rapidly over the past decades, with continuous glucose monitoring (CGM) and insulin pumps being used more often.^1^ The DIAMOND and GOLD trials demonstrated that CGM reduces HbA1c by 5–7 mmol/mol (0·4–0·6%), decreases hypoglycaemia, and improves well-being in adults using multiple daily insulin injections.^2^^,^^3^ Insulin pumps have similarly been shown to lower HbA1c and increase time in range.^4^ Despite these advances, many individuals with T1D also in high-income countries like Sweden where CGM is standard care, fail to achieve the recommended HbA1c targets, leaving them at risk of serious diabetes complications.^5^ This raises a critical question: How can diabetes care teams further support individuals with T1D in achieving optimal glycaemic control?

The American Diabetes Association (ADA) emphasises a person-centred approach in diabetes management, advocating for healthcare teams to facilitate individualised self-care strategies.^6^ Telehealth or telemedicine, endorsed by the ADA as complements to traditional clinical visits,^7^ have shown promise in improving glycaemic control and quality of life, as evidenced by observational studies.^8^, ^9^, ^10^ By enabling real-time interpretation of CGM data in the context of daily life, telemedicine reduces the time-burden for patients and fosters more timely, tailored interventions.

In low- and middle-income countries, where CGM adoption is growing but remains limited to a minority due to constrained healthcare resources,^11^ distance counselling offers significant potential. Digital support can bridge gaps in care by providing remote guidance, optimising the use of scarce resources, and improving logistics. However, randomised controlled trials are needed to evaluate the impact of telehealth and distance counselling on glycaemic outcomes, patient experience, and safety as integral components of T1D management.

The primary aim of this study was to evaluate whether digital support and closer collaboration with a diabetes nurse, focusing on CGM data interpretation, could improve glycaemic control in adults with T1D and suboptimal control. Additional exploratory objectives were to examine whether the intervention could alleviate diabetes-related distress and to evaluate the safety of the treatment.

## Methods

### Study design

The Systematic Intensive Therapy (SIT) trial (Trial registration NCT03474393) was an investigator-initiated, open-label, randomised controlled trial conducted across eight diabetes outpatient clinics in Sweden and Norway. Participants were randomised to receive either a 4-month systematic intensive therapy or standard care. The SIT group underwent weekly distance counselling, including CGM interpretation when mean glucose was >8·4 mmol/L, over an 18-week period. The control group received standard care with two clinical visits during that period. Both groups attended two clinical visits during the 8-month follow-up period.

Further details of the study design have been published previously.^12^ The full trial protocol and subsequent amendments are available in the Supplemental Material. Amendments included the addition of study sites, clarification of safety procedures to minimise the risk of hypoglycaemia, a sample size adjustment due to recruitment challenges during the COVID-19 pandemic, and the reordering of endpoints to enable a confirmatory hierarchical testing procedure for the primary and key secondary endpoints.

### Inclusion and exclusion criteria

Adults aged ≥18 years with type 1 diabetes, HbA1c ≥58 mmol/mol, and current use of CGM or intermittent scanning CGM (isCGM) were eligible for inclusion. Exclusion criteria included a diabetes duration of less than a year and planned or recent changes in diabetes management within the past 3 months (e.g., switching between multiple daily injections [MDI] and insulin pumps, or initiating or discontinuing CGM/isCGM use). Full details are provided in Supplemental Table S1.

### Randomisation

Participants were allocated in a 1:1 ratio to either SIT or conventional therapy using a secure, web-based minimisation algorithm managed by dSharp Consulting (Gothenburg, Sweden). The first four participants were randomised using permuted blocks. Thereafter, a minimisation algorithm was used, assigning a higher probability to the group that minimised imbalance across sex, HbA1c, route of insulin delivery (multiple daily injections or insulin pump) and sensor type (CGM or isCGM). Access to the system was restricted to authorised study personnel.

### Data management

Study data were collected and managed using the MediCase electronic data capture system (MediCase AB, Gothenburg, Sweden), which ensures secure and standardised handling of clinical trial data. Data entry was performed by trained personnel and supported by built-in validation checks and predefined quality control procedures to ensure data accuracy, completeness, and consistency.

### Treatment

All participants were instructed on how to upload and interpret their CGM data. This included evaluation of nocturnal and morning glucose levels, pre- and postprandial glucose excursions, insulin timing in relation to meals and exercise, time spent in different glycaemic ranges, and overall glycaemic variability.

Participants in the SIT group received weekly 5–15 min telephone calls to review their digitally uploaded CGM data. During these calls, diabetes nurses provided guidance on improving glucose levels, achieving individualised glucose targets, and interpreting CGM patterns. Insulin dose adjustments were discussed, and possible causes of hypoglycaemia or hyperglycaemia from the preceding week were reviewed. If a participant's mean glucose was ≤8·4 mmol/L (corresponding to HbA1c 52 mmol/mol), no contact was made that week, but data was uploaded again the following week.

### Clinical visits

Both groups attended clinical visits at diabetes outpatient clinics at randomisation and at weeks 10, 18, 32 and 52. At all clinical visits, HbA1c was measured, CGM data uploaded, insulin doses reviewed, and adverse events recorded.

### Endpoints

The primary endpoint was the change in HbA1c from baseline to week 18 (end of intervention). Secondary endpoints included the following CGM metrics: changes in time in range (TIR, 3·9–10·0 mmol/L), mean glucose, and time above range (TAR, >10·0 mmol/L) from baseline to week 18, as well as change in HbA1c from baseline to weeks 32 and 52. Exploratory endpoints comprised time below range (TBR, <3·9 mmol/L), TAR level 2 (>13·9 mmol/L), standard deviation (SD) of glucose values, coefficient of variation (CV) and mean amplitude of glycaemic excursions (MAGE). Patient-reported outcomes were assessed using the Hypoglycaemia Confidence Scale (HCS),^13^ the Diabetes Treatment Satisfaction Questionnaire,^14^ status and change versions (DTSQs and DTSQc), and the Diabetes Distress Scale (DDS).^15^ The HCS ranges from 1 to 4, with higher scores indicating greater confidence; the DTSQs and DTSQc range from 0 to 36 and −18 to +18, respectively, with higher scores reflecting greater treatment satisfaction; and the DDS ranges from 1 to 6, with higher scores indicating greater diabetes-related distress. All study endpoints and their corresponding evaluation timepoints are listed in Supplemental Table S2.

### Safety and adverse events

All serious adverse events were documented during scheduled clinical visits. Additionally, adverse events of special interest (AESI), defined as severe hypoglycaemia or diabetic ketoacidosis, were recorded throughout the study period. The trial was monitored by an external study monitor in accordance with Good Clinical Practice (GCP) to ensure participant safety and data integrity. Given the behavioural nature and low-risk profile of the intervention, no independent data monitoring committee was established.

### Sample size

The study was powered to detect a clinically meaningful 4·4 mmol/mol (0·4%) improvement in the primary outcome, change in HbA1c from baseline to 18 weeks, with SIT compared to conventional therapy. This target difference was chosen based on clinically relevant thresholds and prior CGM intervention studies in T1D. Assuming a common SD of 8·8 mmol/mol (0·8%), this corresponds to a standardised effect size of 0·5. With 1:1 allocation, 64 participants per group were required to achieve 80% power at a two-sided significance level of α = 0·05. To account for an anticipated 10% dropout rate, the planned sample size was increased to 142 participants. Due to recruitment challenges during the COVID-19 pandemic, the target sample size was subsequently revised to 120 participants, providing 80% power to detect a 4·8 mmol/mol (0·44%) difference in HbA1c under the same assumptions. No correction for clustering across sites was applied in the sample size calculation. Sample size calculations were performed using the POWER procedure in SAS/STAT® software, version 9·4 (SAS Institute Inc., Cary, NC, USA).

### Statistics

All randomised participants were included in the intention-to-treat (ITT) population. Two per-protocol (PP) populations were defined prior to database lock. PP1 included all participants who completed visits at weeks 10 and 18, provided HbA1c data at week 18, and, for those in the SIT group, additionally attended at least 70% of scheduled SIT contacts. PP2 included participants meeting PP1 criteria who also attended visits at weeks 32 and 52 and provided HbA1c data at all follow-up visits. The safety population comprised all randomised participants, analysed according to the treatment actually received, and was identical to the ITT population. Efficacy analyses were conducted in both the ITT and PP populations, and safety analyses in the safety population.

Descriptive data were summarised as mean (SD) or median (interquartile range; IQR) for continuous variables, and counts (percentages) for categorical variables. Efficacy analyses were conducted using analysis of covariance (ANCOVA) to assess changes from baseline, adjusting for baseline values. Skewed variables and questionnaire scales (TAR, TBR, DTSQs, HCS, DDS) were analysed using robust standard errors (HC3 estimator). The DTSQc, for which baseline values are not applicable, was analysed using a two-sample t-test. Binary variables were compared using the Farrington-Manning test. Adverse events were summarised using Wilson confidence intervals for within-group proportions and compared between groups using the Farrington–Manning test for absolute risk differences, with corresponding confidence intervals. Missing data were handled using multiple imputation with chained equations, incorporating baseline values, minimisation variables, and outcomes from earlier and later visits. To account for potential clustering by sites, sensitivity analyses were conducted using linear mixed-effects models with study site as a random effect. Additionally, the primary and secondary efficacy analyses were repeated stratified by minimisation variables and study site.

Correction for multiple testing was implemented using hierarchical testing of the primary and secondary endpoints in the pre-specified order (see Table 2), controlling the family-wise error rate at α = 0·05. Exploratory endpoints were analysed at the nominal 5% significance level without adjustment for multiplicity.

Changes from the protocol included replacing last observation carried forward (LOCF) with multiple imputation and substituting non-parametric tests for patient-reported outcomes (DDS, DTSQ, HCS) with parametric tests to ensure compatibility with the imputation method. All analyses were pre-specified in the statistical analysis plan (SAP) prior to database lock; the SAP is provided in the Supplement.

Statistical analyses were conducted in SAS/STAT® software, version 9·4 (SAS Institute Inc., Cary, NC, USA).

### Ethics approval

The study was approved by the Swedish Ethical Review Authority (DNR 225-18) and the Regional Committees for Medical and Health Research Ethics in Norway (ID 93133). All participants provided written informed consent prior to enrolment.

### Role of the funding source

The funders had no role in the design or conduct of the study, including collection, management, analysis, and interpretation of the data, or the preparation, review, or approval of the manuscript for publication.

## Results

### Patient characteristics

**Fig. 1:**
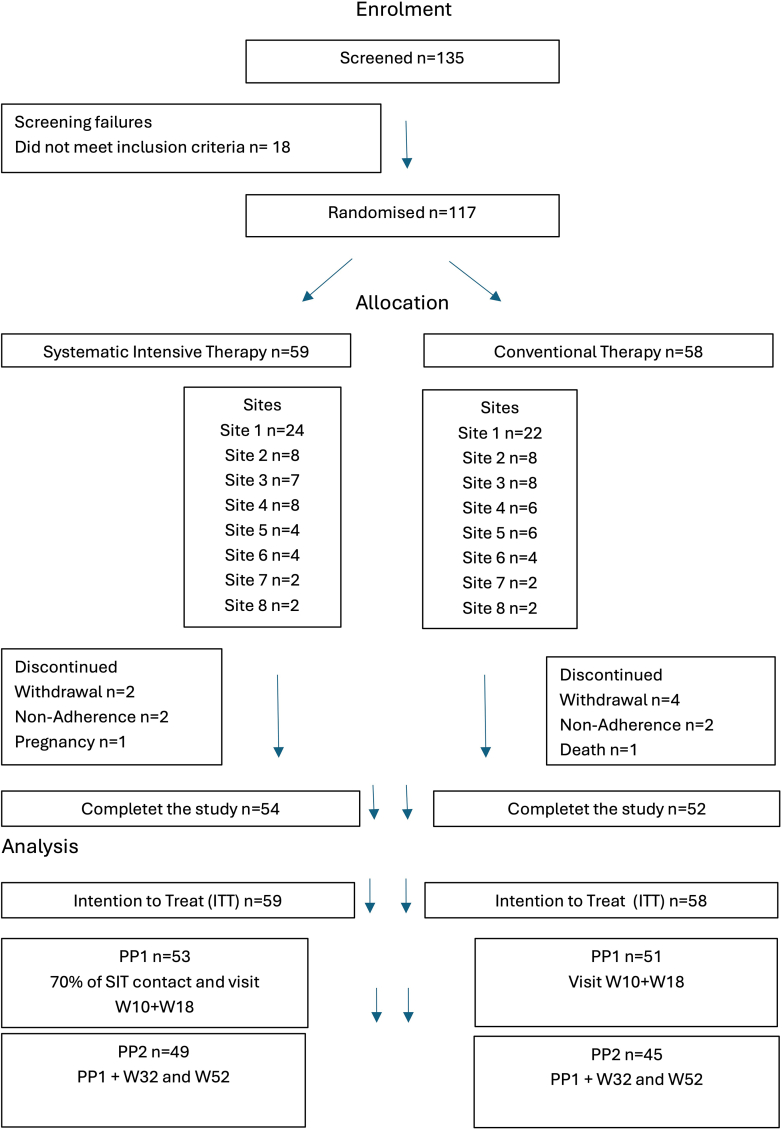
Flowchart of study participation, showing the number of participants screened, randomised, discontinued, completed, and analysed according to ITT, PP1, and PP2 populations.

Between November 2018 and April 2022, 117 participants were enrolled, with 59 allocated to the SIT group and 58 to the conventional therapy group (Fig. 1). The median (IQR) age of participants was 46 (32–57) years, 43% (n = 50) were female, and the mean (SD) HbA1c at baseline was 71 (13) mmol/mol or 8·6% (1·2), with similar characteristics across groups. Additional baseline characteristics are shown in Table 1 for the ITT population and in Supplemental Tables S3 and S4 for the PP populations. The distribution of participants across study sites is shown in Fig. 1 and Supplemental Table S5.

**Table 1:** Baseline characteristics of study participants randomised to SIT or conventional therapy (ITT population).

	ITT population (n = 117)	SIT (n = 59)	Conventional therapy (n = 58)
Age (years)	46 (32–57)	44 (33–58)	47 (32–56)
Female sex, n (%)	50 (42·7%)	25 (42·4%)	25 (43·1%)
Diabetes duration (years)	23 (14–34)	26 (14–35)	22 (14–34)
Weight (kg)	84·1 (17·0)	85·3 (17·1)	82·9 (16·9)
Height (cm)	175 (9·7)	175 (10·5)	176 (8·9)
BMI (kg/m^2^)	27·3 (4·8)	27·8 (5·1)	26·7 (4·5)
Smoking, n (%)	21 (17·9%)	9 (15·3%)	12 (20·7%)
HbA1c (mmol/mol)	70·6 (13·2)	70·5 (11·4)	70·8 (15·0)
HbA1c (%)	8·61 (1·21)	8·60 (1·04)	8·63 (1·37)
Mean glucose (mmol/L)	11·0 (2·0)	11·1 (2·1)	11·0 (2·0)
Time in range (% of time 3·9–10 mmol/L)	43·0 (15·3)	42·4 (15·3)	43·8 (15·4)
Time above range (% of time >10 mmol/L)	53·5 (16·7)	54·1 (17·0)	52·9 (16·4)
Glucose sensor type			
Medtronic 640G, n (%)	8 (6·8%)	3 (5·1%)	5 (8·6%)
Dexcom G4, n (%)	2 (1·7%)	2 (3·4%)	0 (0·0%)
Dexcom G5, n (%)	6 (5·1%)	3 (5·1%)	3 (5·2%)
Dexcom G6, n (%)	27 (23·1%)	16 (27·1%)	11 (19·0%)
FreeStyle Libre, n (%)	66 (56·4%)	32 (54·2%)	34 (58·6%)
FreeStyle Libre 2, n (%)	7 (6·0%)	3 (5·1%)	4 (6·9%)
Guardian Link, n (%)	1 (0·9%)	0 (0·0%)	1 (1·7%)
DTSQs total score^a^	26·1 (5·5)	25·5 (5·3)	26·7 (5·7)
Diabetes distress scale (DDS)^b^	2·2 (0·68)	2·2 (0·68)	2·2 (0·68)
Hypoglycaemia confidence scale (HCS)^c^	3·1 (0·60)	3·2 (0·61)	3·0 (0·58)
Insulin doses (IU/day)			
Total bolus insulin	24 (16–35)	25 (16–40)	24 (15–32)
Total basal insulin	30 (19–40)	30 (18–42)	31 (20–37)
Total daily insulin	55 (38–72)	54 (43–72)	57 (35–71)
Route of insulin delivery			
Insulin pump	39 (33·3%)	19 (32·2%)	20 (34·5%)
Multiple daily injections	78 (66·7%)	40 (67·8%)	38 (65·5%)

Descriptive data are presented as mean (SD) or median (IQR) for numeric variables, as appropriate, and as counts and percentages for categorical variables.

**Abbreviations**: BMI, body mass index; DTSQs, Diabetes Treatment Satisfaction Questionnaire, status version; HbA1c, glycated haemoglobin A1c; IQR, interquartile range; IU, international units; SD, standard deviation.

a The DTSQs ranges from 0 to 36, with higher scores reflecting greater treatment satisfaction.

b The DDS ranges from 1 to 6, with higher scores indicating greater diabetes-related distress.

c The HCS ranges from 1 to 4, with higher scores indicating greater confidence in managing hypoglycaemia.

### HbA1c changes

The primary endpoint, change in HbA1c from baseline to 18 weeks, showed a mean (SD) reduction of −10·7 (9·4) mmol/mol (0·98% [0·86]) in the SIT group (from 70·5 to 59·9 mmol/mol) compared to −2·4 (8·4) mmol/mol (0·22% [0·77]) (from 70·8 to 68·4 mmol/mol) in the conventional therapy group, with a mean difference of −8·3 mmol/mol (0·76%) (95% CI −11·2 to −5·5 mmol/mol; *P* < 0·0001; Table 2). After the intervention phase ended at week 18, and both groups only received support at clinical visits, the SIT group maintained a statistically significant improvement in HbA1c at week 32. Mean (SD) HbA1c reductions were −7·8 (9·3) mmol/mol (0·71% [0·85]) in the SIT group compared to −3·6 (9·4) mmol/mol (0·33% [0·86]) in the conventional therapy group, with a mean difference of −4·1 mmol/mol (0·38%) (95% CI −7·2 to −0·9 mmol/mol; *P* = 0·012). At 52 weeks, the difference in HbA1c between the groups remained numerically similar to that of week 32, with a mean difference of −4·1 mmol/mol (0·38%) (95% CI −8·2 to 0·1 mmol/mol), with corresponding mean (SD) reductions of −6·2 (10·5) mmol/mol (0·57% [0·96]) in the SIT group and −2·2 (12·3) mmol/mol (0·20% [1·13]) in the control group (*P* = 0·053, Table 2). Longitudinal changes in HbA1c are illustrated graphically in Supplemental Figure S1.

**Table 2:** Primary and secondary efficacy analyses of changes in glycaemic control with systematic intensive therapy (SIT) versus conventional therapy (ITT population).

	SIT (n = 59)	Conventional therapy (n = 58)	Adjusted mean difference (95% CI)	*P*
Change from baseline to 18 weeks				
HbA1c (mmol/mol)	−10·7 (9·4)	−2·4 (8·4)	−8·3 (−11·2, −5·5)	<0·0001
TIR (% of time 3·9–10 mmol/L)	11·9 (13·5)	1·2 (13·7)	10·0 (5·3, 14·9)	<0·0001
Mean glucose (mmol/L)	−1·4 (1·6)	−0·1 (1·4)	−1·3 (−1·9, −0·8)	<0·0001
TAR (% of time >10 mmol/L)	−13·2 (14·8)	−1·7 (14·7)	−11·2 (−16·4, −6·0)	<0·0001
Change from baseline to 32 weeks				
HbA1c (mmol/mol)	−7·8 (9·3)	−3·6 (9·4)	−4·1 (−7·2, −0·9)	0·012
Change from baseline to 52 weeks				
HbA1c (mmol/mol)	−6·2 (10·5)	−2·2 (12·3)	−4·1 (−8·2, 0·1)	0·053

Descriptive data are presented as means and standard deviations.

Statistical analyses were performed using analysis of covariance (ANCOVA), adjusting for baseline values. Robust (heteroscedasticity-consistent) standard errors were employed for non-normally distributed variables (TAR). Missing data were handled using multiple imputation. Results are presented as adjusted mean differences with 95% confidence intervals (CIs).

Secondary endpoints were tested hierarchically at α = 0·05, in the order listed, contingent on statistical significance of the primary endpoint.

**Abbreviations**: CI, confidence interval; HbA1c, glycated haemoglobin A1c; TAR, time above range; TIR, time in range.

**Fig. 2:**
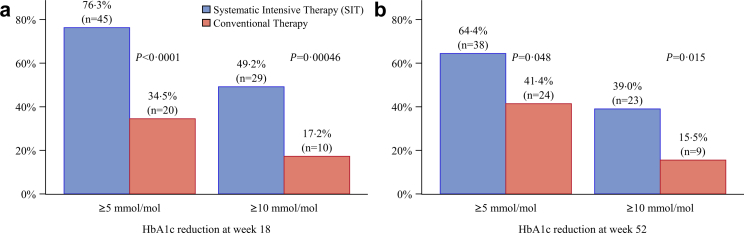
Proportion of participants achieving an HbA1c reduction of ≥5 mmol/mol or ≥10 mmol/mol at week 18 (**a**) and week 52 (**b**) with SIT compared to conventional therapy. A higher proportion of participants achieved HbA1c reductions of ≥5 mmol/mol (difference 41·8%, 95% CI 23·8–59·8%) and ≥10 mmol/mol (difference 32%, 95% CI 14·8–49·0%) with SIT compared to conventional therapy at the end of treatment. These improvements were sustained at 52 weeks, with corresponding differences of 23·0% (95% CI 4·9–41·1%) for ≥5 mmol/mol and 23·5% (95% CI 7·3–39·6%) for ≥10 mmol/mol.

At 18 weeks, 76% (45/59) of participants in the SIT group achieved an HbA1c reduction of ≥5 mmol/mol (0·46%) versus 34% (20/58) in the control group, while 49% (29) achieved a reduction of ≥10 mmol/mol (0·91%) versus 17% (10) in the control group (Fig. 2a). At week 52, 64% (38) of participants in the SIT group achieved an HbA1c reduction of ≥5 mmol/mol compared to 41% (24) in the control group. Similarly, 39% (23) of participants in the SIT group achieved an HbA1c improvement of ≥10 mmol/mol, compared to 16% (9) in the control group (Fig. 2b).

The number of participants achieving the HbA1c target of ≤53 mmol/mol (≤7%) increased from none at baseline to 10 (17%) at the end of treatment and 8 (14%) at the end of the study in the SIT group, compared to 2 (3%), and 6 (10%), respectively, in the conventional therapy group (Supplemental Table S6).

### CGM metrics

Time in range increased, with a mean difference between the groups of 10% (95% CI 5·3–14·9; *P* < 0·0001) in favour of the SIT group. Mean glucose level based on CGM data also improved in the SIT group compared with the conventional therapy at week 18, with mean changes of −1·4 mmol/L and −0·1 mmol/L, respectively, yielding a mean difference of −1·3 mmol/L (95% CI −1·9 to −0·8; *P* < 0·0001). Additionally, participants in the SIT group reduced their time above range by a mean of 13% compared to 2% with the conventional therapy, with a mean difference of −11% (95% CI −16·4 to −6·0; *P* < 0·0001) (Table 2). Additional descriptive statistics are presented in Supplemental Table S7 and graphically in Supplemental Figure S1. The number of participants achieving the recommended TIR threshold of ≥70% was comparable at baseline (2 [3%] in each group), but increased in both groups at the end of treatment (9 [15%] vs 6 [10%]) and at study end (12 [20%] vs 6 [10%]) in favour of the SIT group (Supplemental Table S6).

Further CGM-derived outcomes confirmed a beneficial and statistically significant effect of SIT over conventional therapy. Mean glucose levels were lower in the SIT group at weeks 18, 32, and 52, along with reduced time above range level 2 (>13·9 mmol/L). Time in tight range (3·9–7·8 mmol/L) increased at weeks 18 and 32, while time below range (<3·9 mmol/L) was slightly elevated at week 18. Glucose variability, measured by the SD of glucose values, was numerically reduced in the SIT group, whereas variability measured by the coefficient of variation (CV) was slightly increased at 18 weeks (Table 3).

**Table 3:** Changes in exploratory endpoints over time in the systematic intensive therapy (SIT) and conventional therapy groups (ITT population).

	SIT (n = 59)	Conventional therapy (n = 58)	Adjusted mean difference (95% CI)	*P*
Change from baseline to 18 weeks				
TBR (% of time <3·9 mmol/L)	0·54 (3·46)	−0·60 (2·60)	1·42 (0·48, 2·35)	0·0033
TITR (% of time 3·9–7·8 mmol/L)	8·54 (10·77)	1·13 (11·26)	7·75 (4·13, 11·37)	<0·0001
TBR level 2 (% of time <3·0 mmol/L)	−0·06 (2·40)	−0·54 (1·39)	0·69 (0·20, 1·19)	0·0063
TAR level 2 (% of time >13·9 mmol/L)	−10·21 (12·68)	−0·20 (11·39)	−9·30 (−13·4, −5·15)	<0·0001
SD of glucose values (mmol/L)	−0·33 (0·84)	−0·10 (0·66)	−0·19 (−0·45, 0·08)	0·16
CV of glucose values (%)	0·46 (6·46)	−0·78 (5·13)	1·92 (0·07, 3·77)	0·043
MAGE (mmol/L)	−1·02 (1·94)	−0·09 (1·58)	−0·75 (−1·36, −0·15)	0·015
DTSQs^a^	5·49 (5·05)	1·73 (4·90)	3·19 (1·51, 4·87)	0·00027
DTSQc^b^	14·60 (2·90)	9·70 (6·07)	4·93 (3·18, 6·68)	<0·0001
Hypoglycaemia confidence scale (HCS)^c^	0·21 (0·45)	0·14 (0·40)	0·12 (−0·03, 0·27)	0·11
Diabetes distress scale (DDS)^d^	−0·30 (0·50)	−0·05 (0·38)	−0·27 (−0·44, −0·10)	0·0020
Change from baseline to 32 weeks				
Mean glucose (mmol/L)	−0·75 (1·75)	0·04 (1·33)	−0·77 (−1·32, −0·22)	0·0065
TIR (% of time 3·9–10 mmol/L)	7·40 (13·93)	−0·03 (11·13)	6·94 (2·37, 11·52)	0·0033
TBR (% of time <3·9 mmol/L)	−0·29 (4·83)	−0·26 (2·70)	0·27 (−0·98, 1·52)	0·67
TAR (% of time >10 mmol/L)	−8·00 (15·91)	−0·46 (12·06)	−7·29 (−12·4, −2·20)	0·0055
TITR (% of time 3·9–7·8 mmol/L)	4·60 (11·61)	0·42 (8·87)	4·48 (0·94, 8·02)	0·014
TBR level 2 (% of time <3·0 mmol/L)	−0·37 (3·18)	−0·37 (1·79)	0·24 (−0·47, 0·96)	0·50
TAR level 2 (% of time >13·9 mmol/L)	−6·77 (13·07)	0·53 (10·85)	−6·64 (−11·0, −2·30)	0·0030
SD of glucose values (mmol/L)	−0·19 (0·79)	−0·04 (0·72)	−0·11 (−0·39, 0·17)	0·43
CV of glucose values (%)	−0·47 (5·54)	−0·10 (5·83)	0·23 (−1·65, 2·11)	0·81
MAGE (mmol/L)	−0·56 (1·90)	0·23 (1·81)	−0·63 (−1·29, 0·03)	0·061
DTSQs^a^	4·00 (5·51)	1·15 (4·95)	2·29 (0·46, 4·11)	0·014
Hypoglycaemia confidence scale (HCS)^c^	0·24 (0·47)	0·09 (0·37)	0·22 (0·08, 0·37)	0·0025
Diabetes distress scale (DDS)^d^	−0·25 (0·50)	−0·11 (0·39)	−0·16 (−0·32, −0·01)	0·033
Change from baseline to 52 weeks				
Mean glucose (mmol/L)	−0·47 (1·80)	0·18 (1·48)	−0·65 (−1·27, −0·02)	0·043
TIR (% of time 3·9–10 mmol/L)	6·00 (14·35)	0·51 (13·30)	5·15 (−0·22, 10·51)	0·060
TBR (% of time <3·9 mmol/L)	−0·65 (4·65)	−0·82 (2·60)	0·50 (−0·62, 1·62)	0·38
TAR (% of time >10 mmol/L)	−6·26 (16·62)	−0·68 (14·00)	−5·37 (−11·3, 0·54)	0·074
TITR (% of time 3·9–7·8 mmol/L)	4·24 (11·60)	0·40 (10·73)	4·08 (−0·02, 8·17)	0·051
TBR level 2 (% of time <3·0 mmol/L)	−0·62 (2·75)	−0·50 (1·58)	0·11 (−0·47, 0·68)	0·71
TAR level 2 (% of time >13·9 mmol/L)	−5·17 (13·97)	0·33 (12·27)	−5·08 (−10·1, −0·03)	0·049
SD of glucose values (mmol/L)	−0·27 (0·95)	−0·17 (0·83)	−0·05 (−0·36, 0·26)	0·75
CV of glucose values (%)	−1·20 (6·10)	−1·52 (6·31)	1·01 (−1·06, 3·08)	0·34
MAGE (mmol/L)	−0·71 (1·97)	−0·24 (1·75)	−0·30 (−0·95, 0·35)	0·37
DTSQs^a^	3·30 (5·36)	1·65 (5·17)	1·10 (−0·74, 2·93)	0·24
Hypoglycaemia confidence scale (HCS)^c^	0·24 (0·61)	0·21 (0·43)	0·13 (−0·05, 0·31)	0·16
Diabetes distress scale (DDS)^d^	−0·26 (0·48)	−0·15 (0·41)	−0·13 (−0·29, 0·03)	0·098

Descriptive data are presented as means and standard deviations.

Statistical analyses were performed using analysis of covariance (ANCOVA), adjusting for baseline values. Robust (heteroscedasticity-consistent) standard errors were employed for non-normally distributed variables (TAR, TBR, DTSQs, HCS, and DDS). Missing data were handled using multiple imputation. Results are presented as adjusted mean differences with 95% confidence intervals (CIs).

**Abbreviations**: CI, confidence interval; CV, coefficient of variation; DDS, diabetes distress scale; DTSQc, diabetes treatment satisfaction questionnaire, change version; DTSQs, diabetes treatment satisfaction questionnaire, status version; HbA1c, glycated haemoglobin A1c; HCS, hypoglycaemia confidence scale; MAGE, mean amplitude of glycaemic excursions; SD, standard deviation; TAR, time above range; TBR, time below range; TIR, time in range; TITR, time in tight range.

a The DTSQs ranges from 0 to 36, with higher scores reflecting greater treatment satisfaction.

b The DTSQc ranges from −18 to +18, with higher scores reflecting greater treatment satisfaction.

c The HCS ranges from 1 to 4, with higher scores indicating greater confidence in managing hypoglycaemia.

d The DDS ranges from 1 to 6, with higher scores indicating greater diabetes-related distress.

### Patient-reported outcomes

Patient-reported outcomes favoured SIT, with greater treatment satisfaction (DTSQs and DTSQc) and reduced diabetes-related distress (DDS) during the intervention and at week 32 but attenuated at week 52. Despite lower glucose levels, hypoglycaemia confidence was similar to the control group at week 18 and higher in the SIT group by week 32 (Table 3). Descriptive statistics for exploratory endpoints by visit are presented in Supplemental Table S7.

### Per-protocol analyses

Per-protocol analyses, conducted on populations of 104 and 94 individuals respectively, confirmed the beneficial effects of SIT on the primary endpoint compared to conventional therapy. Results for glucose metrics and patient-reported outcomes followed similar patterns to the ITT analysis, demonstrating advantages for SIT (Supplemental Tables S8 and S9).

### Safety and adverse events

Eight serious adverse events were reported during the study: five events (8·5%) in the SIT group and three events (5·2%) in the conventional therapy group (risk difference 3·3%, (95% CI −5·8 to 12·4%). No events of severe hypoglycaemia or diabetic ketoacidosis were reported in the SIT group, whereas one severe hypoglycaemia event (1·7%) was observed in the conventional therapy group, corresponding to a risk difference of −1·7% (95% CI −5·1 to 1·6%) (Table 4).

**Table 4:** Incidence of serious adverse events (SAE) and adverse events of special interest (AESI) in the systematic intensive therapy (SIT) and conventional therapy groups (safety population).

	SIT (n = 59)	95% CI (% points)	Conventional therapy (n = 58)	95% CI (% points)	Risk difference (95% CI)
Any SAE	5 (8·5%)	3·7, 18·4	3 (5·2%)	1·8, 14·1	3·3% (−5·8, 12·4)
AESI^a^	0 (0·0%)	0·0, 6·1	1 (1·7%)	0·3, 9·1	−1·7% (−5·1, 1·6)
Severe hypoglycaemia	0 (0·0%)	0·0, 6·1	1 (1·7%)	0·3, 9·1	−1·7% (−5·1, 1·6)
Diabetic ketoacidosis	0 (0·0%)	0·0, 6·1	0 (0·0%)	0·0, 6·2	0·0% (−6·2, 6·1)
Other SAEs					
Aortobifemoral bypass	1 (1·7%)	0·3, 9·0	0 (0·0%)	0·0, 6·2	1·7% (−1·6, 5·0)
Death	0 (0·0%)	0·0, 6·1	1 (1·7%)	0·3, 9·1	−1·7% (−5·1, 1·6)
Gallstone surgery	0 (0·0%)	0·0, 6·1	1 (1·7%)	0·3, 9·1	−1·7% (−5·1, 1·6)
Headache and balance problems	1 (1·7%)	0·3, 9·0	0 (0·0%)	0·0, 6·2	1·7% (−1·6, 5·0)
Pilonidal cyst/abscess	1 (1·7%)	0·3, 9·0	0 (0·0%)	0·0, 6·2	1·7% (−1·6, 5·0)
Retinal detachment requiring surgery	1 (1·7%)	0·3, 9·0	0 (0·0%)	0·0, 6·2	1·7% (−1·6, 5·0)
Urosepsis	1 (1·7%)	0·3, 9·0	0 (0·0%)	0·0, 6·2	1·7% (−1·6, 5·0)

Data are presented as counts and percentages, with 95% confidence intervals calculated using the Wilson score method.

Confidence intervals for absolute risk differences were calculated using the Farrington–Manning method.

**Abbreviations**: AE, adverse event; AESI, adverse event of special interest; CI, confidence interval; SAE, serious adverse event.

a AESI was defined as severe hypoglycaemia or diabetic ketoacidosis.

### Sensitivity analyses

In a sensitivity analysis accounting for clustering by study site, the mean difference in HbA1c change from baseline to 18 weeks was −8·2 mmol/mol (95% CI −11·0 to −5·4; *P* < 0·0001) for SIT versus conventional therapy. Time in range increased by 10·1% (5·4–14·8; *P* < 0·0001), mean glucose decreased by 1·3 mmol/L (0·8–1·8; *P* < 0·0001), and time above range decreased by 11·2% (5·6–16·8; *P* = 0·00014). At 32 weeks, HbA1c remained lower in the SIT group by −3·7 mmol/mol (95% CI −6·6 to −0·8; *P* = 0·014), and at 52 weeks by −3·8 mmol/mol (95% CI −7·6 to −0·0; *P* = 0·048) (Supplemental Table S10). Sensitivity analyses stratified by minimisation variables showed consistent improvements in primary and secondary endpoints across subgroups (Supplemental Figures S2–S7), with larger treatment effects observed in women and among participants with poorer glycaemic control (i.e., higher HbA1c) at baseline.

## Discussion

Systematic intensive therapy (SIT) with distance counselling based on CGM data significantly improved glycaemic control in adults with T1D compared to conventional therapy, while maintaining safety. After 4 months of SIT, HbA1c was reduced by 8 mmol/mol (0·8%) and time in range increased by 10% compared to conventional therapy. These improvements surpass the thresholds for clinical significance, where reductions of 3 mmol/mol in HbA1c and increases of 5% in TIR have been associated with reduced risks of diabetes-related complications.^16^^,^^17^ Participants receiving SIT also reported greater treatment satisfaction and less diabetes-related distress.

Safety is paramount when introducing new treatment concepts in clinical practice, particularly for remote therapies where patients are not seen and for persons with T1D, given the associated risks of acute complications such as severe hypoglycaemia and diabetic ketoacidosis (DKA). While reductions in mean glucose levels, as achieved with SIT, often increase the risk of hypoglycaemia, no cases of severe hypoglycaemia or DKA occurred during the SIT intervention.

Diabetes distress is common among individuals with T1D, and the burden of managing the condition can lead to diabetes burnout for both patients but also for caregivers of minors.^18^, ^19^, ^20^ Distance counselling offers a practical solution by facilitating everyday diabetes management. There are several advantages, including time savings and minimising disruptions to daily life and work through reduced travel. Moreover, the more frequent and closer contact enabled by remote counselling, integrated into patient's daily routines, enhances the precision of guidance. This approach simplifies discussions about glucose patterns and fosters a better understanding of their causes. These factors likely contribute to the observed improvements in glucose control, treatment satisfaction, and reduced diabetes distress with SIT.

The observed effects of SIT, with a between-group HbA1c reduction of 8 mmol/mol and a 10% increase in TIR at the end of active treatment, are substantial when compared to outcomes from other treatments evaluated in T1D. For instance, the introduction of novel insulin formulations has generally been associated with reductions in HbA1c ranging from 1 to 2 mmol/mol.^21^^,^^22^ Similarly, clinical trials comparing CGM with capillary blood glucose testing have reported reductions in HbA1c of 5–7 mmol/mol.^2^^,^^3^ Although substantial improvements in glycaemic control were achieved with SIT, participants entered the trial with severely elevated HbA1c levels, and consequently, only a minority reached the recommended glycaemic targets.^23^

SIT has the potential to significantly impact diabetes care not only in high-income countries but also in low- and middle-income countries where T1D care remains limited and hybrid closed-loop (HCL) systems are not yet widely accessible. In many regions, including Asia, Africa, South America, and Eastern Europe, access to advanced diabetes technologies such as insulin pumps and CGM is limited, though the availability of CGM is gradually increasing.^11^ With broader access to CGM, this type of support could be adapted for use across diverse healthcare systems, and possibly also across country borders. However, further evaluation is needed to determine its feasibility, scalability, and long-term impact in resource-limited settings, including formal cost-effectiveness analyses. In high-income settings, where many individuals with T1D are already using HCL systems that significantly improve glycaemic outcomes,^24^^,^^25^ SIT could still play an important role. It may serve as an adjunct for individuals who require additional support to reach glycaemic targets or who face other challenges in managing their diabetes.

Many treatments lose their efficacy once they are discontinued; for instance, the benefits of CGM are diminshed once individuals stop using their device.^2^ In the current study, as anticipated, the treatment effect attenuated when telephone contacts ceased. Nevertheless, a clinically meaningful effect on HbA1c, with a reduction of 4 mmol/mol, and an increase in time in range (TIR) of 7% persisted in the SIT group compared to controls more than 3 months post-intervention. Mean glucose estimated by CGM remained lower for the SIT group throughout the study period over 52 weeks. HbA1c showed numerically similar and clinically important reductions of 4 mmol/mol at 3 and 8 months post-intervention, with a statistically significant improvement at three months (95% CI −7·2 to −0·9; *P* = 0·012) and indications of a sustained effect at eight months (95% CI −8·2 to 0·1; *P* = 0·053), which became statistically significant in a sensitivity analysis accounting for clustering by study site (95% CI −7·6 to −0·0; *P* = 0·048).

These findings raise the essential question of how often digital counselling should be conducted and how best to phase it out. It is likely that this process needs to be individualised based on glucose levels over time and with appropriate fading procedures.^26^ In this context, artificial intelligence may play a complementary role by helping to identify patients' ongoing needs and providing adaptive support, thereby contributing to the maintenance of long-term outcomes.

The strengths of this study include its randomised design, independence from diabetes device manufacturers, and adherence to good clinical practice by independent monitoring and predefined endpoints established before enrolment. Additionally, the post-intervention phase allowed evaluation of both safety and the sustainability of effects. However, several limitations should be acknowledged. The sample size was relatively small, which limits the precision of estimates and the ability to detect rare adverse events. As a result, the study was not powered to comprehensively assess safety. The trial population was restricted to adults with T1D and an HbA1c of 58 mmol/mol (7·5%) or higher at inclusion, ethnicity was not recorded, and some of the glucose sensors used have since been replaced by newer devices. HCL pump systems were not included, as the study was largely conducted before they became widely available. Despite these limitations, the findings remain broadly applicable, as most adults with T1D, even in high-income countries, use multiple daily insulin injections. While many participants in this study were insulin pump users, the high treatment satisfaction and favourable safety profile observed with dose recommendations and adjustments suggest that remote counselling may also be beneficial for individuals using more advanced insulin pump systems.

In conclusion, this randomised trial provides evidence that systematic intensive therapy with distance counselling based on CGM data significantly improves glycaemic control in adults with T1D, with no evidence of harm. The intervention was also associated with greater treatment satisfaction and reduced diabetes-related distress, underscoring its potential value in clinical care. The approach offers more direct and frequent feedback for everyday diabetes management, benefitting those who may find it challenging or stressful to attend in-person clinical visits. Additionally, it holds significant potential in settings with limited healthcare resources, addressing barriers to care and supporting equitable access to effective diabetes management.

## Contributors

AFÓ and ML designed the trial. KAS, MW, SH, PHN, HH, and ML were principal investigators on trial sites. AFÓ and ME were diabetes nurses at sites and study coordinators. SSI was study administrator and site personnel. HI was responsible for the statistical analyses. AFÓ, HI, and ML had full access to raw data. AFÓ, SSI, ME, HI, and ML verified the data. AFÓ drafted the manuscript. ML had the overall responsibility for the trial and the final decision to submit the manuscript. All authors have critically reviewed and approved the final manuscript.

## Data sharing statement

Data from this study can be made available from the corresponding author upon reasonable request.

## Declaration of interests

AFÓ has received lecture fees from Nordic Infucare, MW has served on advisory boards and/or lectured for Astra Zeneca. SH has lectured for Novo Nordisk. PHN has shares in Novonordisk and acted as chair on local diabetes group. ML has received grants from Eli Lilly and Novonordisk and received honoraria or been consultant for Boehringer Ingelheim, Eli Lilly, Nordic Infucare, Novonordisk, and Rubin Medical. KAS, SSI, HH, ME, and HI have nothing to declare.

## Appendix A Supplementary data

Supplemental Tables and Figures

Protocol SIT

All Protocol Amendments

SIT SAP

SIT SAP Signed

SIT SAP Amendment
