# Supplementary material for: Systematic intensive therapy in addition to continuous glucose monitoring in adults with type 1 diabetes: a multicentre, open-label, randomised controlled trial
Source: Lancet Reg Health Eur. 2025 Oct 16;59:101485. doi: 10.1016/j.lanepe.2025.101485 (PMC12553072; doi:10.1016/j.lanepe.2025.101485)
Supplement: Supplemental Tables and Figures [file mmc1.pdf]

This supplement has been provided by the authors to give readers additional information about their work. Supplement to

Systematic Intensive Therapy in addition to Continuous Glucose Monitoring in Adults with Type 1 Diabetes: A Multicentre, Open-Label, Randomised Controlled Trial

Ólafsdóttir AF[1,2,5], Sveen KA[3], Wijkman M[4], Hallström S[2,5], Nilsson P-H[6], Sterner Isaksson S[1,2], Holmer H[7], Ekström M[1], Imberg H[2,8,] Lind M[1,2,5]

Affiliations

1. Department of Medicine, NU Hospital Group, Uddevalla, Sweden
2. Department of Molecular and Clinical Medicine, Institute of Medicine, Sahlgrenska Academy, University of Gothenburg, Gothenburg, Sweden
3. Department of Endocrinology, Morbid Obesity and Preventive Medicine, Oslo University Hospital, Oslo, Norway
4. Department of Health, Medicine and Caring Sciences and Department of Internal Medicine, Linköping University, Norrköping, Sweden
5. Department of Internal Medicine, Sahlgrenska University Hospital, Gothenburg, Sweden
6. Central Hospital Växjö, Växjö, Sweden
7. Department of Internal medicine, Centralsjukhuset, Kristianstad, Sweden
8. Statistiska Konsultgruppen Sweden AB, Gothenburg, Sweden

Contents

Supplemental Table S1. Inclusion and exclusion criteria. 3

Supplemental Table S2. Study endpoints.. 4

Supplemental Table S3. Baseline characteristics of study participants randomised to systematic intensive therapy (SIT) or conventional therapy (PP1 population). 5

Supplemental Table S4. Baseline characteristics of study participants randomised to systematic intensive therapy (SIT) or conventional therapy (PP2 population). 6

Supplemental Table S5. Distribution of study participants by site (ITT population). 7

Supplemental Table S6. Proportion of participants achieving HbA1c ≤53 mmol/mol and TIR ≥70% at each study visit during systematic intensive therapy (SIT) and conventional therapy (ITT population). 8

Supplemental Table S7. Descriptive statistics for primary, secondary and exploratory efficacy variables by visit (ITT population).. 9

Supplemental Table S8. Longitudinal changes in glycaemic and patient-reported outcomes in adults with type 1 diabetes undergoing 18 weeks of systematic intensive therapy (SIT) versus conventional therapy (PP1 population).. 10

Supplemental Table S9. Longitudinal changes in glycaemic and patient-reported outcomes in adults with type 1 diabetes undergoing 18 weeks of systematic intensive therapy (SIT) versus conventional therapy (PP2 population).. 12

Supplemental Table S10. Sensitivity analysis of primary and secondary endpoints using linear mixed effects models, accounting for clustering by study site (ITT population)... 14

Supplemental Figure S1. Longitudinal changes in HbA1c (a), time in range (TIR; b), mean glucose (c), and time above range (TAR; d) with SIT compared to conventional therapy. 15

Supplemental Figure S2. Subgroup analyses of the change in HbA1c from baseline to 18 weeks across minimisation variables (a) and study site (b) in the ITT population. 16

Supplemental Figure S3. Subgroup analyses of the change in time in range (TIR) from baseline to 18 weeks across minimisation variables (a) and study site (b) in the ITT population. 17

Supplemental Figure S4. Subgroup analyses of the change in mean glucose from baseline to 18 weeks across minimisation variables (a) and study site (b) in the ITT population. 18

Supplemental Figure S5. Subgroup analyses of the change in time above range (TAR) from baseline to 18 weeks across minimisation variables (a) and study site (b) in the ITT population. 19

Supplemental Figure S6. Subgroup analyses of the change in HbA1c from baseline to 32 weeks across minimisation variables (a) and study site (b) in the ITT population. 20

Supplemental Figure S7. Subgroup analyses of the change in HbA1c from baseline to 52 weeks across minimisation variables (a) and study site (b) in the ITT population. 21

Supplemental Table S1. Inclusion and exclusion criteria.

Category	Criteria
Inclusion criteria	Signed informed consent obtained before any trial-related activities.<br>Clinical diagnosis of type 1 diabetes.<br>Age ≥18 years.<br>HbA1c ≥ 58 mmol/mol at screening.<br>Current use of continuous glucose monitoring (CGM) or intermittently scanned CGM (isCGM).<br>Ability and willingness to download and share CGM/isCGM data.
Exclusion criteria	Diagnosis of Type 2 diabetes or any other form of diabetes.<br>Diabetes duration of less than one year.<br>Use of long-term systemic glucocorticoids within the past three months.<br>Recent (within the last three months) initiation, discontinuation, or modification of multiple daily injections (MDI) vs. insulin pump therapy, or CGM/isCGM use.<br>Current pregnancy, planned pregnancy, or breastfeeding within the next 12 months.<br>Planned relocation within the next 12 months that would prevent participation in study activities.<br>Any other medical, psychological, or logistical reason deemed by the investigator to make participation inappropriate.

Abbreviations: CGM, continuous glucose monitoring; HbA1c, glycated haemoglobin A1c; isCGM, intermittently scanned continuous glucose monitoring; MDI, multiple daily injections.

Supplemental Table S2. Study endpoints.

Category	Endpoint
Primary endpoint	Change in HbA1c from baseline to 18 weeks.
Secondary endpoints*	Change in TIR from baseline to 18 weeks.<br>Change in mean glucose levels from baseline to 18 weeks.<br>Change in TAR from baseline to 18 weeks.<br>Change in HbA1c from baseline to 32 weeks.<br>Change in HbA1c from baseline to 52 weeks.
Exploratory endpoints	Change in TIR from baseline to 32 and 52 weeks.<br>Change in mean glucose levels from baseline to 32 and 52 weeks.<br>Change in TAR from baseline to 32 and 52 weeks.<br>Change in TBR from baseline to 18, 32, and 52 weeks.<br>Change in TITR from baseline to 18, 32, and 52 weeks.<br>Change in TBR level 2 from baseline to 18, 32 and 52 weeks.<br>Change in TAR level 2 from baseline to 18, 32 and 52 weeks.<br>Change in SD of glucose values from baseline to 18, 32 and 52 weeks.<br>Change in CV of glucose values from baseline to 18, 32 and 52 weeks.<br>Change in MAGE from baseline to 18, 32 and 52 weeks.<br>Change in DTSQs from baseline to 18, 32 and 52 weeks.<br>DTSQc at 18 weeks.<br>Change in HCS from baseline to 18, 32 and 52 weeks.<br>Change in DDS from baseline to 18, 32 and 52 weeks.

* Secondary endpoints were tested hierarchically at $\alpha = 0{\cdot}05$, in the order listed, contingent on statistical significance of the primary endpoint.

Abbreviations: CV, coefficient of variation; DDS, diabetes distress scale; DTSQc, diabetes treatment satisfaction questionnaire, change version; DTSQs, diabetes treatment satisfaction questionnaire, status version; HbA1c, glycated haemoglobin A1c; HCS, hypoglycaemia confidence scale; MAGE, mean amplitude of glycaemic excursions; SD, standard deviation; TAR, time above range >10·0 mmol/L; TAR level 2, time above range >13·9 mmol/L; TBR, time below range <3·9 mmol/L; TBR level 2, time below range <3·0 mmol/L; TIR, time in range 3·9–10·0 mmol/L; TITR, time in tight range 3·9–7·8 mmol/L.

Supplemental Table S3. Baseline characteristics of study participants randomised to systematic intensive therapy (SIT) or conventional therapy (PP1 population).

	SIT (n=53)	Conventional Therapy (n=51)
Age (years)	47 (34–58)	47 (32–56)
Female sex, n (%)	21 (39·6%)	21 (41·2%)
Diabetes duration (years)	27 (14–36)	22 (14–34)
Weight (kg)	86·5 (17·0)	84·3 (17·1)
BMI (kg/m^2)	28·1 (5·2)	27·2 (4·5)
Smoking, n (%)	8 (15·1%)	11 (21·6%)
HbA1c (mmol/mol)	69·7 (11·0)	70·4 (13·8)
HbA1c (%)	8·5 (1·0)	8·6 (1·3)
Mean glucose (mmol/L)	10·8 (1·8)	10·8 (1·8)
Time in range (% of time 3·9–10 mmol/L)	43·9 (14·5)	45·7 (14·7)
Time above range (% of time >10 mmol/L)	52·6 (16·4)	51·2 (15·6)
DTSQs total score	25·7 (5·5)	26·7 (5·7)
Diabetes distress scale	2·13 (0·69)	2·24 (0·68)
Hypoglycaemia confidence scale	3·25 (0·63)	3·06 (0·58)
Insulin doses (IU/day)		
Total bolus insulin	24 (16–35)	24 (16–39)
Total basal insulin	30 (19–40)	29 (18–42)
Total daily insulin	55 (41–70)	54 (43–69)
Glucose sensor type, n (%)		
CGM	22 (41·5%)	18 (35·3%)
isCGM	31 (58·5%)	33 (64·7%)
Route of insulin delivery, n (%)		
Insulin pump	19 (35·8%)	17 (33·3%)
Multiple daily injections	34 (64·2%)	34 (66·7%)

Descriptive data are presented as mean (SD) or median (IQR) for numeric variables, as appropriate, and as counts and percentages for categorical variables.

PP1 included participants who attended at least 70% of scheduled SIT contacts, completed visits in weeks 10 and 18, and provided HbA1c data at week 18.

Abbreviations: BMI, body mass index; DTSQs, diabetes treatment satisfaction questionnaire, status version; HbA1c, glycated haemoglobin A1c; IQR, interquartile range; SD, standard deviation.

Supplemental Table S4. Baseline characteristics of study participants randomised to systematic intensive therapy (SIT) or conventional therapy (PP2 population).

	SIT (n=49)	Conventional Therapy (n=45)
Age (years)	50 (35–58)	47 (33–55)
Female sex, n (%)	19 (38·8%)	19 (42·2%)
Diabetes duration (years)	26 (14–37)	22 (14–34)
Weight (kg)	86·8 (17·3)	83·7 (17·4)
BMI (kg/m^2)	28·2 (5·3)	27·3 (4·6)
Smoking, n (%)	8 (16·3%)	8 (17·8%)
HbA1c (mmol/mol)	70·1 (11·4)	69·6 (12·1)
HbA1c (%)	8·6 (1·0)	8·5 (1·1)
Mean glucose (mmol/L)	10·8 (1·9)	10·7 (1·7)
Time in range (% of time 3·9–10 mmol/L)	43·5 (15·1)	46·2 (14·3)
Time above range (% of time >10 mmol/L)	53·1 (17·0)	50·6 (15·1)
DTSQs total score	25·3 (5·5)	27·1 (5·8)
Diabetes distress scale	2·15 (0·69)	2·19 (0·68)
Hypoglycaemia confidence scale	3·20 (0·63)	3·12 (0·52)
Insulin doses (IU/day)		
Total bolus insulin	27 (16–40)	23 (16–33)
Total basal insulin	29 (18–47)	31 (21–38)
Total daily insulin	54 (43–69)	56 (36–72)
Glucose sensor type, n (%)		
CGM	19 (38·8%)	16 (35·6%)
isCGM	30 (61·2%)	29 (64·4%)
Route of insulin delivery, n (%)		
Insulin pump	16 (32·7%)	15 (33·3%)
Multiple daily injections	33 (67·3%)	30 (66·7%)

Descriptive data are presented as mean (SD) or median (IQR) for numeric variables, as appropriate, and as counts and percentages for categorical variables.
PP2 included participants meeting PP1 criteria who also attended visits at weeks 32 and 52 and provided HbA1c data at all follow-ups.
Abbreviations: BMI, body mass index; DTSQs, diabetes treatment satisfaction questionnaire, status version; HbA1c, glycated haemoglobin A1c; IQR, interquartile range; SD, standard deviation.

Supplemental Table S5. Distribution of study participants by site (ITT population).

Site	SIT (n=59)	Conventional Therapy (n=58)
Uddevalla	24 (40·7%)	22 (37·9%)
Växjö	2 (3·4%)	2 (3·4%)
Norrköping	4 (6·8%)	4 (6·9%)
Göteborg Östra	8 (13·6%)	6 (10·3%)
Kristianstad	4 (6·8%)	6 (10·3%)
Kiruna	7 (11·9%)	8 (13·8%)
Skövde	2 (3·4%)	2 (3·4%)
Oslo	8 (13·6%)	8 (13·8%)

Descriptive data are presented as counts and percentages by randomisation group.

Supplemental Table S6. Proportion of participants achieving HbA1c ≤53 mmol/mol and TIR ≥70% at each study visit during systematic intensive therapy (SIT) and conventional therapy (ITT population).

Outcome	Week	SIT (n=59)	Conventional Therapy (n=58)
HbA1c ≤53 mmol/mol	0	0 (0·0%)	0 (0·0%)
	10	3 (5·1%)	2 (3·4%)
	18	10 (16·9%)	2 (3·4%)
	32	5 (8·5%)	2 (3·4%)
	52	8 (13·6%)	6 (10·3%)
TIR ≥70%	0	2 (3·4%)	2 (3·4%)
	10	10 (16·9%)	6 (10·3%)
	18	9 (15·3%)	6 (10·3%)
	32	8 (13·6%)	4 (6·9%)
	52	12 (20·3%)	6 (10·3%)

Values are number of participants (%) achieving the stated threshold at each follow-up visit.

Abbreviations: HbA1c, glycated haemoglobin A1c; SIT, systematic intensive therapy; TIR, time in range (3·9–10 mmol/L).

Supplemental Table S7. Descriptive statistics for primary, secondary and exploratory efficacy variables by visit (ITT population).

	SIT (n=59)				Conventional Therapy (n=58)			
	Baseline	18 weeks	32 weeks	52 weeks	Baseline	18 weeks	32 weeks	52 weeks
HbA1c (mmol/mol)	70·58 (11·44)	59·89 (7·38)	63·00 (10·24)	64·33 (11·91)	70·78 (14·98)	68·37 (14·52)	67·19 (13·50)	68·54 (16·20)
TIR (% of time 3·9–10 mmol/L)	42·68 (15·12)	54·53 (13·28)	50·07 (14·67)	48·67 (16·89)	44·18 (15·65)	45·42 (17·88)	44·15 (16·53)	44·69 (18·95)
Mean glucose (mmol/L)	11·05 (2·00)	9·68 (1·45)	10·30 (1·73)	10·58 (2·14)	10·97 (2·02)	10·92 (2·13)	11·02 (2·17)	11·15 (2·58)
TAR (% of time >10 mmol/L)	53·95 (16·92)	40·80 (14·11)	45·95 (15·91)	47·69 (18·12)	53·22 (16·54)	51·56 (18·60)	52·76 (17·54)	52·54 (19·68)
TBR (% of time <3·9 mmol/L)	3·81 (4·17)	4·35 (3·60)	3·52 (4·60)	3·16 (4·23)	3·23 (3·66)	2·63 (2·65)	2·97 (2·90)	2·41 (2·46)
TITR (% of time 3·9–7·8 mmol/L)	25·31 (11·18)	33·85 (9·73)	29·91 (10·83)	29·55 (12·40)	24·62 (11·29)	25·75 (12·18)	25·05 (11·33)	25·02 (12·92)
TBR level 2 (% of time <3·0 mmol/L)	1·42 (2·55)	1·35 (1·96)	1·05 (2·87)	0·79 (2·39)	1·07 (2·00)	0·53 (0·96)	0·70 (1·00)	0·57 (0·89)
TAR level 2 (% of time >13·9 mmol/L)	25·74 (16·01)	15·53 (12·30)	18·97 (12·55)	20·57 (15·92)	23·66 (15·86)	23·46 (17·26)	24·19 (17·81)	23·99 (19·98)
SD of glucose values (mmol/L)	4·19 (1·04)	3·87 (0·84)	4·01 (0·97)	3·93 (0·95)	4·07 (0·89)	3·98 (1·00)	4·03 (0·97)	3·90 (1·03)
CV of glucose values (%)	39·23 (8·03)	39·69 (5·62)	38·76 (6·09)	38·03 (6·20)	37·71 (7·09)	36·93 (7·01)	37·61 (7·19)	36·19 (7·03)
MAGE (mmol/L)	10·20 (2·34)	9·18 (1·78)	9·64 (2·18)	9·49 (1·94)	9·74 (2·20)	9·65 (2·30)	9·96 (2·34)	9·50 (2·40)
DTSQs*	25·50 (5·33)	31·00 (4·31)	29·50 (4·74)	28·81 (5·30)	26·71 (5·65)	28·44 (5·85)	27·86 (6·13)	28·36 (5·67)
DTSQc†		14·6 (2·9)				9·70 (6·07)		
HCS‡	3·21 (0·61)	3·42 (0·48)	3·45 (0·44)	3·45 (0·49)	3·03 (0·58)	3·18 (0·61)	3·12 (0·51)	3·25 (0·51)
Diabetes distress scale (DDS)§	2·15 (0·67)	1·85 (0·60)	1·90 (0·53)	1·89 (0·57)	2·24 (0·68)	2·19 (0·74)	2·13 (0·66)	2·09 (0·66)
Missing data, n (%)								
HbA1c	0 (0·0%)	6 (10·2%)	6 (10·2%)	5 (8·5%)	0 (0·0%)	4 (6·9%)	4 (6·9%)	7 (12·1%)
CGM	4 (6·8%)	5 (8·5%)	8 (13·6%)	8 (13·6%)	6 (10·3%)	4 (6·9%)	7 (12·1%)	9 (15·5%)
Questionnaires	1 (1·7%)	5 (8·5%)	8 (13·6%)	5 (8·5%)	1 (1·7%)	4 (6·9%)	6 (10·3%)	6 (10·3%)

Data are presented as estimated marginal means and standard deviations, adjusted for baseline values.
Missing data are presented as the number of participants and the percentage within each group.
*The DTSQs ranges from 0 to 36, with higher scores reflecting greater treatment satisfaction.
†The DTSQc ranges from −18 to +18, with higher scores reflecting greater treatment satisfaction.
‡The HCS ranges from 1 to 4, with higher scores indicating greater confidence in managing hypoglycaemia.
§The DDS ranges from 1 to 6, with higher scores indicating greater diabetes-related distress.
Abbreviations: CGM, continuous glucose monitoring; CV, coefficient of variation; DDS, diabetes distress scale; DTSQc, diabetes treatment satisfaction questionnaire, change version; DTSQs, diabetes treatment satisfaction questionnaire, status version; HbA1c, glycated haemoglobin A1c; HCS, hypoglycaemia confidence scale; MAGE, mean amplitude of glycaemic excursions; SD, standard deviation; TAR, time above range; TBR, time below range; TIR, time in range; TITR, time in tight range.

Supplemental Table S8. Longitudinal changes in glycaemic and patient-reported outcomes in adults with type 1 diabetes undergoing 18 weeks of systematic intensive therapy (SIT) versus conventional therapy (PP1 population).

	SIT (n=53)	Conventional Therapy (n=51)	Adjusted mean difference (95% CI)	*P*
Change from Baseline to 18 weeks				
HbA1c (mmol/mol)	−10·30 (9·41)	−3·27 (7·24)	−7·30 (−9·89, −4·71)	<0·0001
TIR (% of time 3·9–10 mmol/L)	11·96 (13·60)	1·10 (14·04)	10·02 (5·23, 14·81)	<0·0001
Mean glucose (mmol/L)	−1·26 (1·63)	0·02 (1·51)	−1·27 (−1·80, −0·74)	<0·0001
TAR (% of time >10 mmol/L)	−12·92 (14·77)	−0·81 (14·75)	−11·5 (−16·6, −6·35)	<0·0001
TBR (% of time <3·9 mmol/L)	0·52 (3·47)	−0·73 (2·75)	1·44 (0·44, 2·44)	0·0051
TITR (% of time 3·9–7·8 mmol/L)	8·47 (11·12)	0·95 (11·57)	7·70 (4·04, 11·36)	<0·0001
TBR level 2 (% of time <3·0 mmol/L)	−0·11 (2·48)	−0·60 (1·50)	0·70 (0·16, 1·23)	0·011
TAR level 2 (% of time >13·9 mmol/L)	−9·64 (12·28)	0·32 (11·31)	−9·27 (−13·3, −5·18)	<0·0001
SD of glucose values (mmol/L)	−0·30 (0·84)	−0·02 (0·55)	−0·27 (−0·53, −0·02)	0·038
CV of glucose values (%)	0·52 (6·55)	−0·77 (5·04)	1·66 (−0·22, 3·54)	0·083
MAGE (mmol/L)	−0·85 (1·93)	0·04 (1·48)	−0·81 (−1·42, −0·20)	0·010
DTSQs*	5·47 (5·12)	1·73 (5·01)	3·25 (1·49, 5·02)	0·00041
DTSQc†	14·60 (2·90)	9·70 (6·07)	4·93 (3·18, 6·68)	<0·0001
Hypoglycaemia confidence scale (HCS)‡	0·19 (0·45)	0·15 (0·42)	0·12 (−0·03, 0·27)	0·13
Diabetes distress scale (DDS)§	−0·30 (0·51)	−0·05 (0·39)	−0·28 (−0·45, −0·10)	0·0023
Change from Baseline to 32 weeks				
HbA1c (mmol/mol)	−7·46 (9·21)	−4·20 (8·69)	−3·53 (−6·45, −0·60)	0·019
TIR (% of time 3·9–10 mmol/L)	7·28 (14·34)	−0·12 (11·32)	6·70 (1·96, 11·44)	0·0060
Mean glucose (mmol/L)	−0·66 (1·79)	0·06 (1·38)	−0·70 (−1·26, −0·14)	0·014
TAR (% of time >10 mmol/L)	−7·36 (16·13)	0·26 (11·85)	−7·06 (−12·2, −1·89)	0·0080
TBR (% of time <3·9 mmol/L)	−0·30 (4·99)	−0·46 (2·81)	0·37 (−0·97, 1·72)	0·058
TITR (% of time 3·9–7·8 mmol/L)	4·43 (12·10)	0·34 (9·01)	4·24 (0·56, 7·92)	0·024
TBR level 2 (% of time <3·0 mmol/L)	−0·38 (3·36)	−0·44 (1·92)	0·29 (−0·51, 1·09)	0·48
TAR level 2 (% of time >13·9 mmol/L)	−5·78 (13·05)	0·48 (10·84)	−5·60 (−9·81, −1·39)	0·010
SD of glucose values (mmol/L)	−0·16 (0·78)	0·03 (0·63)	−0·18 (−0·45, 0·08)	0·17
CV of glucose values (%)	−0·42 (5·51)	−0·10 (5·67)	−0·00 (−1·94, 1·93)	1·00
MAGE (mmol/L)	−0·40 (1·89)	0·34 (1·80)	−0·67 (−1·38, 0·03)	0·062
DTSQs*	3·95 (5·58)	1·16 (5·10)	2·30 (0·40, 4·20)	0·018
Hypoglycaemia confidence scale (HCS)‡	0·22 (0·48)	0·08 (0·38)	0·23 (0·08, 0·38)	0·0035
Diabetes distress scale (DDS)§	−0·23 (0·51)	−0·11 (0·38)	−0·16 (−0·32, 0·00)	0·053
Change from Baseline to 52 weeks				
HbA1c (mmol/mol)	−6·14 (10·40)	−3·14 (11·41)	−3·25 (−7·14, 0·65)	0·10
TIR (% of time 3·9–10 mmol/L)	6·24 (14·63)	0·71 (13·54)	4·98 (−0·45, 10·41)	0·072
Mean glucose (mmol/L)	−0·46 (1·83)	0·04 (1·41)	−0·49 (−1·12, 0·13)	0·12
TAR (% of time >10 mmol/L)	−5·99 (16·67)	−0·38 (13·98)	−5·14 (−11·1, 0·78)	0·088
TBR (% of time <3·9 mmol/L)	−0·63 (4·81)	−0·98 (2·70)	0·58 (−0·64, 1·80)	0·35
TITR (% of time 3·9–7·8 mmol/L)	4·16 (11·89)	0·40 (10·97)	3·89 (−0·34, 8·13)	0·071
TBR level 2 (% of time <3·0 mmol/L)	−0·65 (2·91)	−0·56 (1·69)	0·13 (−0·51, 0·77)	0·69
TAR level 2 (% of time >13·9 mmol/L)	−4·57 (13·51)	−0·34 (11·78)	−3·75 (−8·63, 1·13)	0·13
SD of glucose values (mmol/L)	−0·25 (0·95)	−0·13 (0·71)	−0·12 (−0·43, 0·20)	0·46

	SIT (n=53)	Conventional Therapy (n=51)	Adjusted mean difference (95% CI)	*P*
CV of glucose values (%)	−1·10 (6·10)	−1·75 (5·90)	1·03 (−0·96, 3·01)	0·31
MAGE (mmol/L)	−0·59 (1·88)	−0·16 (1·63)	−0·36 (−1·02, 0·31)	0·29
DTSQs*	3·42 (5·16)	1·53 (5·29)	1·41 (−0·45, 3·27)	0·14
Hypoglycaemia confidence scale (HCS)‡	0·23 (0·62)	0·19 (0·42)	0·15 (−0·04, 0·34)	0·12
Diabetes distress scale (DDS)§	−0·26 (0·49)	−0·15 (0·42)	−0·15 (−0·31, 0·02)	0·075

Descriptive data are presented as means and standard deviations.
Statistical analyses were performed using analysis of covariance (ANCOVA), adjusting for baseline values. Robust (heteroscedasticity-consistent) standard errors were employed for non-normally distributed variables (TAR, TBR, DTSQs, HCS, and DDS). Missing data was handled using multiple imputation. Results are presented as adjusted mean differences with 95% confidence intervals (CIs).
PP1 included participants who attended at least 70% of scheduled SIT contacts, completed visits in weeks 10 and 18, and provided HbA1c data at week 18.
*The DTSQs ranges from 0 to 36, with higher scores reflecting greater treatment satisfaction.
†The DTSQc ranges from −18 to +18, with higher scores reflecting greater treatment satisfaction.
‡The HCS ranges from 1 to 4, with higher scores indicating greater confidence in managing hypoglycaemia.
§The DDS ranges from 1 to 6, with higher scores indicating greater diabetes-related distress.
Abbreviations: CI, confidence interval; CV, coefficient of variation; DDS, diabetes distress scale; DTSQc, diabetes treatment satisfaction questionnaire, change version; DTSQs, diabetes treatment satisfaction questionnaire, status version; HbA1c, glycated haemoglobin A1c; HCS, hypoglycaemia confidence scale; MAGE, mean amplitude of glycaemic excursions; SD, standard deviation; TAR, time above range; TBR, time below range; TIR, time in range; TITR, time in tight range.

Supplemental Table S9. Longitudinal changes in glycaemic and patient-reported outcomes in adults with type 1 diabetes undergoing 18 weeks of systematic intensive therapy (SIT) versus conventional therapy (PP2 population).

	SIT (n=49)	Conventional Therapy (n=45)	Adjusted mean difference (95% CI)	*P*
Change from Baseline to 18 weeks				
HbA1c (mmol/mol)	−10·63 (9·64)	−2·47 (6·44)	−7·98 (−10·7, −5·23)	<0·0001
TIR (% of time 3·9–10 mmol/L)	12·28 (13·85)	0·33 (14·04)	10·64 (5·62, 15·66)	<0·0001
Mean glucose (mmol/L)	−1·30 (1·67)	0·12 (1·48)	−1·36 (−1·91, −0·81)	<0·0001
TAR (% of time >10 mmol/L)	−13·41 (15·03)	0·18 (14·78)	−12·3 (−17·7, −6·94)	<0·0001
TBR (% of time <3·9 mmol/L)	0·52 (3·57)	−0·71 (2·67)	1·54 (0·45, 2·63)	0·0060
TITR (% of time 3·9–7·8 mmol/L)	8·62 (11·23)	−0·34 (11·08)	8·57 (4·93, 12·22)	<0·0001
TBR level 2 (% of time <3·0 mmol/L)	−0·10 (2·59)	−0·45 (1·26)	0·76 (0·20, 1·31)	0·0079
TAR level 2 (% of time >13·9 mmol/L)	−10·16 (12·63)	1·24 (11·61)	−10·1 (−14·5, −5·62)	<0·0001
SD of glucose values (mmol/L)	−0·29 (0·88)	−0·02 (0·56)	−0·27 (−0·55, 0·01)	0·055
CV of glucose values (%)	0·62 (6·81)	−0·98 (4·95)	1·95 (−0·02, 3·93)	0·053
MAGE (mmol/L)	−0·83 (2·00)	0·04 (1·39)	−0·80 (−1·44, −0·16)	0·015
DTSQs*	5·59 (5·27)	1·60 (4·86)	3·13 (1·27, 4·98)	0·0012
DTSQc†	14·60 (2·90)	9·70 (6·07)	4·93 (3·18, 6·68)	<0·0001
Hypoglycaemia confidence scale (HCS)‡	0·20 (0·46)	0·19 (0·39)	0·05 (−0·09, 0·20)	0·48
Diabetes distress scale (DDS)§	−0·31 (0·52)	−0·06 (0·39)	−0·26 (−0·44, −0·08)	0·0055
Change from Baseline to 32 weeks				
HbA1c (mmol/mol)	−7·90 (9·20)	−3·76 (8·14)	−3·97 (−7·04, −0·90)	0·012
TIR (% of time 3·9–10 mmol/L)	7·84 (14·45)	−0·15 (11·74)	6·91 (1·88, 11·93)	0·0076
Mean glucose (mmol/L)	−0·75 (1·79)	0·05 (1·41)	−0·74 (−1·31, −0·16)	0·013
TAR (% of time >10 mmol/L)	−8·28 (16·21)	0·33 (12·29)	−7·54 (−13·1, −2·03)	0·0079
TBR (% of time <3·9 mmol/L)	−0·25 (5·08)	−0·35 (2·46)	0·41 (−0·99, 1·81)	0·56
TITR (% of time 3·9–7·8 mmol/L)	4·81 (12·17)	−0·06 (9·39)	4·54 (0·65, 8·43)	0·023
TBR level 2 (% of time <3·0 mmol/L)	−0·38 (3·43)	−0·25 (1·40)	0·26 (−0·52, 1·04)	0·051
TAR level 2 (% of time >13·9 mmol/L)	−6·43 (13·26)	1·06 (11·12)	−6·13 (−10·6, −1·68)	0·0076
SD of glucose values (mmol/L)	−0·16 (0·83)	0·03 (0·61)	−0·18 (−0·47, 0·11)	0·22
CV of glucose values (%)	−0·14 (5·44)	−0·01 (5·54)	0·13 (−1·90, 2·16)	0·90
MAGE (mmol/L)	−0·37 (1·96)	0·32 (1·66)	−0·63 (−1·36, 0·10)	0·090
DTSQs*	4·01 (5·77)	1·05 (5·20)	2·11 (0·02, 4·19)	0·048
Hypoglycaemia confidence scale (HCS)‡	0·23 (0·50)	0·09 (0·36)	0·18 (0·04, 0·33)	0·013
Diabetes distress scale (DDS)§	−0·23 (0·51)	−0·11 (0·38)	−0·14 (−0·31, 0·03)	0·10
Change from Baseline to 52 weeks				
HbA1c (mmol/mol)	−6·45 (10·55)	−2·69 (11·22)	−3·60 (−7·75, 0·54)	0·088
TIR (% of time 3·9–10 mmol/L)	6·55 (14·80)	0·67 (13·63)	5·06 (−0·69, 10·81)	0·084
Mean glucose (mmol/L)	−0·50 (1·86)	0·05 (1·43)	−0·51 (−1·17, 0·15)	0·13
TAR (% of time >10 mmol/L)	−6·53 (17·08)	−0·08 (14·26)	−5·55 (−11·8, 0·70)	0·081
TBR (% of time <3·9 mmol/L)	−0·65 (4·90)	−0·95 (2·42)	0·66 (−0·59, 1·92)	0·30
TITR (% of time 3·9–7·8 mmol/L)	4·47 (11·97)	−0·05 (11·29)	4·24 (−0·24, 8·72)	0·063
TBR level 2 (% of time <3·0 mmol/L)	−0·65 (2·96)	−0·34 (1·07)	0·06 (−0·53, 0·64)	0·84
TAR level 2 (% of time >13·9 mmol/L)	−5·01 (14·01)	0·22 (11·68)	−4·35 (−9·57, 0·86)	0·10
SD of glucose values (mmol/L)	−0·24 (0·98)	−0·14 (0·73)	−0·09 (−0·42, 0·24)	0·59
CV of glucose values (%)	−0·91 (6·17)	−1·72 (5·83)	1·13 (−0·95, 3·22)	0·28

	SIT (n=49)	Conventional Therapy (n=45)	Adjusted mean difference (95% CI)	*P*
MAGE (mmol/L)	−0·59 (1·94)	−0·16 (1·53)	−0·37 (−1·06, 0·32)	0·29
DTSQs*	3·53 (5·33)	1·38 (5·36)	1·31 (−0·67, 3·28)	0·19
Hypoglycaemia confidence scale (HCS)‡	0·24 (0·64)	0·20 (0·42)	0·10 (−0·09, 0·28)	0·30
Diabetes distress scale (DDS)§	−0·25 (0·50)	−0·16 (0·42)	−0·11 (−0·28, 0·06)	0·19

Descriptive data are presented as means and standard deviations.
Statistical analyses were performed using analysis of covariance (ANCOVA), adjusting for baseline values. Robust (heteroscedasticity-consistent) standard errors were employed for non-normally distributed variables (TAR, TBR, DTSQs, HCS, and DDS). Missing data was handled using multiple imputation. Results are presented as adjusted mean differences with 95% confidence intervals (CIs).
PP2 included participants meeting PP1 criteria who also attended visits at weeks 32 and 52 and provided HbA1c data at all follow-ups.
*The DTSQs ranges from 0 to 36, with higher scores reflecting greater treatment satisfaction.
†The DTSQc ranges from −18 to +18, with higher scores reflecting greater treatment satisfaction.
‡The HCS ranges from 1 to 4, with higher scores indicating greater confidence in managing hypoglycaemia.
§The DDS ranges from 1 to 6, with higher scores indicating greater diabetes-related distress.
Abbreviations: CI, confidence interval; CV, coefficient of variation; DDS, diabetes distress scale; DTSQc, diabetes treatment satisfaction questionnaire, change version; DTSQs, diabetes treatment satisfaction questionnaire, status version; HbA1c, glycated haemoglobin A1c; HCS, hypoglycaemia confidence scale; MAGE, mean amplitude of glycaemic excursions; SD, standard deviation; TAR, time above range; TBR, time below range; TIR, time in range; TITR, time in tight range.

Supplemental Table S10. Sensitivity analysis of primary and secondary endpoints using linear mixed effects models, accounting for clustering by study site (ITT population).

	SIT (n=59)	Conventional Therapy (n=58)	Adjusted mean difference (95% CI)	*P*	ICC before MI	ICC after MI
Change from baseline to 18 weeks						
HbA1c (mmol/mol)	−10·6 (9·4)	−2·4 (8·4)	−8·2 (−11·0, −5·4)	<0·0001	0·06	0·07
TIR (% of time 3·9–10 mmol/L)	12·2 (13·4)	1·3 (13·8)	10·1 (5·4, 14·8)	<0·0001	0·08	0·09
Mean glucose (mmol/L)	−1·4 (1·6)	−0·1 (1·5)	−1·3 (−1·8, −0·8)	<0·0001	0·10	0·05
TAR (% of time >10 mmol/L)	−13·2 (14·7)	−1·4 (14·6)	−11·2 (−16·8, −5·6)	0·00014	0·01	0·09
Change from baseline to 32 weeks						
HbA1c (mmol/mol)	−7·5 (9·3)	−3·7 (9·4)	−3·7 (−6·6, −0·8)	0·014	0·19	0·18
Change from baseline to 52 weeks						
HbA1c (mmol/mol)	−6·1 (10·5)	−2·2 (12·2)	−3·8 (−7·6, −0·0)	0·048	0·24	0·20

Descriptive data are presented as means and standard deviations.
Statistical analyses were conducted using linear mixed-effects models, adjusting for baseline values and including study site as a random effect to account for clustering.
Robust (heteroscedasticity-consistent) standard errors were employed for non-normally distributed variables (TAR). Missing data was handled using multiple imputation. Results are presented as adjusted mean differences with 95% confidence intervals (CIs).
Intra-class correlation coefficients (ICCs), reflecting clustering by study site, are reported both before and after multiple imputation.
Abbreviations: CI, confidence interval; HbA1c, glycated haemoglobin A1c; ICC, intra-class correlation coefficient; TAR, time above range; TIR, time in range.

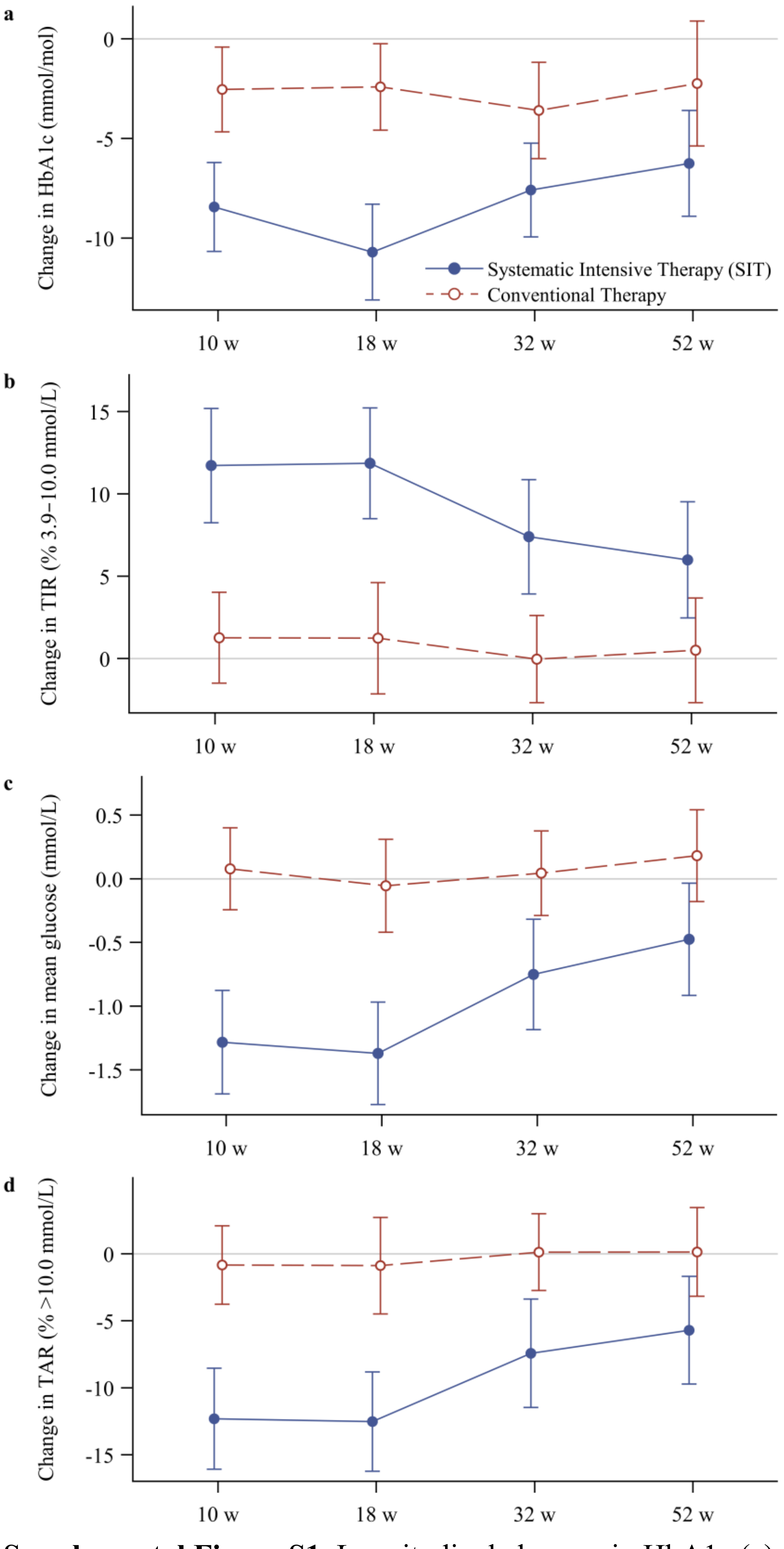

Supplemental Figure S1. Longitudinal changes in HbA1c (a), time in range (TIR; b), mean glucose (c), and time above range (TAR; d) with SIT compared to conventional therapy. Mean values with 95% confidence intervals are shown as solid lines with error bars for the SIT group (blue circles) and dashed lines with error bars for the conventional therapy group (red open circles). The positive effects of SIT observed at the end of treatment (week 18) were sustained across all endpoints at 32 weeks and for mean glucose at 52 weeks.

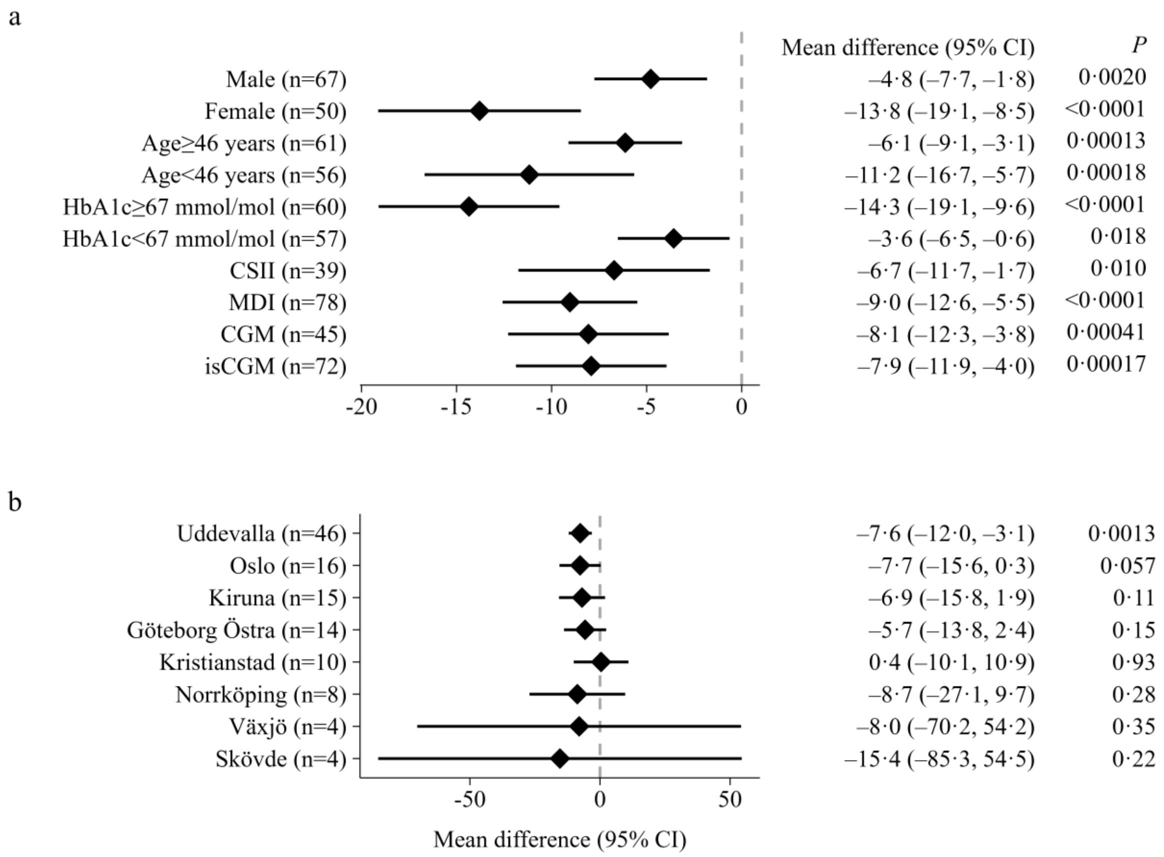

Supplemental Figure S2. Subgroup analyses of the change in HbA1c from baseline to 18 weeks across minimisation variables (a) and study site (b) in the ITT population.

Symbols represent adjusted mean differences in HbA1c (mmol/mol) between the systematic intensive therapy (SIT) group and the conventional therapy group, with horizontal error bars indicating 95% confidence intervals. Estimates were obtained using analysis of covariance (ANCOVA), adjusting for baseline HbA1c values. Negative values indicate greater reductions in HbA1c in the SIT group relative to the control group.

Abbreviations: CGM, continuous glucose monitoring; CSII, continuous subcutaneous insulin infusion; HbA1c, glycated haemoglobin; isCGM, intermittently scanned continuous glucose monitoring; MDI, multiple daily injections.

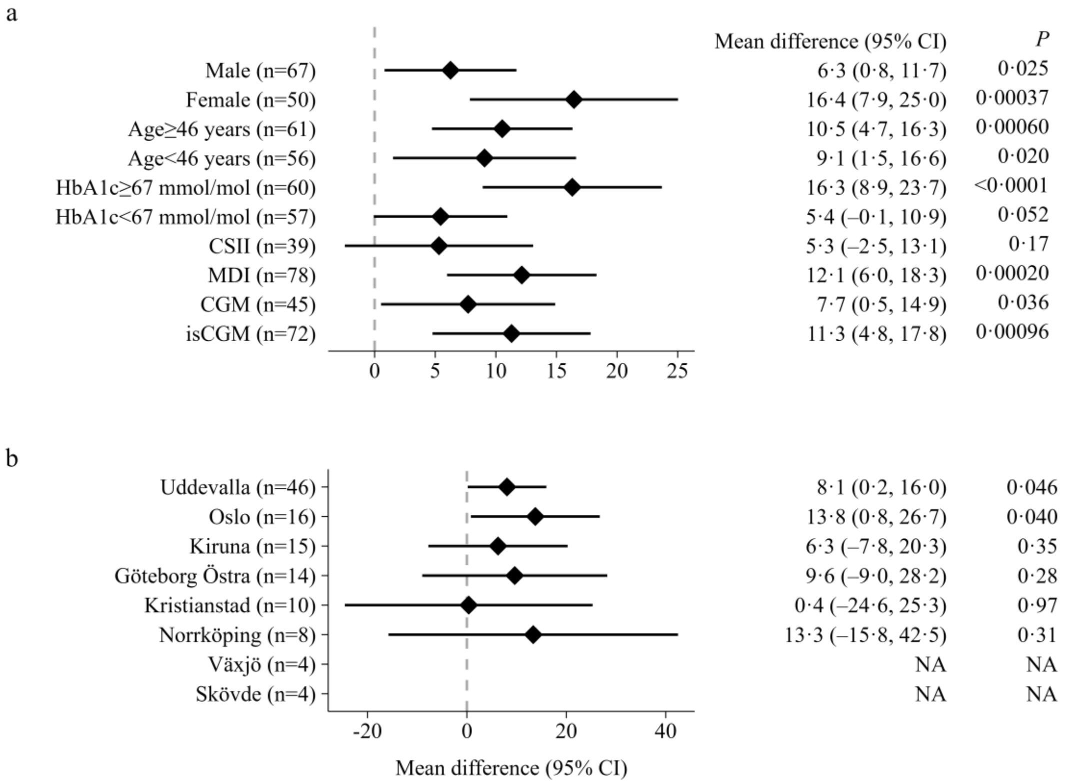

Supplemental Figure S3. Subgroup analyses of the change in time in range (TIR) from baseline to 18 weeks across minimisation variables (a) and study site (b) in the ITT population.

Symbols represent adjusted mean differences in TIR (% of time within 3·9–10 mmol/L) between the systematic intensive therapy (SIT) group and the conventional therapy group, with horizontal error bars indicating 95% confidence intervals. Estimates were obtained using analysis of covariance (ANCOVA), adjusting for baseline TIR. Positive values indicate greater improvements in TIR in the SIT group relative to the control group.

Abbreviations: CGM, continuous glucose monitoring; CSII, continuous subcutaneous insulin infusion; HbA1c, glycated haemoglobin; isCGM, intermittently scanned continuous glucose monitoring; MDI, multiple daily injections; NA, not available due to limited data.

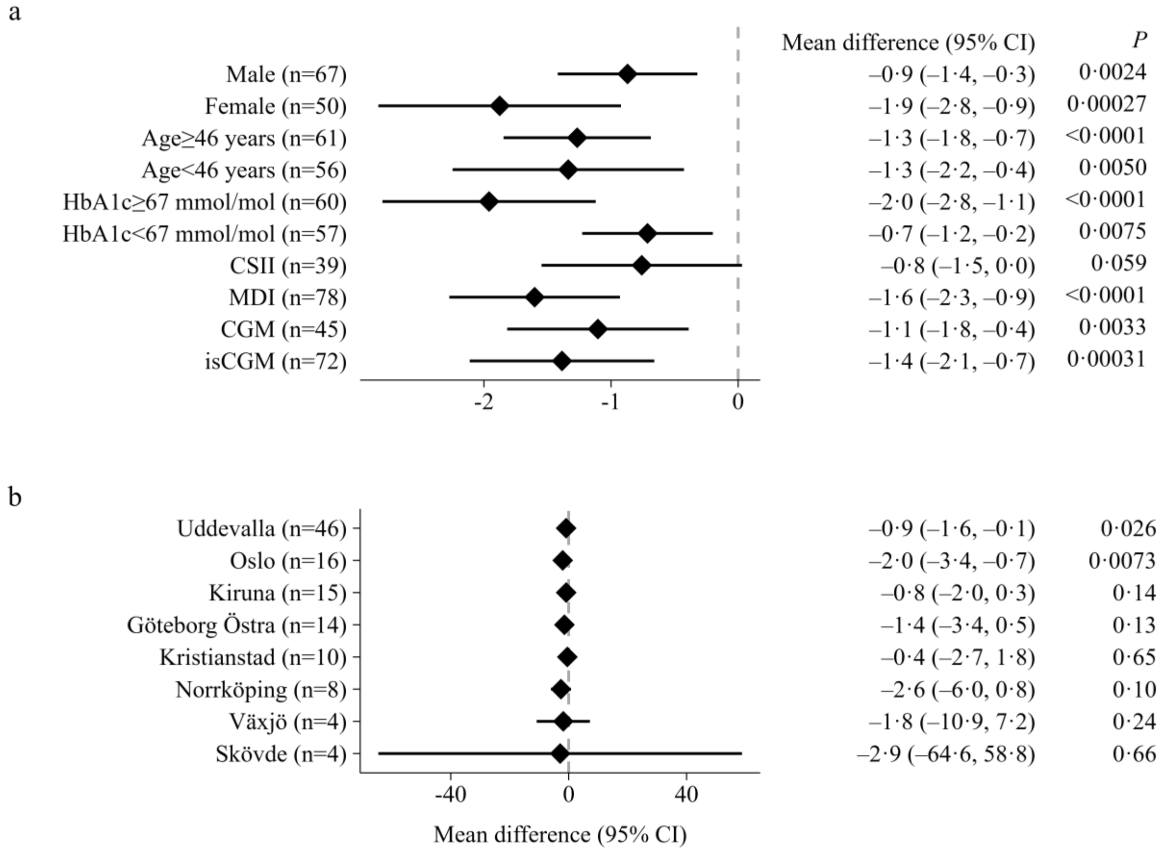

Supplemental Figure S4. Subgroup analyses of the change in mean glucose from baseline to 18 weeks across minimisation variables (a) and study site (b) in the ITT population.

Symbols represent adjusted mean differences in mean glucose (mmol/L) between the systematic intensive therapy (SIT) group and the conventional therapy group, with horizontal error bars indicating 95% confidence intervals. Estimates were obtained using analysis of covariance (ANCOVA), adjusting for baseline mean glucose. Negative values indicate greater reductions in mean glucose in the SIT group relative to the control group.

Abbreviations: CGM, continuous glucose monitoring; CSII, continuous subcutaneous insulin infusion; HbA1c, glycated haemoglobin; isCGM, intermittently scanned continuous glucose monitoring; MDI, multiple daily injections.

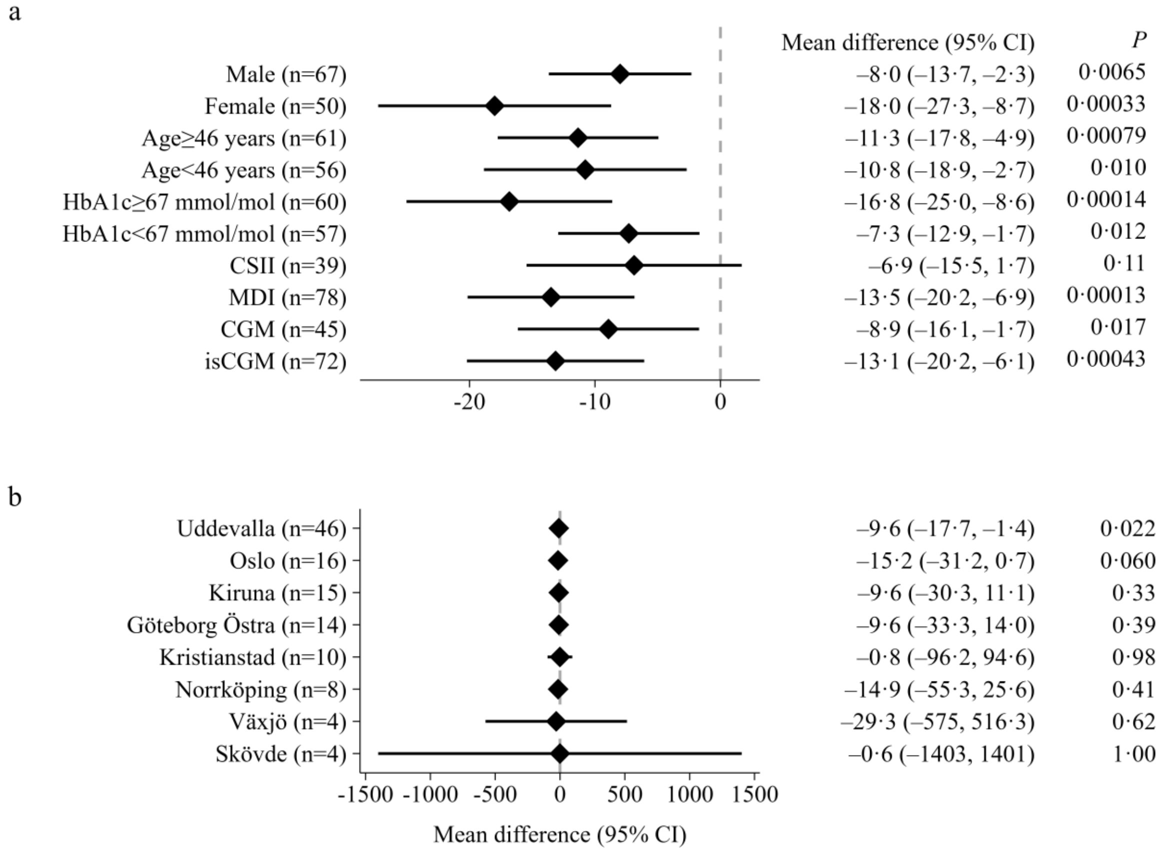

Supplemental Figure S5. Subgroup analyses of the change in time above range (TAR) from baseline to 18 weeks across minimisation variables (a) and study site (b) in the ITT population.

Symbols represent adjusted mean differences in TAR (% of time >10·0 mmol/L) between the systematic intensive therapy (SIT) group and the conventional therapy group, with horizontal error bars indicating 95% confidence intervals. Estimates were obtained using analysis of covariance (ANCOVA), adjusting for baseline TAR. Negative values indicate greater reductions in TAR in the SIT group relative to the control group.

Abbreviations: CGM, continuous glucose monitoring; CSII, continuous subcutaneous insulin infusion; HbA1c, glycated haemoglobin; isCGM, intermittently scanned continuous glucose monitoring; MDI, multiple daily injections.

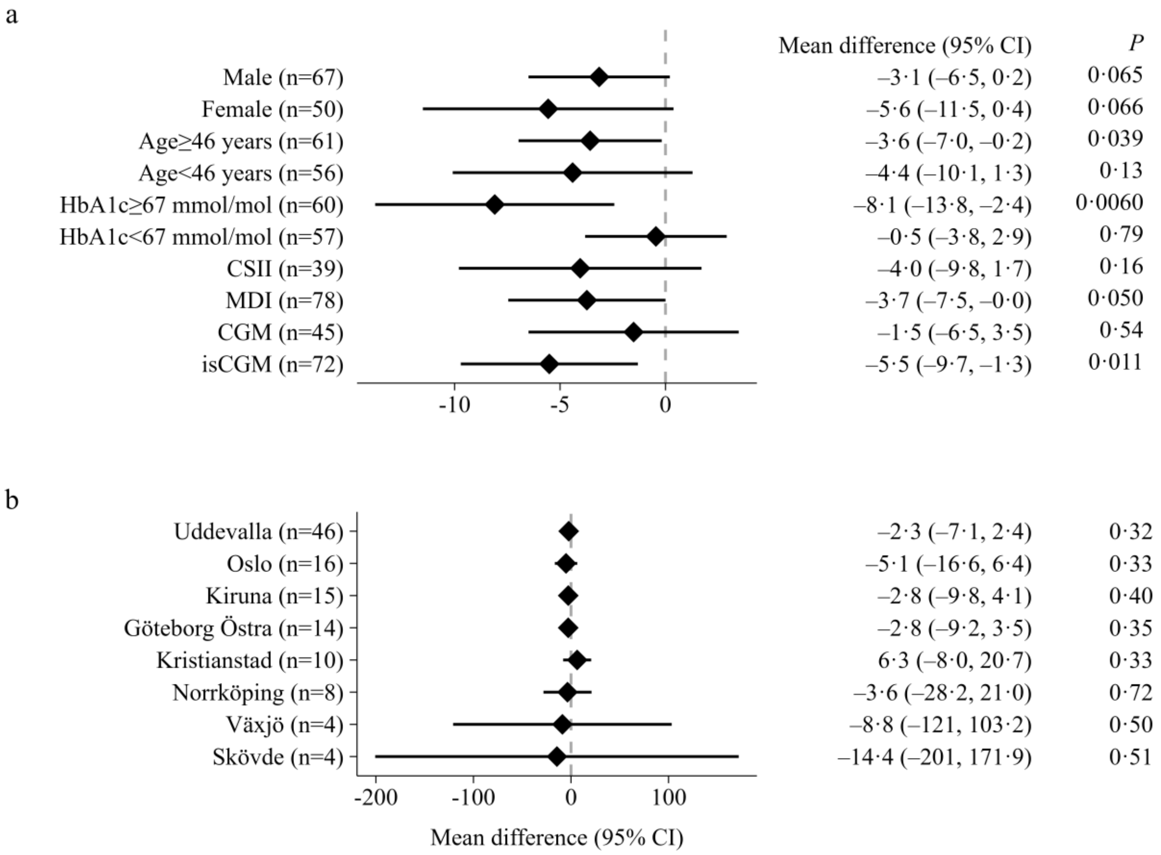

Supplemental Figure S6. Subgroup analyses of the change in HbA1c from baseline to 32 weeks across minimisation variables (a) and study site (b) in the ITT population.

Symbols represent adjusted mean differences in HbA1c (mmol/mol) between the systematic intensive therapy (SIT) group and the conventional therapy group, with horizontal error bars indicating 95% confidence intervals. Estimates were obtained using analysis of covariance (ANCOVA), adjusting for baseline HbA1c values. Negative values indicate greater reductions in HbA1c in the SIT group relative to the control group.

Abbreviations: CGM, continuous glucose monitoring; CSII, continuous subcutaneous insulin infusion; HbA1c, glycated haemoglobin; isCGM, intermittently scanned continuous glucose monitoring; MDI, multiple daily injections.

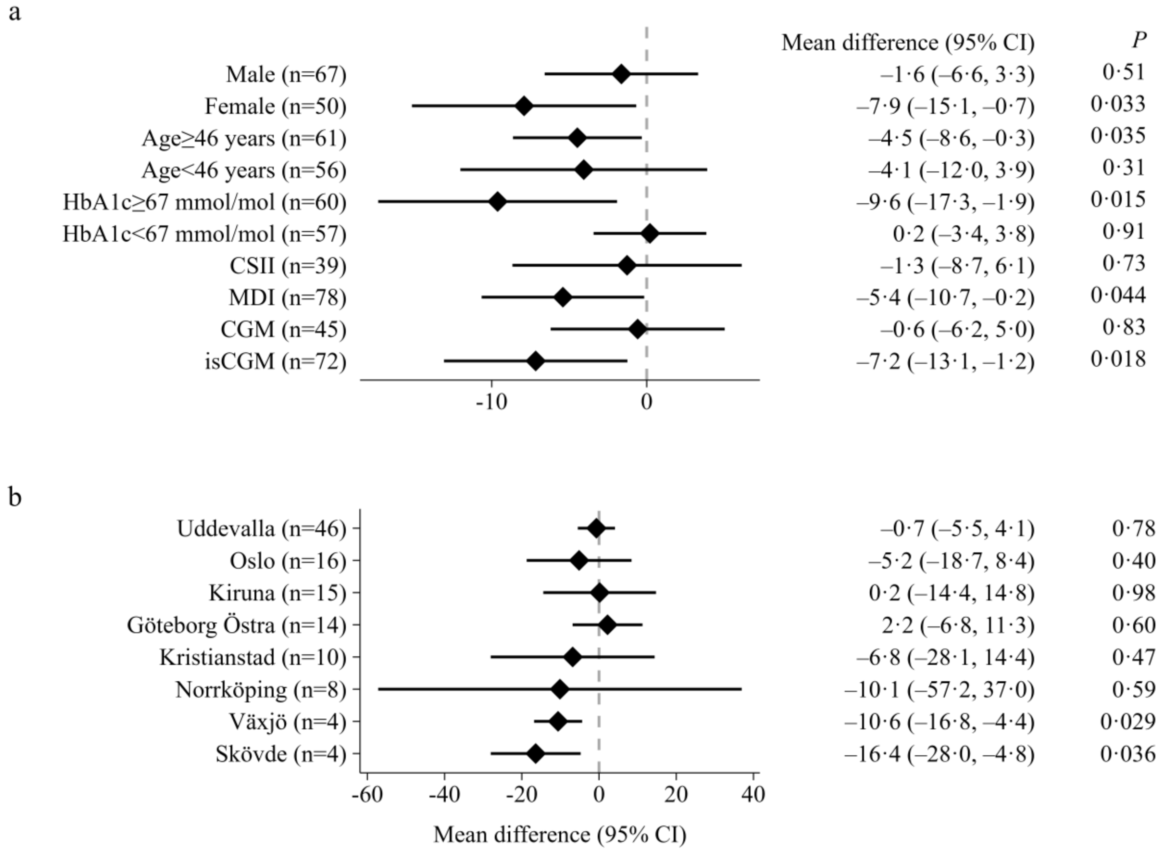

Supplemental Figure S7. Subgroup analyses of the change in HbA1c from baseline to 52 weeks across minimisation variables (a) and study site (b) in the ITT population.

Symbols represent adjusted mean differences in HbA1c (mmol/mol) between the systematic intensive therapy (SIT) group and the conventional therapy group, with horizontal error bars indicating 95% confidence intervals. Estimates were obtained using analysis of covariance (ANCOVA), adjusting for baseline HbA1c values. Negative values indicate greater reductions in HbA1c in the SIT group relative to the control group.

Abbreviations: CGM, continuous glucose monitoring; CSII, continuous subcutaneous insulin infusion; HbA1c, glycated haemoglobin; isCGM, intermittently scanned continuous glucose monitoring; MDI, multiple daily injections.
