# Supplementary material for: Systematic intensive therapy in addition to continuous glucose monitoring in adults with type 1 diabetes: a multicentre, open-label, randomised controlled trial
Source: Lancet Reg Health Eur. 2025 Oct 16;59:101485. doi: 10.1016/j.lanepe.2025.101485 (PMC12553072; doi:10.1016/j.lanepe.2025.101485)
Supplement: Protocol SIT [file mmc2.pdf]

1 Title Page

Protocol Number:	Systematic Intensive Therapy
Protocol Title:	A randomised trial of evaluating a systematic intensive therapy using Continuous Glucose Monitoring (CGM) and Flash Glucose Monitoring (FGM) in clinical diabetes care
Sponsor:	Researcher initiated trial. Sponsor is: NU-Hospital Group
Intervention	Systematic intensive therapy through internet or telephone
Protocol Release Date:	13. September 2018
GCP Statement:	This study is to be performed in full compliance with ICH and all applicable local Good Clinical Practices (GCP) and regulations. All required study documentation will be archived as required by competent authorities.

2 Protocol Synopsis/Summary

Good glycaemic control is important to reduce diabetes complications for persons with type 1 diabetes. Earlier study have shown that both Continuous glucose monitoring (CGM) and Flash glucose monitoring (FGM) improves glycaemic control.

This study aims to analyse if intensive systematic treatment via internet and telephone during 4 months improved HbA1c for persons with type 1 diabetes which are already treated with CGM or FGM, and if the effect continuous for 1-2 years after the intervention stops. Time in Hypoglycaemia, treatment satisfaction, Diabetes distress and hypoglycaemia fear will even be analysed.

A randomised, non-blinded, multi-centre, clinical study for persons with type 1 diabetes and hbA1c ≥ 58mmol/mol and treated with CGM or FGM.

The control group continuous its normal diabetes care with study visits at randomisation, 10, 18, 32, and 52 weeks for HbA1c and to fill in questionnaires.

The intervention group will have contact with the study team on a weekly basis, when mean blood glucose levels the previous week are elevated. They will receive help with analysing data and advice on how to improve their glucose values. They will even meet the study team at randomisation, 10, 18, 32, and 52 weeks for HbA1c and to fill in questionnaires.

Table of Contents

1 Title Page ...1

2 Protocol Synopsis/Summary ...2

3 List of Abbreviations & Definition of Terms ...7

4 Study Conduct & Oversight ...8

4.1 Sponsor and Coordinating Investigator ...8

4.2 Executive committee ...8

4.3 Site Monitoring ...8

4.4 Data Management & Statistics ...8

4.5 Lab ...8

4.6 Investigators & sites ...8

5 Introduction & Background Information ...9

5.1 Background ...9

5.2 Study population ...10

6 Trial Objectives ...10

6.1 Purpose/aim of the study ...10

6.2 Primary Objective ...10

6.3 Secondary Objectives ...10

7 Trial Design ...11

7.1 Design ...11

7.2 Treatments ...11

7.2.1 Conventional Care ...11

7.2.2 Intensive diabetes care ...11

7.3 Randomisation ...12

7.4 Duration ...13

7.5 Endpoints 13
7.5.1 Primary 13
7.5.2 Secondary 13
8 Selection and Withdrawal of Subjects 13
8.1 Inclusion & Exclusion criteria 13
8.1.1 Inclusion Criteria: 13
8.1.2 Exclusion criteria: 14
8.2 Criteria for Withdrawal 14
9. Trial Procedures 15
9.1 Information 16
9.2 Inclusion 16
9.3 Randomisation visit 16
9.4 Clinical Visits 16
10 Importance 17
10. Assessment of Efficacy 17
10.1 Primary efficacy variable 17
10.2 Secondary efficacy variables 17
11. Assessment of Safety 17
11.1 Hypoglycaemias and other adverse events. 17
11.1.1 Reporting of Adverse Events (AE) 18
11.2 Serious Adverse Events (SAE) 18
11.2.1 Definition of SAE 18
11.2.2 Reporting of SAE 18
12. Statistics 19
12.1 Populations 19

12.1.1 Intention to treat population (ITT) 19
12.1.2 Per-Protocol population 19
12.1.3 Safety Population 19
12.2 Descriptive statistics 19
12.3 Baseline table 19
12.4 Efficacy analyses 19
12.4.1 Primary efficacy analysis 19
12.4.2 Secondary efficacy analyses 19
12.5 Statistical Analysis Plan 20
12.6 Sample Size Calculation 20
12.7 Safety analyses 20
13. Premature termination of the trial 21
14. Data Handling and Record Keeping 21
14.1 Data Collection 21
14.2 Data Management 21
14.2.1 Study Database & Data Entry 21
14.2.2 Data Validation & Data Clarifications 21
14.2.3 Clean File & Database Lock 21
14.3 Data Retention & Archiving 22
15. Access to Source Data/Documents 22
16. Quality Control and Quality Assurance 22
17. Ethics 22
17.1 Declaration of Ethical Conduct 22
17.2 Ethical Review 23
17.3 Subject Information & Consent 23

18. Protocol Adherence & Amendments 24
18.1 Adherence to Protocol 24
18.2 Protocol Amendments 24
19. Financing and Insurance 25
20. Compensation 25
21. Publication Policy 25
22. Supplements/Appendices 25
22.1 Amendments 25
22.2 Personnel Information 25
23. References 26

3 List of Abbreviations & Definition of Terms

HbA1c = Glycated hemoglobin

SD = Standard deviation

MDI = Multiple daily insulin injections = Basal insulin + meal time insulin to at least all major meals

CGM = Continuous glucose monitoring

FGM = Flash Glucose Monitoring

AE = Adverse event

SAE = Serious adverse event

SAP = Statistical Analysis Plan

4 Study Conduct & Oversight

4.1 Sponsor and Coordinating Investigator

This is an investigator-initiated trial. The sponsor is NU-Hospital Group. The Coordinating Investigator is Marcus Lind, MD, PhD, Sahlgrenska Academy, University of Gothenburg, Gothenburg, Sweden and NU-Hospital Organization, Uddevalla, Sweden.

4.2 Executive committee

Marcus Lind, MD, PhD, Associate Professor
Department of Molecular and Clinical Medicine, Sahlgrenska Academy University of Gothenburg, Gothenburg, Sweden and NU-Hospital Organization, Uddevalla, Sweden

Arndís F. Ólafsdóttir RN, PgD
Department of Molecular and Clinical Medicine, Sahlgrenska Academy University of Gothenburg, Gothenburg, Sweden and NU-Hospital Organization, Uddevalla, Sweden

4.3 Site Monitoring

Gothia Forum, Gothenburg, Sweden

4.4 Data Management & Statistics

An eCRF-system will be used in the study. Data Management (including the randomization system) will be handled by dSharp Consulting, Gothenburg, Sweden. Statistical analyses will be performed by Statistiska Konsultgruppen, Gothenburg, Sweden.

4.5 Lab

The only laboratory test that will be performed is control of HbA1c which will be made locally with a method calibrated and controlled to Equalis.

4.6 Investigators & sites

Marcus Lind, MD, PhD, Diabetologist, Diabetes Section Uddevalla Hospital, Uddevalla, Sweden

Ulf Rosengren, MD, Diabetes clinic, Hospital in Motala, Motala, Sweden

Thomas Nyström, MD, Department of Medicine, Diabetes, Södersjukhuset, Stockholm, Sweden

Magnus Wijkman, MD, Diabetes Clinic, Vrinnevihospital Norrköping, Norrköping, Sweden

Per-Anders Jansson MD, Gothia Forum, Sahlgrenska Universitetssjukhus, Göteborg, Sweden

Erik Schwarcz, MD, Örebro Diabetes clinic, University Hospital, Örebro, Sweden

5 Introduction & Background Information

5.1 Background

Good glycaemic control is a key stone for reducing long-term diabetes complications in persons with type 1 diabetes (1). During the last few years continuous glucose monitoring (CGM) has shown to be an efficient way to improve glycaemic control in persons treated with insulin pumps (2). Recently it was shown in a multicentre randomised trial, the GOLD study, that CGM also improves the glycaemic control in persons with T1D treated with multiple daily insulin injections [MDI,(3)]. In addition time in hypoglycaemia was reduced and we could for the first time show that CGM improved quality of life and hypoglycaemia confidence. Flash Glucose Monitoring (FGM) has also recently been shown to efficiently reduce time in hypoglycaemia (4).

CGM is a subcutaneous sensor estimating blood glucose levels continuously and displaying them on a small monitor or on a mobile phone. CGM also informs of glucose trends and alarms the patient for low or high glucose levels(5). FGM is a subcutaneous sensor which is placed on the upper arm and needs to be scanned by small monitor or by a mobile phone to receive estimated blood glucose levels and how the current trends are(6).

In spite of CGM and FGM being used by approximately 50% of patients in Sweden, the majority of patients do still not reach good glycaemic control associated with a low risk of diabetes complications (7). This is in line with effects in clinical trials, although CGM efficiently reduces HbA1c the effects have been around 0.4% and far from enough for solving the problem of poor glycaemic control for most patients.

However, in earlier studies patients have generally just been educated about the systems and then mainly used them by themselves with regular clinical visits as support. It is possible that the effects of FGM and CGM on glycaemic control could be much greater with a more extensive clinical support. CGM/FGM could also be used as a tool for motivation and communication with caregivers. It is possible that diabetes care should translate into another type of assistance to patients rather than the current system of having clinical visits every 3-6 months.

Today FGM and CGM data can be transferred to the caregiver via electronic communication. This opens up several opportunities such as discussion and support of glucose values and trends in close connection to daily living. The question is whether such an approach would be more fruitful than only using regular clinical visits and what effects could be obtained. If the mean blood glucose levels are elevated, glucose data can be transferred on a weekly basis to the diabetes nurse or doctor for guidance. Besides assistance such an approach may enhance motivation. Specific individual targets could be set for each participant depending on how far over the recommended guidelines they lie. This way they can reach several partial goals before reaching their final goal.

Today, diabetes-care teams in many countries are not constructed for supporting approaches via telephone contacts or other media financially. However, if a more efficient care could be shown,

financial support would likely be given and routines for registering financial costs of work changed.

5.2 Study population

The study will be performed at 6 clinics in Sweden including a total of 142 individuals. Patients with type 1 diabetes with HbA1c ≥ 58 mmol/mol, currently using CGM or FGM and who have the possibility to download their devices at home will be included. Patients will be recruited at each site.

6 Trial Objectives

6.1 Purpose/aim of the study

The aim of the study is to evaluate whether a close collaboration using FGM and CGM data with diabetes-care teams will improve glycaemic control in persons with type 1 diabetes. We will also evaluate whether such an approach has a sustained effect on glycaemic control after it is discontinued.

6.2 Primary Objective

The primary objective is to evaluate whether systematic intensive therapy using FGM and CGM data with diabetes care teams will improves glycaemic control (measured by HbA1c at baseline and after 18 weeks) compared to conventional care in persons with type 1 diabetes with impaired glycaemic control during a 18 week period.

6.3 Secondary Objectives

Secondary objectives are comparison of the following variables between patients with type 1 diabetes randomised to systematic intensive therapy or conventional care:

- HbA1c at 32 weeks
- HbA1c at 52 weeks
- Mean glucose levels at 18, 32, and 52 weeks.
- Glycaemic variability by Standard Deviation, CV and MAGE at 18, 32, and 52 weeks.
- Time in Hypoglycaemia at 18, 32, and 52 weeks,
- Time in Hyperglycaemia 18, 32, and 52 weeks
- Hypoglycaemia Confidence (Hypoglycaemia confidence scale) at 18, 32, and 52 weeks
- Diabetes distress (DDS-questionnaire) at 18, 32, and 52 weeks
- Treatment satisfaction (DTSQs and c questionnaire) at 18, 32, and 52 weeks

7 Trial Design

7.1 Design

Non-blinded, multi-centre randomised clinical trial, parallel design, a 52-week follow-up study.

7.2 Treatments

7.2.1 Conventional Care

Patients will come for a visit 10, 18, 32 and 52 weeks after randomisation for measurement of HbA1c, downloading of CGM/FGM curves and during weeks 18, 32, and 52 they will fill in questionnaires registered at baseline, the visits week 10 and 18 will take place exact 10 and 18 weeks after randomisation + - 1 week, and vists week 32 and 52 will take place exact 32 and 52 weeks after randomisation + - 2 weeks.

7.2.2 Intensive diabetes care

Patients randomised to systematic intensive therapy will continue to follow their regular planned clinical visits and contacts. All patients will be taught how to download CGM/FGM-data on their home computer/lap-top. They will be taught how to use the software suitable for their device for interpretation of data. They will be taught how to interpret their data for patterns such as:

A) High/low overnight and morning profile
B) Excursions before and after meals
C) Timing of insulin in relation to mealtimes and exercise.
D) Time in various glycaemic ranges and interpretation of glycaemic variability by the standard deviation

In most instances it will be possible to use the Diasend software, but for some CGM systems specific software's will be needed as for example care-link for Medtronic products.

The relationship between mean glycaemic control and HbA1c will be discussed and explained, patients will receive a graph which depicts this relationship.

An individual HbA1c target will be discussed and a goal set for mean glucose levels relating to this HbA1c target. The mean glucose goal will also be discussed in relation to the current mean glucose level at randomisation. The patients will be taught how to download their device on the first visit and will get assistance if necessary. If needed, further support can be given in the beginning via telephone or internet as patients download their devise at home.

A first telephone contact will be planned up to and no later than 1 week after randomisation. The patient will be expected to have downloaded their device prior to this contact so both care-giver and patient have access to all blood glucose profile for the past week. The visit will take place the same day each week +- 1 day.

A joint analysis will be performed on the glucose profiles. Analysis will be done in relation to mean glucose levels, standard deviation, before bedtime data, overnight glucose profile, blood glucose levels before and after meals and exercise, time in hypoglycaemia and the patient will even be able to discuss any particular situations that have proven more difficult than others.

Using their unique expertise the care-giver will be expected to make an overall judgement regarding improvements for the patient.

If the patient has reached its first mean blood glucose goal but not the recommended goal of 8,4mmol/l, a new target will be decided on. The recommended mean glucose level of 8,4mmol/l gives an estimated HbA1c level of 52 mmol/mol which is the national recommendation in Sweden. Therefore, if patients reaches a mean blood glucose of 8,4mmol/l, they are expected to have an HbA1c level which are associated with a lower risk of complications.

During the first 4 weeks a telephone contact will be made on a weekly basis. After these 4 weeks it might be actual to decrease this to every second week depending on mean glucose levels.

If the patient reaches the recommended goal of mean blood glucose < 8,4 mmol/l no telephone contact will be made that week but data needs to be downloaded again the following week and new contact made if mean glucose has risen above 8,4 mmol/l.

A clinical visit will be scheduled after 10 and 18 weeks (same time as for the control group) to evaluate effects on HbA1c and other glycaemic variables (mean glucose levels, SD of glucose levels, time in hypo and hyperglycaemia and time in range).

6.5.3 Follow-up phase

After the 18 weeks patients will return to their normal schedule at their diabetes clinic but HbA1c will be controlled again at 32 and 52 weeks. No intervention will be done during this time. Patients will be encouraged to and hopefully will continue to download their devices and to do their own analysis of their blood glucose profiles as they have learned during the intervention. If patients actively contact their team due to technical problems regarding the downloading of their devises or specific questions regarding their analysis, support and advice will be given but there will be no further planned contact.

7.3 Randomisation

Subjects will be randomised to systematic intensive therapy or conventional care. Minimisation (optimal allocation) using a centralised web system (handled by dSharp Consulting) will be used for randomisation. At randomisation each subject will be assigned a unique and anonymous Subject ID.

7.4 Duration

The expected duration of subject participation is 18 weeks, with follow up measurements at weeks 32 and 52. The expected total study duration, from first patient in to last patient out, is approximately 2 years (recruitment is judged to take 1 year).

7.5 Endpoints

7.5.1 Primary

Primary endpoint will be change in HbA1c from baseline to week 18.

7.5.2 Secondary

Secondary endpoints will be:

- change in HbA1c from baseline to week 18
- change in HbA1c from baseline to week 52
- Change in Time in Range (4-10mmol/l) and Time in Target (4-8mmol/l) from Baseline to week 18
- Change in Time in Range (4-10mmol/l) and Time in Target (4-8mmol/l) from Baseline to week 52
- Change in mean glucose levels from baseline to 18, 32, and 52 weeks
- Change in glycaemic variability from baseline to 18, 32, and 52 weeks
- Change in time in hypoglycaemia from baseline to 18, 32, and 52 weeks
- Change in time in hyperglycaemia from baseline to 18, 32, and 52 weeks
- Change in Hypoglycaemic confidence score from baseline to 18, 32, and 52 weeks
- Change in DDS score from baseline to 18, 32, and 52 weeks
- Change in DTSQs score from baseline to 18, 32, and 52 weeks and DTSQc at 18, 32 and 52 weeks

8 Selection and Withdrawal of Subjects

The study is planned to include 142 subjects randomised 1:1 to systematic intensive care or conventional care. Drop-outs (expected to be maximally 10%) will not be replaced.

8.1 Inclusion & Exclusion criteria

8.1.1 Inclusion Criteria:

- Informed consent obtained before trial-related activities (i.e., any activity that would not have been performed during routine patient management)
- Clinical diagnosis of Type 1 diabetes
- Adult patients over 18 years of age
- HbA1c ≥ 58 mmol/mol
- Currently using CGM or FGM

- To have a possibility to download and share FGM/CGM data

8.1.2 Exclusion criteria:

- Type 2 diabetes
- Diabetes duration <1 year
- Long-term Systemic glucocorticoid treatment during the last 3 months
- Planned or changed treatment the last 3 months regarding MDI vs. Insulin pump or added or stopped CGM or FGM therapy
- Current or planned pregnancy or breastfeeding during the next 12 months
- Planned move during the next 12 months making it not possible to participate in study activities
- Other reason determined by the investigator not being appropriate for participation

8.2 Criteria for Withdrawal

- Subjects may withdraw from participation at will at any time.

9. Trial Procedures

Trial procedures are schematically shown below:

Variables		Visits **				
	Inclusion visit 1 *	Randomisation visit 2	10 week Follow-up visit 3	18 week follow-up visit 4	32 week follow-up visit 5	52 week follow-up visit 6
Visit Window		Scheduled within 28 days after inclusion	+ - 1 weeks	+ - 1 weeks	+- 2 weeks	+ - 2 weeks
Informed Consent	X					
Inclusion/Exclusion Criteria	X					
Demographics, Medical History	X					
Physical Examination	X					X
HbA1c	X	X	X	X	X	X
Download devise	X	X	X	X	X	X
Education on downloading devise		X				
Weight		X		X	X	X
DTSQs		X		X	X	X
DTSQc				X		
DDS scale		X		X	X	X
Hypoglycaemia confidence scale		X		X	X	X
AE (severe hypoglycaemia and diabetes ketoacidosis), SAE		X	X	X	X	X
	* Before the visits in the schedule above patient information will be given either via telephone or at a clinical visit.<br><br>** If randomised to systematic intensive treatment the first telephone contact will take place 1 week after randomisation and after that on a weekly basis or until mean glucose levels reach target.					

9.1 Information

Recruitment visit/Telephone contact: Patients will be given a brief overview of the study either at a clinical visit or via telephone and written information approved by ethical committee sent/given to the patient.

9.2 Inclusion

Patients will be permitted to ask questions about the study after reading the written information and receive further explanation. If the patient gives written and verbal informed consent to participate, inclusion/exclusion criteria will be assessed and a physical examination will be performed. HbA1c will be taken and CGM/FGM downloaded.

9.3 Randomisation visit

Randomization will be made to systematic intensive care or conventional care. The following variables will be recorded:

- HbA1c
- Weight
- Type of insulin and doses of insulin
- Demographics and history of complications
- Type of insulin delivery CSII/MDI
- Type of glucose recording – CGM or FGM
- DTSQs, DDS, and hypoglycaemia confidence scale

Web randomisation will be performed with minimisation (optimal allocation) to systematic intensive therapy or conventional care in 1:1 proportion.

9.4 Clinical Visits

Initial visit with a diabetes nurse will take place at the randomisation visit. Treatment will be initiated in accordance with section 7.2 “Treatment”. At visit week 18, 32, and 52 with the diabetes nurse CGM/FGM will be downloaded and the following variables will be measured:

- HbA1c
- Weight
- Type of insulin and doses of insulin
- AE, SAE
- DTSQs, DTSQc, DDS, and hypoglycaemia confidence scale
- Number of contacts (clinical or telephone) with regular diabetes care team

At visit week 10 with the diabetes nurse CGM/FGM will be downloaded and following variable will be measured:

- HbA1c

At week 52 a physical examination will be performed

10 Importance

It is of great importance to gain knowledge of the potential benefits of the addition of systematic intensive therapy from a diabetes nurse in the type 1 diabetes care. If the treatment improves glycaemic control, diabetes distress or hypoglycaemic confidence this could be a complement to the routine care for diabetes team.

10. Assessment of Efficacy

10.1 Primary efficacy variable

The primary efficacy variable is change in HbA1c from baseline to 18weeks follow-up.

10.2 Secondary efficacy variables

Secondary efficacy variables:

- change in HbA1c from baseline to week 32
- change in HbA1c from baseline to week 52
- Change in mean glucose levels from baseline to 18, 32, and 52 weeks
- Change in glycaemic variability from baseline to 18, 32, and 52 weeks
- Change in time in hypoglycaemia from baseline to 18, 32, and 52 weeks
- Change in time in hyperglycaemia from baseline to 18, 32, and 52 weeks
- Change in Hypoglycaemic confidence score from baseline to 18, 32, and 52 weeks
- Change in DDS score from baseline to 18, 32, and 52 weeks
- Change in DTSQs score from baseline to 18, 32, and 52 weeks and DTSQc at 18, 32 and 52 weeks.

Descriptive efficacy variables will be the above variables at each visit.

11. Assessment of Safety

11.1 Hypoglycaemias and other adverse events.

Non-severe hypoglycaemias will not be recorded in this study. Only severe hypoglycaemias will be recorded. Besides severe hypoglycaemias only keto-acidosis will be recorded regarding AE. In addition all SAE:s will be recorded. Severe hypoglycaemias will be defined as unconsciousness due to low glucose levels or needing assistance to solve a hypoglycaemia.

11.1.1 Reporting of Adverse Events (AE)
AEs according to section 11.1 will be followed up and recorded at visits with the diabetes nurse in the study at visit week 18, 32 and 52.

AEs will be recorded on the AE pages of the eCRF. For each AE, the following information will be recorded:

- Start/stop date
- Severity
- Action taken
- Relationship to study treatment
- Outcome
- Seriousness

11.2 Serious Adverse Events (SAE)

11.2.1 Definition of SAE
An SAE is any medical occurrence at any dose that:

- Results in death
- Is life-threatening (i.e. the subject was at immediate risk of death from the AE as it occurred. This does not include an event that, had it occurred in a more severe form or was allowed to continue, might have caused death)
- Requires inpatient hospitalisation or prolongs existing hospitalisation
- Results in persistent or significant disability/incapacity
- Is a congenital anomaly/birth defect (in the child of a subject who was exposed to the study medication)
- Is a medically important event or reaction (see below)

Other important medical events that may not be immediately life-threatening or result in death or hospitalisation but may, based on appropriate medical judgment, jeopardise the subject or require intervention to prevent one of the outcomes in the definition of SAE listed above should also be considered SAEs. Examples of such events are intensive treatments in an emergency room or at home for allergic bronchospasm, blood dyscrasias or seizures that do not result in hospitalisation, or development of drug dependency or drug abuse. These events may be considered to need rapid reporting by the Sponsor to competent authorities.

11.2.2 Reporting of SAE
All SAEs will be recorded on the AE pages of the CRF. In addition, SAEs must be reported to the sponsor using an SAE Data Form. Subjects with SAEs must be followed until the event resolves, or the event or sequelae stabilise.

12. Statistics

12.1 Populations

12.1.1 Intention to treat population (ITT)

The intention to treat (ITT-population) consists of all randomised patients. The patient belongs to the randomised group irrespective of treatment received.

12.1.2 Per-Protocol population

The Per-Protocol population (PP-population) consists of all patients in the ITT-population without any significant protocol deviations. The PP-population is defined at the clean-file meeting before the database is locked.

12.1.3 Safety Population

The safety population consists of all randomised patients who were treated. In the safety analysis a patient will belong to the treatment given not to the randomised treatment.

12.2 Descriptive statistics

All continuous variables will be summarized with number, mean, SD, median and range and all categorical variables will be summarised with number and percentages.

12.3 Baseline table

All baseline variables will be tabulated and analysed by treatment group.

12.4 Efficacy analyses

12.4.1 Primary efficacy analysis

Primary efficacy analysis will be change in HbA1c from baseline to 18 weeks follow-up between the two treatment groups using analysis of covariance (ANCOVA) with HbA1c at baseline as covariate on the ITT -population, two-sided test and significance level of 0.05. If HbA1c from 18weeks follow-up is missing the last observation carry forward (LOCF) principle from baseline and 10 weeks will be applied. Additionally, the sensitivity analyses using multiple imputation and complete cases will be performed. The assumption of normal distribution will be assessed. The detailed information about statistical methods will be described in the Statistical Analysis Plan (SAP) prior to database lock.

12.4.2 Secondary efficacy analyses

- Change in HbA1c, mean glucose, glycaemic variability and time in hypo- and hyperglycaemia, time in range and time in target will be analysed in similar way as described above for the primary variable.
- Change in the DDS-score from baseline to week 18, 32, 52 between the two treatment groups using ANCOVA with DDS-score at baseline as covariate. In case the assumption

of normal distribution is not met, not even after making an effort of transforming data to normal distribution, logistic regression will be used with grouping variable as dependent variable, change in DDS as main effect variable and DDS score at baseline as covariate.

- Change in the hypoglycaemia confidence scale from baseline to week 18, 32 and 52 between the two treatment groups using the same methodology as for DDS score above
- Score of the DTSQc at week 18 between the two treatment groups using ANCOVA in case assumption of normal distribution is met, or otherwise by using Mann-Whitney U-test
- Score of the DTSQs at week 18, 32 ,52 between the two treatment groups using ANCOVA in case assumption of normal distribution is met, or otherwise by using Mann-Whitney U-test

Achieving HbA1c goal will be analysed between the two treatment groups using multiple logistic regression with HbA1c at baseline as covariate.

All tests will be two-tailed and conducted on 0.05 significance level.
Analyses will be further specified in the Statistical Analysis Plan (SAP) before the database is locked.

The above efficacy analyses will also be performed on the PP-population.

Descriptive statistics will be given for all efficacy variables by treatment group for each visit and for changes from baseline to 18 weeks and 32weeks. For continuous variables descriptive statistics will be given as mean, SD, median, min and max and for categorical variables as number and percentage.

12.5 Statistical Analysis Plan

A SAP that contains a detailed description of all planned analysis will be written and signed before the database is locked.

12.6 Sample Size Calculation

The study will be designed to detect an improvement in HbA1c of 0.4 percentage units from baseline to 18 weeks follow-up. An SD of 0,8 % for change in HbA1c has been assumed for both treatment groups showing that 64 individuals per group are needed to obtain a power of 80% at an alpha-level of 0.05. If accounting for a drop-out rate of 10% 142 individuals will be needed.

12.7 Safety analyses

All safety analyses will be performed on the safety population.

All AE and SAE will be coded using the MedDRA dictionary and tabulated by treatment group. Number of events, number of patients with events and percentage of patients with events will be given for:

- All events
- All SOC-classes
- All PT-codes within each SOC –code.

13. Premature termination of the trial

The Sponsor or the Investigator may decide to stop the trial or part of the trial at any time. If a trial is prematurely terminated or suspended, the Investigator should promptly inform the patients and ensure appropriate therapy and follow-up. Furthermore, the Investigator should promptly inform the IEC (Independent Ethics Committee) and provide a detailed written explanation. The pertinent regulatory authorities should be informed according to national regulations. If changes to the principal features of the confirmatory statistical analyses described in the protocol are required, a protocol amendment must be prepared. Only results from analyses envisaged in the protocol (including amendments) can be regarded as confirmatory.

14. Data Handling and Record Keeping

14.1 Data Collection

Study data will be collected using electronic Case Record Forms (eCRF). No personal identifiers will be recorded in the eCRF but only an anonymous ID number assigned to each subject in the study will be used.

14.2 Data Management

14.2.1 Study Database & Data Entry

An eCRF will be set up for data entry by Investigator site personnel.

14.2.2 Data Validation & Data Clarifications

Data management will periodically run data validation procedures on the data entered into the database. The data validation procedures will be specified in a Data Validation Plan (DVP). Where applicable, data queries will be raised in the eCRF.

14.2.3 Clean File & Database Lock

Once all study data has been collected and entered, and prior to breaking the randomisation codes, the database will be reviewed for completeness, accuracy and consistency. At a formal Clean File meeting the database will be declared locked, after which point the database will be write protected and analysis start.

14.3 Data Retention & Archiving

Study sites should keep study documents and records, including printout copies of the eCRF and CGM records, for 10 years after the study ends. After the study has been closed, printout copies of the eCRF and raw datasets (eCRF, central lab, and CGM data) will be transferred to the Sponsor for archiving in accordance with data archiving requirements.

15. Access to Source Data/Documents

The investigator will permit trial-related monitoring, audits, IRB/EC review, and regulatory inspection(s), providing direct access to source data/documents.

16. Quality Control and Quality Assurance

Site monitoring will be performed by Gothia Forum.

17. Ethics

The trial will be conducted in accordance with the Declaration of Helsinki Ethical Principles for Medical Research Involving Human Subjects. The protocol is subject to review and approval by relevant ethics committee.

17.1 Declaration of Ethical Conduct

This study will be conducted in accordance with the ethical principles that have their origin in the Declaration of Helsinki and that are consistent with Good Clinical Practice (GCP) and the applicable regulatory requirements. It will be conducted in accordance with Good Clinical Practice (GCP) guidelines as required by the following:

1. Declaration of Helsinki, 1964 ("Recommendations Guiding Physicians in Biomedical Research Involving Human Patients"), and all its accepted amendments to date concerning medical research in humans.

2. ICH Guideline for GCP (CPMP/ICH/135/95) of the European Agency for the Evaluation of Medicinal Products, Committee for Proprietary Medicinal Products, International Conference on Harmonisation of Pharmaceuticals for Human Use. (Note for Guidance on Good Clinical Practice, 2002).

3. European Union (EU) Clinical Trials Directive 2001/20/EC on the regulation of clinical trials in the EU and the implementation of GCP.

4. GCP Directive 2005/28/EC

This study will be conducted in accordance with national and local laws (e.g. drug and narcotics laws of the countries where study sites are located).

The Investigator agrees, when signing the protocol, to adhere to the instructions and procedures described in the protocol and to adhere to the principles of ICH Good Clinical Practice to which the protocol conforms as well as all governing local regulations and principles for medical research.

17.2 Ethical Review

Prior to commencement of the trial, the protocol, any amendments, patient information/Informed Consent Form, any other written information to be provided to the patient, SPC, information about payments and compensation available to patient if not mentioned in the subject information, the physician's current CV and/or other documentation evidencing qualifications, and other documents as required by the local Independent Ethics Committee (IEC) should be submitted. The submission letter should clearly identify (by including version number and/or date of the document) which documents have been submitted to the IEC. Written approval/favourable opinion must be obtained from IEC prior to commencement of the trial.

During the trial, the Investigator must promptly report the following to the IEC unexpected SAEs where a causal relationship cannot be ruled out, amendments to the protocol, notes of administrative changes, deviations to the protocol implemented to eliminate immediate hazards to the trial patients, new information that may affect adversely the safety of the patients or the conduct of the trial and other documents as required by the local IRB/IEC.

Amendments must not be implemented before approval/favorable opinion, unless necessary to eliminate hazards to patients.

The Investigator must maintain an accurate and complete record of all submissions made to the IEC. The records should be filed in the physician's Trial File.

In case of early termination of the study, the Investigator should promptly inform the IEC and provide a detailed written explanation. The pertinent regulatory authorities should be informed according to national regulations.

17.3 Subject Information & Consent

Informed consent should be obtained by means of a patient information sheet (PIS) and informed consent form (ICF), prepared in accordance with ICH E6 section 4.8.10 and applicable local regulations, written in non-technical language. The ICF should list all risks associated with the treatment. All subjects will be provided with oral and written information describing the nature and duration of the study and the procedures to be performed. The subject will be asked to sign an ICF prior to any study-specific procedures being performed. No subject can enter the study before his/her informed consent has been obtained. A sample subject ICF used in the study will be included in the clinical study report for this protocol.

As part of administering the ICF, the Investigator must explain to each subject the nature of the study, its purpose, the procedures involved, the expected duration, the potential risks and benefits involved, and any potential discomfort. Each subject must be informed that participation in the

study is voluntary and that he/she may withdraw from the study at any time and that withdrawal of consent will not affect his/her subsequent medical treatment or relationship with the treating physician. The subject should understand the PIS and ICF before signing and dating the ICF. The Investigator or person obtaining consent must also sign and date the form.

The original signed ICF for each subject will be verified by the Sponsor monitor and kept in the study site investigational site files. Each subject will be given a copy of the signed ICF and written information.

18. Protocol Adherence & Amendments

18.1 Adherence to Protocol

The Investigator will conduct the study in strict accordance with the protocol, which has been written to enable the Investigator's compliance with ICH E6, Section 4, "Investigator Guideline for Good Clinical Practices."

There are to be no waivers to inclusion/exclusion criteria and no Investigator-led deviations from the schedules and procedures set out within this protocol. Any subject whose treatment deviates from the protocol or who is not qualified for study participation may be ineligible for analysis and may compromise the study.

Subjects who have not signed an EC approved ICF cannot receive study treatment.

The Investigator and research team must comply with ICH E6 principles and all applicable local regulatory laws and regulations.

18.2 Protocol Amendments

Any changes to the protocol will be made by formal amendment. For changes potentially increasing risks to study participants, approval of a protocol amendment must be obtained from the IRB/EC prior to implementation of the change. Changes required to ensure the immediate safety of study participants may be made prior to IRB/EC approval or notification of the Sponsor, but will require prompt, full IRB/IEC notification and acknowledgement after the fact. Administrative changes not influencing subject treatment must be reported to the IRB/EC in accordance with its procedures and must at least be acknowledged in writing by the chairperson of the IRB/EC ("expedited approval"). The IRB/EC letter approving each protocol modification and identifying both the amendment or administrative change and the date of the meeting at which it was approved must be retained by the Investigator and a copy must be provided to the sponsor. The ICF must be revised to reflect protocol modifications affecting participants' treatment or risks, to provide new information potentially influencing participants' willingness to initiate or continue study participation, or to advise of important administrative changes (e.g., change of addresses, phone numbers, IRB/EC or subject ombudsman contact). Following IRB/EC approval, the revised ICF must be signed in a timely fashion by all current study participants. The earlier versions of IRB/EC-approved ICFs must be archived with study records.

19. Financing and Insurance

A separate financial protocol will be set up. Subjects are insured according to the Swedish patient insurance scheme.

20. Compensation

The subjects will receive travel expenses and present voucher for the value of 800kr for their trouble of participating in the study.

21. Publication Policy

The trial will be posted on http://clinicaltrials.ifpma.org/ before trial start. The results of the trial will be published by the Investigators in an international scientific journal.

22. Supplements/Appendices

22.1 Amendments

Before the Investigator commences the trial, the following documents must be available:

- Regulatory approval and/or notification as required (independent ethics committee
- Signed and dated agreement on the final protocol
- Curricula vitae of the Investigator and Sub-Investigator(s) (current, dated and signed and/or supported by an official regulatory document)

22.2 Personnel Information

The Investigator is responsible for the conduct of the trial. If any tasks are delegated, the Investigator should maintain a list of appropriately qualified persons to whom he/she has delegated specified significant trial-related duties

23. References

1. Lind M, Svensson AM, Kosiborod M, Gudbjornsdottir S, Pivodic A, Wedel H, et al. Glycemic control and excess mortality in type 1 diabetes. N Engl J Med. 2014;371(21):1972-82.
2. Pickup JC, Freeman SC, Sutton AJ. Glycaemic control in type 1 diabetes during real time continuous glucose monitoring compared with self monitoring of blood glucose: meta-analysis of randomised controlled trials using individual patient data. BMJ (Clinical research ed). 2011;343:d3805.
3. Lind M, Polonsky W, Hirsch IB, Heise T, Bolinder J, Dahlqvist S, et al. Continuous Glucose Monitoring vs Conventional Therapy for Glycemic Control in Adults With Type 1 Diabetes Treated With Multiple Daily Insulin Injections: The GOLD Randomized Clinical Trial. Jama. 2017;317(4):379-87.
4. Bolinder J, Antuna R, Geelhoed-Duijvestijn P, Kröger J, Weitgasser R. Novel glucose-sensing technology and hypoglycaemia in type 1 diabetes: a multicentre, non-masked, randomised controlled trial. The Lancet. 2016.
5. Hanås R. Type 1 Diabetes in Children, Adolescents and Young Adults. 6 ed. Uddevalla: BetaMed; 2015.
6. Abbott. FreeStyle Libre 2017. Available from: https://www.freestylelibre.se/.
7. S. Gudbjörnsdóttir A-MS, Björn Eliasson, Katarina Eeg-Olofsson, Pär Samuelsson, Ebba Linder, Mervete Miftaraj. Nationella Diabetesregistret Årsrapport 2016. 2017.
