# Supplementary material for: Systematic intensive therapy in addition to continuous glucose monitoring in adults with type 1 diabetes: a multicentre, open-label, randomised controlled trial
Source: Lancet Reg Health Eur. 2025 Oct 16;59:101485. doi: 10.1016/j.lanepe.2025.101485 (PMC12553072; doi:10.1016/j.lanepe.2025.101485)
Supplement: All Protocol Amendments [file mmc3.pdf]

Protocol Amendments list

1. Addition of site
2. Change within questionnaire
3. Addition of site
4. Safety amendment within STI therapy
5. Addition of site
6. Addition of site
7. Addition of site
8. Sampling size
9. Rewording of objectives and efficacy variables

INKOM

2018 -06- 07

Etikprövningsnämnden i Göteborg

Till

Regionala Etikprövningsnämnden i Göteborg

Uddevalla 2018-05-30

Tillägg till Dnr: 225-18

Avseende studien " Effekten av systematisk intensivterapi på blodsockerkontroll och diabetes distress vid typ 1 diabetes".

Avseende ovanstående studie har vi gjort några justeringar:

Sedan tidigare ansökan har ett studie site lagts till: Universitetssjukhuset Örebro med Erik Schwarcz som prövare, resursintyg är bifogad.

Vänliga hälsningar

30/5-2018

Dr Marcus Lind
Medicinmottagningen
Uddevalla Sjukhus
Uddevalla

Datum

Granskad och godkänd som sekreterarärende
Göteborg
11/6-2018
Staffan Björck, docent, bitr vetenskapl sekr
Avdelning för Medicinsk forskning 1
Regionala Etikprövningsnämnden i Göteborg

Dnr T 547-18
Ad 225-18
Betalt 2018-06-08
Exp 2018-06-12

Protocol Amendment

Number: 2

Study Code: SIT

Date: 2018/09/13

A randomised trial of evaluating a systematic intensive therapy using Continuous Glucose Monitoring (CGM) and Flash Glucose Monitoring (FGM) in clinical diabetes care

Sponsor:

Marcus Lind, MD, PhD, Sahlgrenska Academy, University of Gothenburg, Gothenburg, Sweden and NU-Hospital Organization, Uddevalla, Sweden

**Previous text**							**Revised text**							
9. Trial Procedures							9. Trial Procedures							
	Inclusion visit 1 *	Randomisation visit 2	10 week Follow-up visit 3	18 week follow-up visit 4	32 week follow-up visit 5	52 week follow-up visit 6	Inclusion visit 1 *	Randomisation visit 2	10 week Follow-up visit 3	18 week follow-up visit 4	32 week follow-up visit 5	52 week follow-up visit 6	Inclusion visit 1 *
DTSQc				X	X	X	DTSQc			X			

Section(s) of protocol to be amended:

Previous text	Revised text
9.4 Clinical visits<br><br>Initial visit with a diabetes nurse will take place at the randomisation visit. Treatment will be initiated in accordance with section 7.2 "Treatment". At visit week 18, 32, and 52 with the diabetes nurse CGM/FGM will be downloaded and the following variables will be measured:<br><br>• HbA1c<br>• Weight<br>• Type of insulin and doses of insulin<br>• AE, SAE<br>• DTSQs, DTSQc, DDS, and hypoglycaemia confidence scale<br><br>At visit week 10 with the diabetes nurse CGM/FGM will be downloaded and following variable will be measured:<br><br>• HbA1c<br><br>At week 52 a physical examination will be performed	9.4 Clinical visits<br><br>Initial visit with a diabetes nurse will take place at the randomisation visit. Treatment will be initiated in accordance with section 7.2 "Treatment". At visit week 18, 32, and 52 with the diabetes nurse CGM/FGM will be downloaded and the following variables will be measured:<br><br>• HbA1c<br>• Weight<br>• Type of insulin and doses of insulin<br>• AE, SAE<br>• DTSQs, DTSQc, DDS, and hypoglycaemia confidence scale<br>• Number of contacts (clinical or telephone) with regular diabetes care team<br><br>At visit week 10 with the diabetes nurse CGM/FGM will be downloaded and following variable will be measured:<br><br>• HbA1c<br><br>At week 52 a physical examination will be performed

Reason for Amendment:

1. Changes in Trial Procedures are due to corrections from the questionnaires licensing body
2. Changes in clinical visits are due to the importance of an overview of participants contact with diabetes care teams to be able to analyse the effect of the trial intervention better.

Signed agreement to the Amendment:

I agree to the terms of this Protocol Amendment.

Study Code: SIT

Date<br>(day month year)	Principal investigator<br>Marcus Lind

Till

Uddevalla 2018-11-21

Regionala Etikprövningsnämnden i Göteborg

För kännedom angående studie Dnr: 225-18

Avseende studien " Effekten av systematisk intensivterapi på blodsockerkontroll och diabetes distress vid typ 1 diabetes".

Avseende ovanstående studie har vi gjort några justeringar:

Sedan tidigare ansökan har ett studie site i Australien och ett studie site på Island lagts till:

Steinunn Arnardóttir, MD, Landspítali Háskólasjúkrahús, Reykjavík, Iceland.

Claire Morbey, MD, Hunter Diabetes Center, Newcastle, NSW, Australia.

Etiska tillstånd sökes I nuläget I båda dessa länder.

Vänliga hälsningar

[signature]

Dr Marcus Lind
Medicinmottagningen
Uddevalla Sjukhus
Uddevalla

21 Nov. 2018
Datum

Protocol Amendment

Number:	3
Study Code:	SIT
Date:	2018/11/21

A randomised trial of evaluating a systematic intensive therapy using Continuous Glucose Monitoring (CGM) and Flash Glucose Monitoring (FGM) in clinical diabetes care

Sponsor:

Marcus Lind, MD, PhD, Sahlgrenska Academy, University of Gothenburg, Gothenburg, Sweden and NU-Hospital Organization, Uddevalla, Sweden

Section(s) of protocol to be amended:

Previous text	Revised text
4.5<br><br>Marcus Lind, MD, PhD, Diabetologist, Diabetes Section Uddevalla Hospital, Uddevalla, Sweden<br><br>Ulf Rosengren, MD, Diabetes clinic, Hospital in Motala, Motala, Sweden<br><br>Thomas Nyström, MD, Department of Medicine, Diabetes, Södersjukhuset, Stockholm, Sweden<br><br>Magnus Wijkman, MD, Diabetes Clinic, Vrinnevihospital Norrköping, Norrköping, Sweden<br><br>Per-Anders Jansson MD, Gothia Forum, Sahlgrenska Universitetssjukhus, Göteborg, Sweden<br><br>Erik Schwarcz, MD, Örebro Diabetes clinic, University Hospital, Örebro, Sweden	4.5<br><br>Marcus Lind, MD, PhD, Diabetologist, Diabetes Section Uddevalla Hospital, Uddevalla, Sweden<br><br>Ulf Rosengren, MD, Diabetes clinic, Hospital in Motala, Motala, Sweden<br><br>Thomas Nyström, MD, Department of Medicine, Diabetes, Södersjukhuset, Stockholm, Sweden<br><br>Magnus Wijkman, MD, Diabetes Clinic, Vrinnevihospital Norrköping, Norrköping, Sweden<br><br>Per-Anders Jansson MD, Gothia Forum, Sahlgrenska Universitetssjukhus, Göteborg, Sweden<br><br>Erik Schwarcz, MD, Örebro Diabetes clinic, University Hospital, Örebro, Sweden<br><br>Steinunn Arnardóttir, MD, Landspítali Háskólasjúkrahús, Reykjavík, Iceland.<br><br>Claire Morbey, MD, Hunter Diabetes Center, Newcastle, NSW, Australia

Reason for Amendment:

2 New Site have been added to the Study

Signed agreement to the Amendment:
I agree to the terms of this Protocol Amendment.

Study Code: SIT

2/Nov. 2018

Date
(day month year)

Principal investigator
Marcus Lind

Uddevalla 2019-02-29

Till Etikprövningsnämnden

För kännedom angående studie Dnr: 225-18

Avseende studien ” Effekten av systematisk intensivterapi på blodsockerkontroll och diabetes distress vid typ 1 diabetes”.

Avseende ovanstående studie har vi gjort några justeringar:

Trial Design - Treatments

In sektion 7.2.2 Intensive diabetes care, vi har lagt till följande, om patienten har uppnått medelblodglukos på 8,4mmol/l och behöver inte telefonkontakt för att minska sitt blodglukos så ska vårdgivaren ändå kontakta patienten om det finns ett tydligt mönster med låga blodglukosen värden som behöver åtgärdas. Vi har lagt till detta för att öka säkerheten för patienterna.

Vänliga hälsningar

Dr Marcus Lind
Medicinmottagningen
Uddevalla Sjukhus
Uddevalla

1/3-2019
Datum

Protocol Amendment
Number: 4
Study Code: SIT
Date: 2019/03/01

A randomised trial of evaluating a systematic intensive therapy using Continuous Glucose Monitoring (CGM) and Flash Glucose Monitoring (FGM) in clinical diabetes care

Sponsor:

Marcus Lind, MD, PhD, Sahlgrenska Academy, University of Gothenburg, Gothenburg, Sweden and NU-Hospital Organization, Uddevalla, Sweden

Section(s) of protocol to be amended:

Previous text	Revised text
7.2.2 Intensive diabetes care<br><br>If the patient reaches the recommended goal of mean blood glucose < 8,4 mmol/l no telephone contact will be made that week but data needs to be downloaded again the following week and new contact made if mean glucose has risen above 8,4 mmol/l	7.2.2 Intensive diabetes care<br><br>If the patient reaches the recommended goal of mean blood glucose < 8,4 mmol/l no telephone contact will be made that week but data needs to be downloaded again the following week and new contact made if mean glucose has risen above 8,4 mmol/l. If the care givers notice a pattern of hypoglycaemic values in the downloaded data they are recommended to contact the patients to give appropriate recommendations.

Reason for Amendment:

1. Patient safety.

Signed agreement to the Amendment:
I agree to the terms of this Protocol Amendment.

Study Code: SIT

1/3-2019

Date
(day month year)

Principal investigator
Marcus Lind

Till

Uddevalla 201-08-16

Regionala Etikprövningsnämnden i Göteborg

För kännedom angående studie Dnr: 225-18

Avseende studien " Effekten av systematisk intensivterapi på blodsockerkontroll och diabetes distress vid typ 1 diabetes".

Avseende ovanstående studie har vi gjort några justeringar:

Sedan tidigare ansökan har två studie site lagts till:

Peter Fors, MD, Alingsås Hospital, Alingsås, Sweden

Helen Holmer, MD, Central Hospital, Kristianstad, Sweden

Vänliga hälsningar

Dr Marcus Lind
Medicinmottagningen
Uddevalla Sjukhus
Uddevalla

19/8-2019
Datum

Protocol Amendment

Number:	5
Study Code:	SIT
Date:	2019/08/16

A randomised trial of evaluating a systematic intensive therapy using Continuous Glucose Monitoring (CGM) and Flash Glucose Monitoring (FGM) in clinical diabetes care

Sponsor:

Marcus Lind, MD, PhD, Sahlgrenska Academy, University of Gothenburg, Gothenburg, Sweden and NU-Hospital Organization, Uddevalla, Sweden

Section(s) of protocol to be amended:

Previous text	Revised text
4.5<br><br>Marcus Lind, MD, PhD, Diabetologist, Diabetes Section Uddevalla Hospital, Uddevalla, Sweden<br><br>Ulf Rosengren, MD, Diabetes clinic, Hospital in Motala, Motala, Sweden<br><br>Thomas Nyström, MD, Department of Medicine, Diabetes, Södersjukhuset, Stockholm, Sweden<br><br>Magnus Wijkman, MD, Diabetes Clinic, Vrinnevihospital Norrköping, Norrköping, Sweden<br><br>Per-Anders Jansson MD, Gothia Forum, Sahlgrenska Universitetssjukhus, Göteborg, Sweden<br><br>Erik Schwarcz, MD, Örebro Diabetes clinic, University Hospital, Örebro, Sweden<br><br>Steinunn Arnardóttir, MD, Landspítali Háskólasjúkrahús, Reykjavík, Iceland.<br><br>Claire Morbey, MD, Hunter Diabetes Center, Newcastle, NSW, Australia	4.5<br><br>Marcus Lind, MD, PhD, Diabetologist, Diabetes Section Uddevalla Hospital, Uddevalla, Sweden<br><br>Ulf Rosengren, MD, Diabetes clinic, Hospital in Motala, Motala, Sweden<br><br>Thomas Nyström, MD, Department of Medicine, Diabetes, Södersjukhuset, Stockholm, Sweden<br><br>Magnus Wijkman, MD, Diabetes Clinic, Vrinnevihospital Norrköping, Norrköping, Sweden<br><br>Per-Anders Jansson MD, Gothia Forum, Sahlgrenska Universitetssjukhus, Göteborg, Sweden<br><br>Erik Schwarcz, MD, Örebro Diabetes clinic, University Hospital, Örebro, Sweden<br><br>Steinunn Arnardóttir, MD, Landspítali Háskólasjúkrahús, Reykjavík, Iceland.<br><br>Claire Morbey, MD, Hunter Diabetes Center, Newcastle, NSW, Australia<br><br>Peter Fors, MD, Alingsås Hospital, Alingsås, Sweden<br><br>Helen Holmer, MD, Central Hospital, Kristianstad, Sweden

Reason for Amendment:

2 New Site have been added to the Study

Signed agreement to the Amendment:
I agree to the terms of this Protocol Amendment.

Study Code: SIT

19/8-2019

Date
(day month year)

Principal investigator
Marcus Lind

Resursintyg avseende studien " A randomised trial of evaluating a systematic intensive therapy using Continuous Glucose Monitoring (CGM) and Flash Glucose Monitoring (FGM) in clinical diabetes care"

Härmed intygas att vi har resurser för att garantera forskningspersonernas säkerhet och integritet samt resurser och medel för genomförandet av studien " Effekten av systematisk intensivterapi blodsockerkontroll, diabetesrelaterad stress, behandlingstillfredsställelse och trygghetskänsla kring hypoglykemi".

I tjänsten Datum: 190731

Verksamhetschef
Helene Holmer
Diabetesmottagningen

Resursintyg avseende studien " A randomised trial of evaluating a systematic intensive therapy using Continuous Glucose Monitoring (CGM) and Flash Glucose Monitoring (FGM) in clinical diabetes care"

Härmed intygas att vi har resurser för att garantera forskningspersonernas säkerhet och integritet samt resurser och medel för genomförandet av studien " Effekten av systematisk intensivterapi blodsockerkontroll, diabetesrelaterad stress, behandlingstillfredsställelse och trygghetskänsla kring hypoglykemi".

I tjänsten

Datum:

2019-06-27

Verksamhetschef

Jörgen Andreasson
Cerksamheteschef
Medicinkliniken
Alingsås lasarett

Till Uddevalla 2019-10-24

Etikprövningsmyndigheten

För kännedom angående studie Dnr:2019-02017/ (225-18 – etikprövningsnämnden Göteborg)

Avseende studien " Effekten av systematisk intensivterapi på blodsockerkontroll och diabetes distress vid typ 1 diabetes".

Avseende ovanstående studie har vi gjort några justeringar:

Sedan tidigare ansökan har ett studie site lagts till: Setphan Quittenbaum MD, Medicinkliniken, Centrallasarettet Växjö, Växjö

Vänliga hälsningar

24 Oct. 2019

Dr Marcus Lind
Medicinmottagningen
Uddevalla Sjukhus
Uddevalla

Datum

Protocol Amendment

Number:	6
Study Code:	SIT
Date:	2019/10/24

A randomised trial of evaluating a systematic intensive therapy using Continuous Glucose Monitoring (CGM) and Flash Glucose Monitoring (FGM) in clinical diabetes care

Sponsor:

Marcus Lind, MD, PhD, Sahlgrenska Academy, University of Gothenburg, Gothenburg, Sweden and NU-Hospital Organization, Uddevalla, Sweden

Section(s) of protocol to be amended:

Previous text	Revised text
4.5	4.5
Marcus Lind, MD, PhD, Diabetologist, Diabetes Section Uddevalla Hospital, Uddevalla, Sweden	Marcus Lind, MD, PhD, Diabetologist, Diabetes Section Uddevalla Hospital, Uddevalla, Sweden
Ulf Rosengren, MD, Diabetes clinic, Hospital in Motala, Motala, Sweden	Ulf Rosengren, MD, Diabetes clinic, Hospital in Motala, Motala, Sweden
Thomas Nyström, MD, Department of Medicine, Diabetes, Södersjukhuset, Stockholm, Sweden	Thomas Nyström, MD, Department of Medicine, Diabetes, Södersjukhuset, Stockholm, Sweden
Magnus Wijkman, MD, Diabetes Clinic, Vrinnevihospital Norrköping, Norrköping, Sweden	Magnus Wijkman, MD, Diabetes Clinic, Vrinnevihospital Norrköping, Norrköping, Sweden
Per-Anders Jansson MD, Gothia Forum, Sahlgrenska Universitetssjukhus, Göteborg, Sweden	Per-Anders Jansson MD, Gothia Forum, Sahlgrenska Universitetssjukhus, Göteborg, Sweden
Erik Schwarcz, MD, Örebro Diabetes clinic, University Hospital, Örebro, Sweden	Erik Schwarcz, MD, Örebro Diabetes clinic, University Hospital, Örebro, Sweden
Steinunn Arnardóttir, MD, Landspítali Háskólasjúkrahús, Reykjavík, Iceland.	Steinunn Arnardóttir, MD, Landspítali Háskólasjúkrahús, Reykjavík, Iceland.
Claire Morbey, MD, Hunter Diabetes Center, Newcastle, NSW, Australia	Claire Morbey, MD, Hunter Diabetes Center, Newcastle, NSW, Australia
Peter Fors, MD, Alingsås Hospital, Alingsås, Sweden	Peter Fors, MD, Alingsås Hospital, Alingsås, Sweden
Helen Holmer, MD, Central Hospital, Kristianstad, Sweden	Helen Holmer, MD, Central Hospital, Kristianstad, Sweden
	Setphan Quittenbaum, Medicinkliniken, Centrallasarettet Växjö, Växjö

Reason for Amendment:

1 New Site have been added to the Study

Signed agreement to the Amendment:
I agree to the terms of this Protocol Amendment.

Study Code: SIT

24 oct. 2019

Date<br>(day month year)	Principal investigator<br>Marcus Lind

Protocol Amendment

Number:	6
Study Code:	SIT
Date:	2019/11/4

A randomised trial of evaluating a systematic intensive therapy using Continuous Glucose Monitoring (CGM) and Flash Glucose Monitoring (FGM) in clinical diabetes care

Sponsor:

Marcus Lind, MD, PhD, Sahlgrenska Academy, University of Gothenburg, Gothenburg, Sweden and NU-Hospital Organization, Uddevalla, Sweden

Sections(s) of protocol to be amended:

4.5 **Previous text**	4.5 **Amendet text**
Marcus Lind, MD, PhD, Diabetologist, Diabetes Section Uddevalla Hospital, Uddevalla, Sweden<br><br>Ulf Rosengren, MD, Diabetes clinic, Hospital in Motala, Motala, Sweden<br><br>Thomas Nyström, MD, Department of Medicine, Diabetes, Södersjukhuset, Stockholm, Sweden<br><br>Magnus Wijkman, MD, Diabetes Clinic, Vrinnevihospital Norrköping, Norrköping, Sweden<br><br>Per-Anders Jansson MD, Gothia Forum, Sahlgrenska Universitetssjukhus, Göteborg, Sweden<br><br>Erik Schwarcz, MD, Örebro Diabetes clinic, University Hospital, Örebro, Sweden<br><br>Steinunn Arnardóttir, MD, Landspítali Háskólasjúkrahús, Reykjavík, Iceland.<br><br>Claire Morbey, MD, Hunter Diabetes Center, Newcastle, NSW, Australia<br><br>Peter Fors, MD, Alingsås Hospital, Alingsås, Sweden<br><br>Helen Holmer, MD, Central Hospital, Kristianstad, Sweden	Marcus Lind, MD, PhD, Diabetologist, Diabetes Section Uddevalla Hospital, Uddevalla, Sweden<br><br>Ulf Rosengren, MD, Diabetes clinic, Hospital in Motala, Motala, Sweden<br><br>Thomas Nyström, MD, Department of Medicine, Diabetes, Södersjukhuset, Stockholm, Sweden<br><br>Magnus Wijkman, MD, Diabetes Clinic, Vrinnevihospital Norrköping, Norrköping, Sweden<br><br>Per-Anders Jansson MD, Gothia Forum, Sahlgrenska Universitetssjukhus, Göteborg, Sweden<br><br>Erik Schwarcz, MD, Örebro Diabetes clinic, University Hospital, Örebro, Sweden<br><br>Steinunn Arnardóttir, MD, Landspítali Háskólasjúkrahús, Reykjavík, Iceland.<br><br>Claire Morbey, MD, Hunter Diabetes Center, Newcastle, NSW, Australia<br><br>Peter Fors, MD, Alingsås Hospital, Alingsås, Sweden<br><br>Helen Holmer, MD, Central Hospital, Kristianstad, Sweden<br><br>Per-Henrik Nilsson, Medicinkliniken, Centrallasarettet Växjö, Växjö<br><br>Lacramioara Grosu, Medicin/Rehab, Kiruna sjukhus, Kiruna<br><br>Ana Popescu, Medicinkliniken, Skaraborgs sjukhus Skövde, Skövde<br><br>Jesper Fowelin, Medicinmottagningen, Kungsbacka Sjukhus, Kungsbacka

Reason for Amendment:

3 New Site have been added to the Study

Signed agreement to the Amendment:
I agree to the terms of this Protocol Amendment.

Study Code: SIT

20/11-2019

Date<br>(day month year)	Principal investigator<br>Marcus Lind

Tillägg till tidigare inskickat ändringsansökan

Till

Uddevalla 2019-11-06

Etikprövningsmyndigheten

För kännedom angående studie Dnr:2019-02017/ (225-18 – etikprövningsnämnden Göteborg)

Avseende studien ” Effekten av systematisk intensivterapi på blodsockerkontroll och diabetes distress vid typ 1 diabetes”.

Avseende ovanstående studie har vi gjort några justeringar:

Sedan tidigare ansökan har 3 studie site lagts till:

Per-Henrik Nilsson, Medicinkliniken, Centrallasarettet Växjö, Växjö

Lacramioara Grosu, Medicin/Rehab, Kiruna sjukhus, Kiruna

Ana Popescu, Medicinkliniken, Skaraborgs sjukhus Skövde, Skövde

Jesper Fowelin, Medicinmottagningen, Kungsbacka Sjukhus, Kungsbacka

Vänliga hälsningar

Dr Marcus Lind
Medicinmottagningen
Uddevalla Sjukhus
Uddevalla

20/11-2019
Datum

Protocol Amendment

Number:	6a
Study Code:	SIT
Date:	2019/12/12

A randomised trial of evaluating a systematic intensive therapy using Continuous Glucose Monitoring (CGM) and Flash Glucose Monitoring (FGM) in clinical diabetes care

Sponsor:

Marcus Lind, MD, PhD, Sahlgrenska Academy, University of Gothenburg, Gothenburg, Sweden and NU-Hospital Organization, Uddevalla, Sweden

Sections(s) of protocol to be amended:

4.5 **Previous text**	4.5 **Amendet text**
Marcus Lind, MD, PhD, Diabetologist, Diabetes Section Uddevalla Hospital, Uddevalla, Sweden	Marcus Lind, MD, PhD, Diabetologist, Diabetes Section Uddevalla Hospital, Uddevalla, Sweden
Ulf Rosengren, MD, Diabetes clinic, Hospital in Motala, Motala, Sweden	Ulf Rosengren, MD, Diabetes clinic, Hospital in Motala, Motala, Sweden
Thomas Nyström, MD, Department of Medicine, Diabetes, Södersjukhuset, Stockholm, Sweden	Thomas Nyström, MD, Department of Medicine, Diabetes, Södersjukhuset, Stockholm, Sweden
Magnus Wijkman, MD, Diabetes Clinic, Vrinnevihospital Norrköping, Norrköping, Sweden	Magnus Wijkman, MD, Diabetes Clinic, Vrinnevihospital Norrköping, Norrköping, Sweden
Per-Anders Jansson MD, Gothia Forum, Sahlgrenska Universitetssjukhus, Göteborg, Sweden	Per-Anders Jansson MD, Gothia Forum, Sahlgrenska Universitetssjukhus, Göteborg, Sweden
Erik Schwarcz, MD, Örebro Diabetes clinic, University Hospital, Örebro, Sweden	Erik Schwarcz, MD, Örebro Diabetes clinic, University Hospital, Örebro, Sweden
Steinunn Arnardóttir, MD, Landspítali Háskólasjúkrahús, Reykjavík, Iceland.	Steinunn Arnardóttir, MD, Landspítali Háskólasjúkrahús, Reykjavík, Iceland.
Claire Morbey, MD, Hunter Diabetes Center, Newcastle, NSW, Australia	Claire Morbey, MD, Hunter Diabetes Center, Newcastle, NSW, Australia
Peter Fors, MD, Alingsås Hospital, Alingsås, Sweden	Peter Fors, MD, Alingsås Hospital, Alingsås, Sweden
Helen Holmer, MD, Central Hospital, Kristianstad, Sweden	Helen Holmer, MD, Central Hospital, Kristianstad, Sweden
Per-Henrik Nilsson, Medicinkliniken, Centrallasarettet Växjö, Växjö	Per-Henrik Nilsson, Medicinkliniken, Centrallasarettet Växjö, Växjö
Lacramioara Grosu, Medicin/Rehab, Kiruna sjukhus, Kiruna	Lacramioara Grosu, Medicin/Rehab, Kiruna sjukhus, Kiruna
Ana Popescu, Medicinkliniken, Skaraborgs sjukhus Skövde, Skövde	Ana Popescu, Medicinkliniken, Skaraborgs sjukhus Skövde, Skövde
Jesper Fowelin, Medicinmottagningen, Kungsbacka Sjukhus, Kungsbacka	Jesper Fowelin, Medicinmottagningen, Kungsbacka Sjukhus, Kungsbacka

	Kari-Anne Sveen, Endokrinologisk Poliklinikk, Oslo Universitetssykehus HF, Oslo, Norge<br><br>Marianne Höglund, Sörlandet Sykehus HF, Arendal, Norge

Reason for Amendment:

2 New Site have been added to the Study

Signed agreement to the Amendment:
I agree to the terms of this Protocol Amendment.

Study Code: SIT

7Jan2020

Date<br>(day month year)	Principal investigator<br>Marcus Lind

Protocol Amendment

Number:	7
Study Code:	SIT
Date:	2021/08/09

A randomised trial of evaluating a systematic intensive therapy using Continuous Glucose Monitoring (CGM) and Flash Glucose Monitoring (FGM) in clinical diabetes care

Sponsor:

Marcus Lind, MD, PhD, Sahlgrenska Academy, University of Gothenburg, Gothenburg, Sweden and NU-Hospital Organization, Uddevalla, Sweden

Section(s) of protocol to be amended:

Previous text	Revised text
4.5<br><br>Marcus Lind, MD, PhD, Diabetologist, Diabetes Section Uddevalla Hospital, Uddevalla, Sweden<br><br>Ulf Rosengren, MD, Diabetes clinic, Hospital in Motala, Motala, Sweden<br><br>Thomas Nyström, MD, Department of Medicine, Diabetes, Södersjukhuset, Stockholm, Sweden<br><br>Magnus Wijkman, MD, Diabetes Clinic, Vrinnevihospital Norrköping, Norrköping, Sweden<br><br>Per-Anders Jansson MD, Gothia Forum, Sahlgrenska Universitetssjukhus, Göteborg, Sweden<br><br>Erik Schwarcz, MD, Örebro Diabetes clinic, University Hospital, Örebro, Sweden<br><br>Steinunn Arnardóttir, MD, Landspítali Háskólasjúkrahús, Reykjavík, Iceland.<br><br>Claire Morbey, MD, Hunter Diabetes Center, Newcastle, NSW, Australia<br><br>Peter Fors, MD, Alingsås Hospital, Alingsås, Sweden<br><br>Helen Holmer, MD, Central Hospital, Kristianstad, Sweden<br><br>Setphan Quittenbaum, Medicinkliniken, Centrallasarettet Växjö, Växjö	4.5<br><br>Marcus Lind, MD, PhD, Diabetologist, Diabetes Section Uddevalla Hospital, Uddevalla, Sweden<br><br>Ulf Rosengren, MD, Diabetes clinic, Hospital in Motala, Motala, Sweden<br><br>Thomas Nyström, MD, Department of Medicine, Diabetes, Södersjukhuset, Stockholm, Sweden<br><br>Magnus Wijkman, MD, Diabetes Clinic, Vrinnevihospital Norrköping, Norrköping, Sweden<br><br>Per-Anders Jansson MD, Gothia Forum, Sahlgrenska Universitetssjukhus, Göteborg, Sweden<br><br>Erik Schwarcz, MD, Örebro Diabetes clinic, University Hospital, Örebro, Sweden<br><br>Steinunn Arnardóttir, MD, Landspítali Háskólasjúkrahús, Reykjavík, Iceland.<br><br>Claire Morbey, MD, Hunter Diabetes Center, Newcastle, NSW, Australia<br><br>Peter Fors, MD, Alingsås Hospital, Alingsås, Sweden<br><br>Helen Holmer, MD, Central Hospital, Kristianstad, Sweden<br><br>Setphan Quittenbaum, Medicinkliniken, Centrallasarettet Växjö, Växjö<br><br>Sara Hallström, Forskningsenheten, Östra sjukhuset, Sahlgrenska, Göteborg

Reason for Amendment:

1 New Site have been added to the Study

Signed agreement to the Amendment:
I agree to the terms of this Protocol Amendment.

Study Code: SIT

Date<br>(day month year)	Principal investigator<br>Marcus Lind

Protocol Amendment	
Number:	8
Study Code:	SIT
Date:	2022/01/24

A randomised trial of evaluating a systematic intensive therapy using Continuous Glucose Monitoring (CGM) and Flash Glucose Monitoring (FGM) in clinical diabetes care

Sponsor:

Marcus Lind, MD, PhD, Sahlgrenska Academy, University of Gothenburg, Gothenburg, Sweden and NU-Hospital Organization, Uddevalla, Sweden

Section(s) of protocol to be amended:

Previous text	Revised text
**5.2 Study Population**<br><br>The study will be performed at 6 clinics in Sweden including a total of 142 individuals. Patients with type 1 diabetes with HbA1c ≥ 58 mmol/mol, currently using CGM or FGM and who have the possibility to download their devices at home will be included. Patients will be recruited at each site.<br><br>**8 Selection and Withdrawal of Subjects**<br><br>The study is planned to include 142 subjects randomised 1:1 to systematic intensive care or conventional care. Drop-outs (expected to be maximally 10%) will not be replaced.	**5.2 Study Population**<br><br>The study will be performed at 6 clinics in Sweden including a total of 120 individuals. Patients with type 1 diabetes with HbA1c ≥ 58 mmol/mol, currently using CGM or FGM and who have the possibility to download their devices at home will be included. Patients will be recruited at each site.<br><br>**8 Selection and Withdrawal of Subjects**<br><br>The study is planned to include 120 subjects randomised 1:1 to systematic intensive care or conventional care. Drop-outs (expected to be maximally 10%) will not be replaced.

Section(s) of protocol to be amended:

Previous text	Revised text
**12.6 Sample Size Calculation**<br><br>The study will be designed to detect an improvement in HbA1c of 0.4 percentage units from baseline to 18 weeks follow-up. An SD of 0,8 % for change in HbA1c has been assumed for both treatment groups showing that 64 individuals per group are needed to obtain a power of 80% at an alpha-level of 0.05. If accounting for a drop-out rate of 10% 142 individuals will be needed.	**12.6 Sample Size Calculation**<br><br>The study will be designed to detect an improvement in HbA1c of 0.435 percentage units from baseline to 18 weeks follow-up. An SD of 0,8 % for change in HbA1c has been assumed for both treatment groups showing that 54 individuals per group are needed to obtain a power of 80% at an alpha-level of 0.05. If accounting for a drop-out rate of 10% 120 individuals will be needed.

Reason for Amendment:

Due to the current pandemic, recruitment has been exceptionally difficult, therefore the study size has been reduced to some extent but will still detect relatively small differences in HbA1c of 0.435% with a power 80%.

Signed agreement to the Amendment:

I agree to the terms of this Protocol Amendment.

Study Code: SIT

24 Jan 2022

Date
(day month year)

Principal investigator
Marcus Lind

**Protocol Amendment**	
Number:	9
Study Code:	SIT
Date:	2023/11/24

A randomised trial of evaluating a systematic intensive therapy using Continuous Glucose Monitoring (CGM) and Flash Glucose Monitoring (FGM) in clinical diabetes care

Sponsor:

Marcus Lind, MD, PhD, Sahlgrenska Academy, University of Gothenburg, Gothenburg, Sweden and NU-Hospital Organization, Uddevalla, Sweden

Section(s) of protocol to be amended:

Previous text	Revised text
**6.3 Secondary Objectives**<br>Secondary objectives are comparison of the following variables between patients with type 1 diabetes randomized to systematic intensive therapy or conventional care:<br>• HbA1c at 32 weeks<br>• HbA1c at 52 weeks<br>• Mean glucose levels at 18, 32, and 52 weeks.<br>• Glycaemic variability by Standard Deviation, CV and MAGE at 18, 32, and 52 weeks.<br>• Time in Hypoglycaemia at 18, 32, and 52 weeks,<br>• Time in Hyperglycaemia 18, 32, and 52 weeks<br>• Hypoglycaemia Confidence (Hypoglycaemia confidence scale) at 18, 32, and 52 weeks<br>• Diabetes distress (DDS-questionnaire) at 18, 32, and 52 weeks<br>• Treatment satisfaction (DTSQs and c questionnaire) at 18, 32, and 52 weeks	**6.3 Secondary Objectives**<br>Secondary objectives are comparison of the following variables between patients with type 1 diabetes randomized to systematic intensive therapy or conventional care:<br>• Time in Range (TIR) at week 18<br>• Mean glucose at week 18<br>• Time above range (TAR) at week 18<br>• HbA1c at week 32<br>• HbA1c at week 52<br><br>Exploratory objectives are comparison of the following variables between patients with type 1 diabetes randomized to systematic intensive therapy or conventional care:<br>• Mean glucose week 32 and 52<br>• TBR week 18, 32 and 52<br>• TAR week 32 and 52<br>• Glycaemic variability by standard deviation, CV and Mage week 18, 32 and 52<br>• DTSQ at week 18, 32 and 52<br>• Hypoglycaemic confidence week 18, 32 and 52<br>• Diabetes distress week 18, 32 and 52

<table>
<tr><th>Previous text</th><th>Revised text</th></tr>
<tr><td>7.5.2 Secondary
Secondary endpoints will be:
change in HbA1c from baseline to week 32
change in HbA1c from baseline to week 52
Change in mean glucose levels from baseline to 18, 32, and 52 weeks
Change in glycaemic variability from baseline to 18, 32, and 52 weeks
Change in time in hypoglycaemia from baseline to 18, 32, and 52 weeks
Change in time in hyperglycaemia from baseline to 18, 32, and 52 weeks
Change in Hypoglycaemic confidence score from baseline to 18, 32, and 52 weeks
Change in DDS score from baseline to 18, 32, and 52 weeks
Change in DTSQc score from baseline to 18, 32, and 52 weeks</td><td>7.5.2 Secondary
Secondary endpoints will be:
Change in Time in Range (TIR) from baseline to week 18
Change in Mean glucose from baseline to week 18
Change in Time above range (TAR) from baseline to week 18
Change in HbA1c from baseline to week 32
Change in HbA1c from baseline to week 52
Exploratory endpoints will be:
Change in Mean glucose from baseline to week 32 and 52
Change in TBR from baseline to week 18, 32 and 52
Change in TAR from baseline to week 32 and 52
Change in Glycaemic variability by standard deviation, CV and Mage from baseline to week 18, 32 and 52
Change in DTSQ from baseline to week 18, 32 and 52
Change in Hypoglycaemic confidence from baseline to week 18, 32 and 52
Change in Diabetes distress from baseline to week 18, 32 and 52</td></tr>
<tr><td>11.3 Secondary efficacy variables
Secondary efficacy variables:
change in HbA1c from baseline to week 32
change in HbA1c from baseline to week 52
Change in mean glucose levels from baseline to 18, 32, and 52 weeks
Change in glycaemic variability from baseline to 18, 32, and 52 weeks
Change in time in hypoglycaemia from baseline to 18, 32, and 52 weeks
Change in time in hyperglycaemia from baseline to 18, 32, and 52 weeks
Change in Hypoglycaemic confidence score from baseline to 18, 32, and 52 weeks
Change in DDS score from baseline to 18, 32, and 52 weeks
Change in DTSQs score from baseline to 18, 32, and 52 weeks and DTSQc at 18, 32 and 52 weeks.</td><td>11.3 Secondary efficacy Variables
Secondary efficacy variables will be change in:
Time in Range (TIR) from baseline to week 18
Mean glucose from baseline to week 18
Time above range (TAR) from baseline to week 18
HbA1c from baseline to week 32
HbA1c from baseline to week 52
Exploratory efficacy variables will be change in:
Mean glucose from baseline to week 32 and 52
TBR from baseline to week 18, 32 and 52
TAR from baseline to week 32 and 52
Glycaemic variability by standard deviation, CV and Mage from baseline to week 18, 32 and 52
DTSQ from baseline to week 18, 32 and 52
Hypoglycaemic confidence from baseline to week 18, 32 and 52
Diabetes distress from baseline to week 18, 32 and 52</td></tr>
</table>

Reason for Amendment: In preparation for final collection of data from study sites and further on performing the statistical analysis plan it was decided to reorganize several of the secondary endpoints to exploratory endpoints since earlier a very large number of secondary endpoints existed. This was performed to accommodate for confirmative testing.

Signed agreement to the Amendment:

I agree to the terms of this Protocol Amendment.

Study Code: SIT

8Dec2023 [illegible]

____

Date<br>(day month year)	Principal investigator<br>Marcus Lind
