# Supplementary material for: Systematic intensive therapy in addition to continuous glucose monitoring in adults with type 1 diabetes: a multicentre, open-label, randomised controlled trial
Source: Lancet Reg Health Eur. 2025 Oct 16;59:101485. doi: 10.1016/j.lanepe.2025.101485 (PMC12553072; doi:10.1016/j.lanepe.2025.101485)
Supplement: SIT SAP [file mmc4.pdf]

Statistical Analysis Plan

FINAL

Systematic Intensive Therapy (SIT)

A randomised trial of evaluating a systematic intensive therapy using Continuous Glucose Monitoring (CGM) and intermittent-scanning Continuous Glucose Monitoring (isCGM) in clinical diabetes care

25 March 2024

Author

Henrik Imberg / Principal Statistician, Statistiska Konsultgruppen Sweden AB

....
Signature: Date

Approvals

Marcus Lind / Principal Investigator, Professor of Diabetology, University of Gothenburg, Senior consultant of diabetes, NU-Hospital Group and Sahlgrenska University Hospital/Östra, Sweden

....
Signature: Date

Arndís Finna Ólafsdóttir, University of Gothenburg, Sahlgrenska University Hospital/Östra and NU Hospital Group

....
Signature: Date

Revisions

Version	Description	Date
0.1	First draft	11 March 2024
1.0	Final version	25 March 2024

Table of Contents

1 Study details ... 5
1.1 Study objectives ... 5
1.2 Study design ... 5
1.3 Treatment groups ... 6
1.4 Sample size ... 6
2 Analysis populations ... 6
2.1 Intention-to-treat (ITT) population ... 6
2.2 Per-Protocol (PP) population ... 6
2.3 Safety population ... 6
3 Study variables ... 7
3.1 Demographics and baseline characteristics ... 7
3.2 Endpoints ... 7
3.2.1 Primary endpoint ... 7
3.2.2 Secondary endpoints ... 7
3.2.3 Exploratory endpoints ... 7
3.3 Safety variables ... 8
3.3.1 Safety endpoints ... 8
3.3.2 Adverse events ... 8
4 Statistical methods ... 8
4.1 General statistical methodology ... 8
4.2 Patient disposition and datasets analysed ... 9
4.3 Protocol violations ... 9
4.4 Demographics and baseline characteristics ... 9
4.5 Efficacy analyses ... 9
4.5.1 Primary efficacy analysis ... 9
4.5.2 Secondary efficacy analyses ... 9
4.5.3 Exploratory efficacy analyses ... 10
4.5.4 Compliance ... 10
4.6 Safety analyses ... 10
5 Changes of analysis from protocol ... 10
6 List of Tables and Figures ... 10
6.1 List of Tables ... 10
6.2 List of Figures ... 11

ABBREVIATIONS

Acronym	Definition
AE	Adverse event
ANCOVA	Analysis of covariance
ATC	Anatomical therapeutic classification
CGM	Continuous glucose monitoring
CSII	Continuous subcutaneous insulin infusion
CV	Coefficient of variation
DDS	Diabetes distress scale
DTSQ	Diabetes treatment satisfaction questionnaire
DTSQc	Diabetes treatment satisfaction questionnaire, change version
DTSQs	Diabetes treatment satisfaction questionnaire, status version
eCRF	Electronic case report form
HbA1c	Haemoglobin A1c
HCS	Hypoglycaemia confidence scale
ICD	International statistical classification of diseases and health related problems
isCGM	Intermittent-scanning continuous glucose monitoring
ITT	Intention-to-treat
MAGE	Mean amplitude of glycaemic excursions
MDI	Multiple daily insulin injections
PP	Per-protocol
SAE	Serious adverse event
SIT	Systematic intensive therapy
SD	Standard deviation
TAR	Time above range
TBR	Time below range
TIR	Time in range
TITR	Time in tight range

1 STUDY DETAILS

1.1 Study objectives

The primary objective is to evaluate whether systematic intensive therapy in combination with isCGM and CGM with regular telephone-based counselling by diabetes care teams improves glycaemic control, measured by the change in HbA1c, compared to conventional care with CGM and isCGM supported by advice only at regular clinical visits, in persons with type 1 diabetes with impaired glycaemic control, during an 18-week treatment period.

Secondary objectives are to evaluate changes in the following variables between patients with type 1 diabetes randomised to systematic intensive therapy compared to conventional care:

- Time in range (TIR, 3.9–10 mmol/L) after 18 weeks of treatment,
- Mean glucose at 18 weeks,
- Time above range (TAR, >10 mmol/L) at 18 weeks,
- HbA1c 32 and 52 weeks after randomisation.

Exploratory objectives are to evaluate further changes in glycaemic control, diabetes treatment satisfaction, hypoglycaemia confidence and diabetes distress in patients with type 1 diabetes randomised to systematic intensive therapy compared to conventional care at 18, 32 and 52 weeks after randomisation.

1.2 Study design

Multi-centre, open-label, parallel-arm, randomised clinical trial over 52-weeks across eight outpatient clinics in Sweden and Norway. Treatment allocation was performed using minimisation with balancing on sex, age, HbA1c level, treatment type (injections or pump), and sensor type (CGM/isCGM). The trial procedures are schematically shown below. Before each visit, patient information was given via telephone or at a clinical visit.

Variable	Visit 1*† Inclusion	Visit 2† Randomisation	Visit 3 10-week follow-up	Visit 4 18-week follow-up	Visit 5 32-week follow-up	Visit 6 52-week follow-up
Visit window		Within 28 days after inclusion	±1 weeks	±1 weeks	±2 weeks	±2 weeks
Informed consent	X					
Inclusion/exclusion criteria	X					
Demographics, medical history	X					
Physical examination	X					X
HbA1c	X	X	X	X	X	X
Download data from CGM device	X	X	X	X	X	X
Education on downloading data from CGM device		X				
Weight		X		X	X	X
DTSQs		X		X	X	X
DTSQc				X		
Diabetes distress scale		X		X	X	X
Hypoglycaemia confidence scale		X		X	X	X
AE (severe hypoglycaemia and diabetes ketoacidosis), SAE		X	X	X	X	X

*If randomised to systematic intensive treatment, the first telephone contact will take place 1 week after randomisation and after that on a weekly basis or until mean glucose levels reach target.
†Visit 1 (inclusion) and 2 (randomisation) often done at the same clinical visit.
Abbreviations: AE, adverse event; DTSQc, diabetes treatment satisfaction questionnaire, change version; DTSQs, diabetes treatment satisfaction questionnaire, status version; HbA1c, haemoglobin A1c; SAE, serious adverse event.

1.3 Treatment groups

Patients were randomised to Conventional treatment or Systematic Intensive Therapy (SIT).

The SIT group received 18 weeks of regular telephone consultations with their diabetes team, supporting with advice regarding improving glucose control by interpreting CGM data and educating patients by distance contacts. If the glucose control was on target a certain week, no distance counselling was performed that week. The conventional treatment group came for regular clinical visits during this time. Both groups attended 2 additional follow-up visits with the last visit being one year after randomisation.

1.4 Sample size

The study was designed to detect an improvement in HbA1c of 0.435% (4.75 mmol/mol) after 18 weeks of treatment with systematic intensive therapy compared to conventional treatment. An SD of 0.8% (8.75 mmol/mol) for the change in HbA1c was assumed for both treatment groups, showing that 54 individuals per group were needed to obtain a power of 80% at significance level α=0.05. Accounting for a drop-out rate of 10%, 120 individuals were needed in total.

2 ANALYSIS POPULATIONS

2.1 Intention-to-treat (ITT) population

All randomised subjects will be included in the Intent-to-treat (ITT) population. The patient will be analysed by the randomised group irrespective of treatment received.

2.2 Per-Protocol (PP) population

All randomised subjects with no major protocol violations will be included in the Per-Protocol (PP) population(s). Two per-protocol populations will be analysed.

PP1 comprises all randomised subjects satisfying the following criteria:

- Attended at least 11 (70%) of the telephone contact appointments (SIT group only). If the glucose control was on target at a certain visit, no contact was scheduled at that occasion according to the protocol. Such an occasion will therefore be regarded as the protocol was followed although no distance counselling took place.
- Attended visit week 10 and 18, with HbA1c measurement available at week 18.
- Attended visit week 18 within ±3 weeks from the scheduled visit.

PP2 comprises all randomised subjects satisfying the following criteria:

- Attended at least 11 (70%) of the telephone contact appointments (SIT group only). See further details regarding definition of 70% contacts in PP1 above.
- Attended visit week 10, 18, 32 and 52, with HbA1c measurement available at week 18, 32 and 52.
- Attended each visit within the following windows:
    - Visit week 18 within ±3 weeks from the scheduled visit,
    - Visit week 32 within ±4 weeks from the scheduled visit,
    - Visit week 52 within ±5 weeks from the scheduled visit.

The PP-populations are defined at the clean-file meeting before the database is locked. Individuals excluded from the PP1- and PP2-populations will be listed, including the reason(s) for exclusion.

2.3 Safety population

All randomised individuals will be included in the safety population. In the safety analysis, a patient will belong to the treatment given, not to the randomised treatment.

3 STUDY VARIABLES

3.1 Demographics and baseline characteristics

- Age, years
- Sex
- HbA1c, mmol/mol and %
- Weight, kg
- Height, cm
- Body mass index (BMI), kg/m^2
- Smoking (yes/no)
- Glucose sensor type (Enlite Medtronic 640G, Enlite Veo, Dexcom G4, Dexcom G5, Eversense, FreeStyle Libre, Other)
- Diabetes duration, years
- Diabetes treatment satisfaction questionnaire, status version (DTSQs), total scale
- Diabetes distress scale (DDS)
- Hypoglycaemic confidence scale
- Total bolus insulin dose/day, IU
- Total basal insulin dose/day, IU
- Total daily insulin dose, IU
- Insulin delivery (continuous subcutaneous insulin infusion [CSII], multiple daily injections [MDI])

3.2 Endpoints

3.2.1 Primary endpoint

The primary endpoint is the difference in change in HbA1c (mmol/mol) from baseline to 18-weeks follow-up between treatment groups.

3.2.2 Secondary endpoints

Secondary endpoints are the differences between treatment groups regarding changes in

- Percentage of time in range (TIR, 3.9–10 mmol/L) from baseline to week 18,
- Mean glucose (mmol/L) from baseline to week 18,
- Percentage of time above range (TAR, >10 mmol/L), from baseline to week 18,
- HbA1c (mmol/mol) from baseline to week 32,
- HbA1c (mmol/mol) from baseline to week 52.

Mean glucose from the eCRF will be used if raw CGM-data are not available.

3.2.3 Exploratory endpoints

Exploratory endpoints are the differences between treatment groups with regards to:

- Change in percentage of time in range (3.9–10 mmol/L) from baseline to week 32 and 52
- Change in mean glucose (mmol/L) from baseline to week 32 and 52,
- Change in percentage of time below range (TBR, <3.9 mmol/L) from baseline to week 18, 32 and 52,

- Change in percentage of time above range (TAR, >10 mmol/L) from baseline to week 32 and 52,
- Change in glycaemic variability measured by SD (mmol/L) and CV (%) of glucose values, and MAGE (mmol/L), from baseline to week 18, 32 and 52,
- Change in diabetes treatment satisfaction measured by DTSQc at week 18,
- Change in diabetes treatment satisfaction measured DTSQs from baseline to week 18, 32 and 52,
- Change in hypoglycaemic confidence scale (HCS) from baseline to week 18, 32 and 52,
- Change in diabetes distress scale (DDS) from baseline to week 18, 32 and 52,
- Change in percentage of time in tight range (TITR, 3.9–7.8 mmol/L) from baseline to week 18, 32 and 52,
- Change in TBR level 2 (<3.0 mmol/L) from baseline to week 18, 32, and 52, and
- Change in TAR level 2 (>13.9 mmol/L) from baseline to week 18, 32 and 52.

Mean and SD of glucose values from the eCRF will be used if raw CGM-data are not available.

3.3 Safety variables

3.3.1 Safety endpoints

The following safety endpoints will be evaluated by treatment group over the entire study period:

- Any severe hypoglycaemia, and
- Any ketoacidosis.

3.3.2 Adverse events

Adverse events are recorded by duration, severity, action, causality, outcome, and seriousness.

4 STATISTICAL METHODS

4.1 General statistical methodology

Descriptive data will be presented using the mean and standard deviation for numeric variables and using numbers and percentages for categorical variables.

Efficacy analyses will be performed by using analysis of covariance (ANCOVA) on the change from baseline, adjusting for the baseline value. Robust standard errors (HC3 method) will be employed for non-normal variables TAR, TBR, DTSQs, HCS, and DDS. DTSQc will be analysed using the two-sample T-test as baseline variables are not available. Treatment effects will be presented as adjusted mean differences with 95% confidence intervals.

Missing data will be handled using multiple imputation by chained equations, with baseline values, minimisation variables (age, sex, treatment type [CSII or MDI], and sensor type [CGM/isCGM]) and outcomes at earlier/later visits as auxiliary variables for the imputation (using, e.g., HbA1c at baseline, 10, 32 and 52 weeks to impute missing values for HbA1c at 18 weeks). If a baseline value is missing but data from the screening visit is available, this will be used instead. DTSQc will be imputed using DTSQs. 50 imputed datasets will be generated. Missing data will be imputed using imputation for monotone missing data in case of dropouts and fully conditional specification otherwise. Regression imputation will be used for continuous variables (HbA1c, TIR, TITR, TBR, TAR, mean glucose, SD and CV of glucose values, and MAGE) and predictive mean matching for discrete variables (DTSQ, HCS, and DDS). TBR, TBR level 2, TAR and TAR level 2 will be imputed with a lower bound at 0. A fixed seed 636221 of the random number generator will be used for the imputation.

Binary variables (exploratory and safety analyses) will be compared between groups using the Farrington-Manning test for the rate difference (test of equality). The difference in proportions will be presented with corresponding 95% Farrington-Manning confidence intervals.

All efficacy analyses will be performed on the ITT and PP-populations. All tests will be two-tailed and conducted at significance level $\alpha=0.05$. To account for multiple testing, a sequential testing procedure

will be employed. In case of a significant test for the primary endpoint, the entire probability mass α=0.05 will be transferred to the secondary endpoints in the order listed. The procedure will continue until the first encounter of a non-significant test. All these significant tests will be considered confirmatory findings.

Statistical analyses will be performed by using SAS/STAT® Software, Version 9.4 of the SAS System for Windows (SAS Institute Inc. Cary, NC).

4.2 Patient disposition and datasets analysed

The number of subjects included in each of the ITT, PP and safety populations will be summarised for each treatment group and overall. The number and percentage of subjects randomised and treated will be presented. Subjects who completed the study and subjects who withdrew from study prematurely will also be presented with a breakdown of the reasons for withdrawal by treatment group for the ITT, PP, and safety populations.

4.3 Protocol violations

Major protocol deviations are those that are considered to influence the analysis. A list of potential major protocol deviations will be generated programmatically from the data captured before the clean file meeting.

The number of patients with major protocol deviations will be summarised per treatment group.

4.4 Demographics and baseline characteristics

Demographics and baseline characteristics will be summarised by treatment group for the ITT and PP populations. Descriptive data will be presented using the mean and standard deviation for numeric variables and using numbers and percentages for categorical variables.

4.5 Efficacy analyses

4.5.1 Primary efficacy analysis

The primary estimand is the adjusted mean difference in the primary endpoint change in HbA1c from baseline to 18-weeks follow-up between systematic intensive therapy and conventional treatment on the ITT population using analysis of covariance, adjusted for baseline HbA1c, with missing data handled using multiple imputation, as described in **Section 4.1 General statistical methodology**. The primary efficacy analysis will be performed at significance level α=0.05.

Summaries and figures will be presented for the ITT and PP populations. Descriptive data will be presented using the unadjusted sample mean and sample standard deviation at baseline and after 18 weeks of treatment, and change from baseline to 18 weeks, by treatment group. The adjusted mean difference in change from baseline will be presented with corresponding 95% confidence interval.

4.5.2 Secondary efficacy analyses

Secondary efficacy analyses will be performed on the ITT population using analysis of covariance, as described in in **Section 4.1 General statistical methodology**, with respect to the secondary endpoints change in percentage of time in range (TIR, 3.9–10 mmol/L) from baseline to week 18, change in mean glucose (mmol/L) from baseline to week 18, change in percentage of time above range (TAR, >10 mmol/L) from baseline to week 18, and change in HbA1c (mmol/mol) from baseline to week 32 and from baseline to week 52.

In case of a significant test for the primary endpoint, the entire probability mass α=0.05 will be transferred to the secondary endpoints in the order listed. The procedure will continue until the first encounter of a non-significant test. All these significant tests will be considered confirmatory findings.

Summaries and figures will be presented for the ITT and PP populations. Descriptive data will be presented using the unadjusted sample means and sample standard deviations at baseline and

follow-up, and changes from baseline to follow-up, by treatment group. The adjusted mean differences in changes from baseline will be presented with corresponding 95% confidence intervals.

4.5.3 Exploratory efficacy analyses

Exploratory efficacy analyses will be conducted on the ITT and PP populations using analysis of covariance according to the methods described in **Section 4.1 General statistical methodology**. DTSQc will be analysed using the two-sample T-test as baseline values are not available.

Summaries and figures will be presented for the ITT and PP populations. Descriptive data will be presented using the unadjusted sample means and sample standard deviations at baseline and follow-up, and change from baseline to follow-up, by treatment group. The adjusted mean differences in changes from baseline will be presented with corresponding 95% confidence intervals.

The number and proportion of patients that satisfies the following criteria will be summarised descriptively by treatment group at 18, 32 and 52 weeks:

- HbA1c <53 mmol/mol
- TIR ≥70%
- HbA1c ≥60 mmol/mol
- HbA1c ≥70 mmol/mol
- Improvement in Hba1c ≥0.5 or ≥1.0 mmol/mol from baseline to follow-up
- Improvement in TIR ≥5 or ≥10% from baseline to follow-up.

4.5.4 Compliance

Compliance will be summarised as the number of telephone contacts (SIT group only) and visits attended. Summaries will be presented for the ITT population.

4.6 Safety analyses

Safety endpoints (any severe hypoglycaemia, any ketoacidosis) will be compared between systematic intensive therapy and conventional treatment using the Farrington-Manning test for the rate difference. Differences in proportions will be presented with accompanying 95% confidence intervals.

The number of adverse events and number of patients with adverse events will be summarised by treatment group on the safety population by duration, severity, action, causality, outcome, and seriousness.

Summaries and figures will be presented for the safety population.

5 CHANGES OF ANALYSIS FROM PROTOCOL

The following changes from the protocol have been made during the preparation of the statistical analysis plan, prior to database lock:

- Imputation using last observation carried forward (LOCF) replaced by multiple imputation.
- Non-parametric tests for instrument scales (DDS, DTSQ, HCS) replaced by parametric tests for compatibility with the imputation method.

6 LIST OF TABLES AND FIGURES

6.1 List of Tables

Table number	Table title
14.1.1	Patient disposition and data sets analysed (ITT population)
14.1.2.1	Protocol deviations leading to exclusion from PP1 population (ITT population)
14.1.2.2	Protocol deviations leading to exclusion from PP2 population (ITT population)
14.1.3.1	Demographics and baseline characteristics (ITT population)
14.1.3.2	Demographics and baseline characteristics (PP1 population)

14.1.3.3 Demographics and baseline characteristics (PP2 population)
14.2.1.1 Primary, secondary, and exploratory efficacy analyses (ITT population)
14.2.1.2 Primary, secondary, and exploratory efficacy analyses (PP1 population)
14.2.1.3 Primary, secondary, and exploratory efficacy analyses (PP2 population)
14.3.1 Compliance (ITT population)
14.3.2.1 Summary of adverse events (safety population)
14.3.2.2 Adverse events, by system organ class and preferred term (safety population)
14.3.2.3 Adverse events, by system organ class, preferred term, and maximum reported intensity (safety population)
14.3.2.4 Adverse events, by system organ class, preferred term, and causality assessment (safety population)
14.3.2.5 Serious adverse events, by system organ class and preferred term (Safety Population)
14.3.3 Adverse events leading to discontinuation, by system organ class and preferred term (safety population)

6.2 List of Figures

Figure number	Figure title
14.2.1	Line graph with error bars for the mean change (95% CI) in Hba1c from baseline to follow-up during the study period (ITT population).
14.2.2	Line graph with error bars for the mean change (95% CI) in TIR (3.9–10 mmol/L) from baseline to follow-up during the study period (ITT population).
14.2.3	Line graph with error bars for the mean change (95% CI) in mean glucose from baseline to follow-up during the study period (ITT population).
14.2.4	Line graph with error bars for the mean change (95% CI) in TAR (>10 mmol/L) from baseline to follow-up during the study period (ITT population).
14.2.5	Bar plot of patients improving ≥0.5 or ≥1 mmol/mol in HbA1c from baseline to 18 weeks (ITT population)
14.2.6	Bar plot of patients improving ≥0.5 or ≥1 mmol/mol in HbA1c from baseline to 52 weeks (ITT population)
14.2.7	Bar plot of patients improving ≥5 or ≥10% in TIR (3.9–10 mmol/L) from baseline to 18 weeks (ITT population)
14.2.8	Bar plot of patients improving ≥5 or ≥10% in TIR (3.9–10 mmol/L) from baseline to 52 weeks (ITT population)
14.3.1	Bar plot of occurrence of severe hypoglycaemias, ketoacidosis, or serious adverse events during the study period (safety population)
