# Supplementary material for: Systematic intensive therapy in addition to continuous glucose monitoring in adults with type 1 diabetes: a multicentre, open-label, randomised controlled trial
Source: Lancet Reg Health Eur. 2025 Oct 16;59:101485. doi: 10.1016/j.lanepe.2025.101485 (PMC12553072; doi:10.1016/j.lanepe.2025.101485)
Supplement: SIT SAP Signed [file mmc5.pdf]

Statistical Analysis Plan

FINAL

Systematic Intensive Therapy (SIT)

A randomised trial of evaluating a systematic intensive therapy using Continuous Glucose Monitoring (CGM) and intermittent-scanning Continuous Glucose Monitoring (isCGM) in clinical diabetes care

25 March 2024

Author

Henrik Imberg / Principal Statistician, Statistiska Konsultgruppen Sweden AB

Henrik Imberg

Signature

26 March 2024

Date

Approvals

Marcus Lind / Principal Investigator, Professor of Diabetology, University of Gothenburg, Senior consultant of diabetes, NU-Hospital Group and Sahlgrenska University Hospital/Östra, Sweden

Marcus Lind

Signature

25 March 2024

Date

Arndís Finna Ólafsdóttir, University of Gothenburg, Sahlgrenska University Hospital/Östra and NU Hospital Group

Arndís F. Ólafsd.

Signature

26 march 2024

Date
