# Supplementary material for: Systematic intensive therapy in addition to continuous glucose monitoring in adults with type 1 diabetes: a multicentre, open-label, randomised controlled trial
Source: Lancet Reg Health Eur. 2025 Oct 16;59:101485. doi: 10.1016/j.lanepe.2025.101485 (PMC12553072; doi:10.1016/j.lanepe.2025.101485)
Supplement: SIT SAP Amendment [file mmc6.pdf]

Statistical Analysis Plan Amendment:
Clarification on definition of CGM-endpoints

Amendment to:

Systematic Intensive Therapy (SIT): A randomised trial of evaluating a systematic intensive therapy using Continuous Glucose Monitoring (CGM) and intermittent-scanning Continuous Glucose Monitoring (isCGM) in clinical diabetes care, SAP Version 1.0, 25 March 2024

14 May 2024

Author

Henrik Imberg / Principal Statistician, Statistiska Konsultgruppen Sweden AB

Signature: .... Date 22 May 2024

Approvals

Marcus Lind / Principal Investigator, Professor of Diabetology, University of Gothenburg, Senior consultant of diabetes, NU-Hospital Group and Sahlgrenska University Hospital/Östra, Sweden

Signature: .... Date 19 May 2024

Arndís Finna Ólafsdóttir, University of Gothenburg, Sahlgrenska University Hospital/Östra and NU Hospital Group

Signature: .... Date 22 may 2024

ABBREVIATIONS

Acronym	Definition
CGM	Continuous glucose monitoring
eCRF	Electronic case report form
SD	Standard deviation

SECONDARY AND EXPLORATORY CGM-ENDPOINTS

For CGM-endpoints (SAP Section *3.2.2 Secondary endpoints* and *3.2.3 Exploratory endpoints*), a minimum of three days with active CGM is required; the endpoints will be considered as missing otherwise. Up to 30 calendar days of CGM before each study visit will be used. The mean and SD of glucose values will be used as recorded in the eCRF.
